# Correction: Intensive Care Weaning (iCareWean) protocol on weaning from mechanical ventilation: a single-blinded multicentre randomised control trial comparing an open-loop decision support system and routine care, in the general intensive care unit

**DOI:** 10.1136/bmjopen-2020-042145corr1

**Published:** 2020-10-23

Vizcaychipi MP, Martins L, White JR, *et al*. Intensive Care Weaning (iCareWean) protocol on weaning from mechanical ventilation: a single-blinded multicentre randomised control trial comparing an open-loop decision support system and routine care, in the general intensive care unit. *BMJ Open* 2020;10:e042145. doi: 10.1136/bmjopen-2020-042145.

The authors want to alert readers to the following correction made to the published version.

The affiliation of Dan Stleper Karbing and Steve Rees has been updated to: Respiratory and Critical Care Group (rcare), Department of Health Science and Technology, Aalborg University, Denmark.

