# Supplementary material for: Intensive Care Weaning (iCareWean) protocol on weaning from mechanical ventilation: a single-blinded multicentre randomised control trial comparing an open-loop decision support system and routine care, in the general intensive care unit
Source: BMJ Open. 2020 Sep 2;10(9):e042145. doi: 10.1136/bmjopen-2020-042145 (PMC7470506; doi:10.1136/bmjopen-2020-042145)
Supplement: Supplementary data [file bmjopen-2020-042145supp001.pdf]

SUPPLEMENTARY MATERIAL

The following is supplementary material for:

The iCareWean protocol on weaning from mechanical ventilation: A Single-blinded multi-centre randomised control trial comparing an open-loop decision support system and routine care, in the general Intensive Care Unit.

M. P. Vizcaychipi[1,5*],L. Martins[1], J.R. White[1], D.S Karbing[2], , A Gupta[3], S. Singh[1,5], L Osman[1], J. Moreno-Cuesta[4], S.E Rees[2].

1. Magill Department of Anaesthesia, Intensive Care Medicine and Pain Management, Chelsea and Westminster Hospital, C&W NHS Foundation Trust, London, UK.
2. Respiratory and Critical Care group (rcare). Department of Health Science and Technology. Aalborg University, Denmark.
3. Department of Anaesthesia and Intensive Care Medicine, West Middlesex University Hospital, C&W NHS Foundation Trust, London, UK
4. Department of Intensive Care Medicine, North Middlesex University Hospital, London, UK
5. Academic Department of Anaesthesia, Intensive Care & Pain Management, Imperial College London, UK.

**Corresponding Author: Dr Marcela P. Vizcaychipi, email: m.vizcaychipi@imperial.ac.uk*

The purpose of this supplementary material is to provide the reader with an understanding of the structure and function of the Beacon Caresystem©, described here as the clinical decision support system (CDSS). The material includes four sections describing: the mathematical models included in the system, including description of the models used to describe the effects of PEEP; the use of the system; a section illustrating the presentation of advice to the clinician; and a section illustrating screens relating to SBT and the extubation checklist. Much of the material presented here, including patient examples is taken from supplementary material from previous publications (1,2).

Mathematical models included in the CDSS

Figure E1 illustrates the structure of the mathematical models with a full description of model formulation and evaluation published recently (1,2,3). The system includes mathematical models of pulmonary gas exchange; respiratory mechanics; acid-base chemistry of blood, interstitial fluid, tissues and cerebral spinal fluid; respiratory drive and ventilation. In addition, the models include the effects of PEEP on gas exchange, pulmonary mechanics and ventilation as illustrated in figure E2 and described below. All models are tuned to the individual patient's physiological status through measurement of respiratory gas flows and pressures; calorimetry and capnography measurements of respiratory gas fractions of O_2 and CO_2, and subsequent calculation of oxygen utilisation (VO_2) and carbon dioxide production (VCO_2); pulse oximetry measurement of arterial oxygen saturation; and arterial blood measurements of acid-base, oxygenation and haemoglobin fractions. The model of pulmonary gas exchange is tuned to the appropriate matching of ventilation and perfusion to account for O_2 and CO_2 differences between arterial and end tidal gas values. To do so an arterial blood gas (ABG) is required on system start up. In some patients, the system also requires modification of FIO_2 to two levels for 2-5 minutes at each level to tune the pulmonary gas exchange model to the patient, a procedure previously called the automatic lung parameter estimator (ALPE) technique(3,4).

The respiratory mechanics model is tuned to dynamic compliance. The model of acid-base chemistry of the blood is tuned to measured values of arterial pH, PCO_2, PO_2, and SO_2, and haemoglobin concentration, with the acid-base chemistry of the cerebrospinal fluid (CSF) regulated to arterial bicarbonate values to account for chemical changes in respiratory drive. The respiratory drive model is tuned to describe the relationship between alveolar ventilation (VA) and arterial acid-base and oxygenation status. The ventilation model is tuned to the measured serial dead space (V*ds*) to allow calculation of alveolar ventilation. A series of algorithms are present in the CDSS to re-tune the models as new measurements present, or if the patient condition changes. These models are used to simulate the effect of changes in ventilator settings, with the system generating advice based upon simulated values.

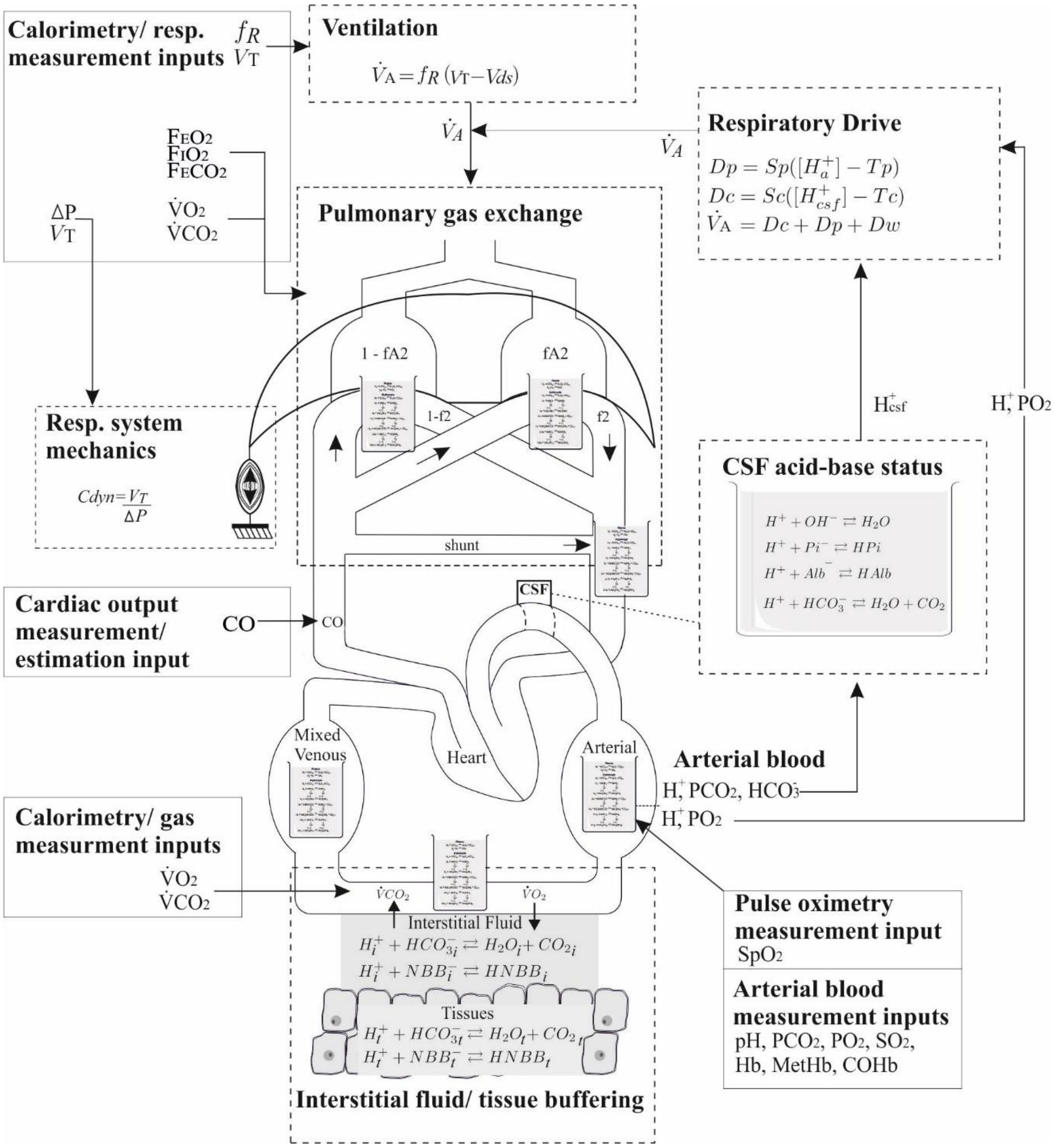

Figure E1: Mathematical models included in the CDSS, modified from (3). Mathematical models are indicated by dashed boxes with the exception of the acid-base model of blood, which is illustrated in each blood compartment as used. Measured input values are indicated by solid boxes.

Vizcaychipi MP, *et al. BMJ Open* 2020; 10:e042145. doi: 10.1136/bmjopen-2020-042145

Figure E2 illustrates the mathematical models built to describe the effects of PEEP. These are formulated as empirical linear models, with the system only advising on PEEP changes of a maximum of 3 cmH_2O in any step and the slope of these relationships modified automatically on measuring the response to PEEP. These models are integrated with those in figure E1 to enable simulation of the patient specific effects of PEEP and other ventilator settings simultaneously, enabling the system to advise on changing settings toward the best compromise for the individual patient given the model simulations.

For patients with acute respiratory failure, PEEP is usually set to improve gas exchange and lung mechanics, and the sub-figures A, B and C of figure E2 illustrate the baseline expected response to changes in PEEP of pulmonary shunt, respiratory system compliance and high ventilation/perfusion (V/Q). Changes in shunt, compliance and high V/Q automatically result in model-simulated changes in oxygenation, ventilation volumes and pressures, and carbon dioxide partial pressures through the models illustrated in figure E1. For shunt and compliance, the initial slopes of the models are dependent on the initial levels of shunt and compliance, with a greater improvement expected for a greater severity of respiratory abnormality. The slopes of these models are adapted automatically according to patient response to changes in PEEP, from measurements of oxygenation (SpO_2), respiratory volumes and pressures, and end tidal CO_2 values. Any increase in end tidal CO_2 values that are simulated to result in severe acidosis result in the system requesting an arterial blood gas. The decision to increase PEEP is then based on the potential benefit of improved oxygenation and respiratory volumes and pressures, given the tuning of the models to the patient's state. For patients responding poorly to increases in PEEP, the absolute slope of shunt and compliance models would be substantially reduced following measurement of the response to PEEP changes. The decision to reduce PEEP at high SpO_2 levels depends upon the FIO_2 level. FIO_2 will always be optimized first, such that the competing goals of sufficient oxygenation and oxygen toxicity are balanced. Following this, the system will calculate the likely effects on oxygenation and respiratory volumes and pressures on reducing PEEP from the models illustrated on subfigures A, B and C of figure E2, combined with the models of E1. For patients recovering from respiratory injury, without substantial pulmonary shunt

or low respiratory system compliance, the system will calculate that the potential negative effects of reducing PEEP will be outweighed by the positive effects of reducing plateau pressure.

For patients on pressure support ventilation without substantial problems related to gas exchange or respiratory system mechanics, PEEP is often used to support the respiratory muscles, preventing abnormal respiratory muscle activity as indicated by abnormal breathing patterns. Figure E2, D and E represent the empirical linear models included in the system to account for changes in tidal volume (VT) due to PEEP. Figure E2D illustrates two potential abnormal situations; the first is a very high VT at a low PEEP value, and the second a very low VT at a high PEEP, possibly representing the situation where the diaphragm may be over distended due to high PEEP. In these situations, the initial slope of the PEEP versus VT relationship is negative. Figure E2E illustrates the situation of a low tidal volume at a low value of PEEP, possible due to under-support of the respiratory muscles. In this situation, the initial slope of the PEEP versus VT relationship is positive. As with other PEEP models, all slopes are adapted following measurement of the response to PEEP changes.

Vizcaychipi MP, *et al. BMJ Open* 2020; 10:e042145. doi: 10.1136/bmjopen-2020-042145

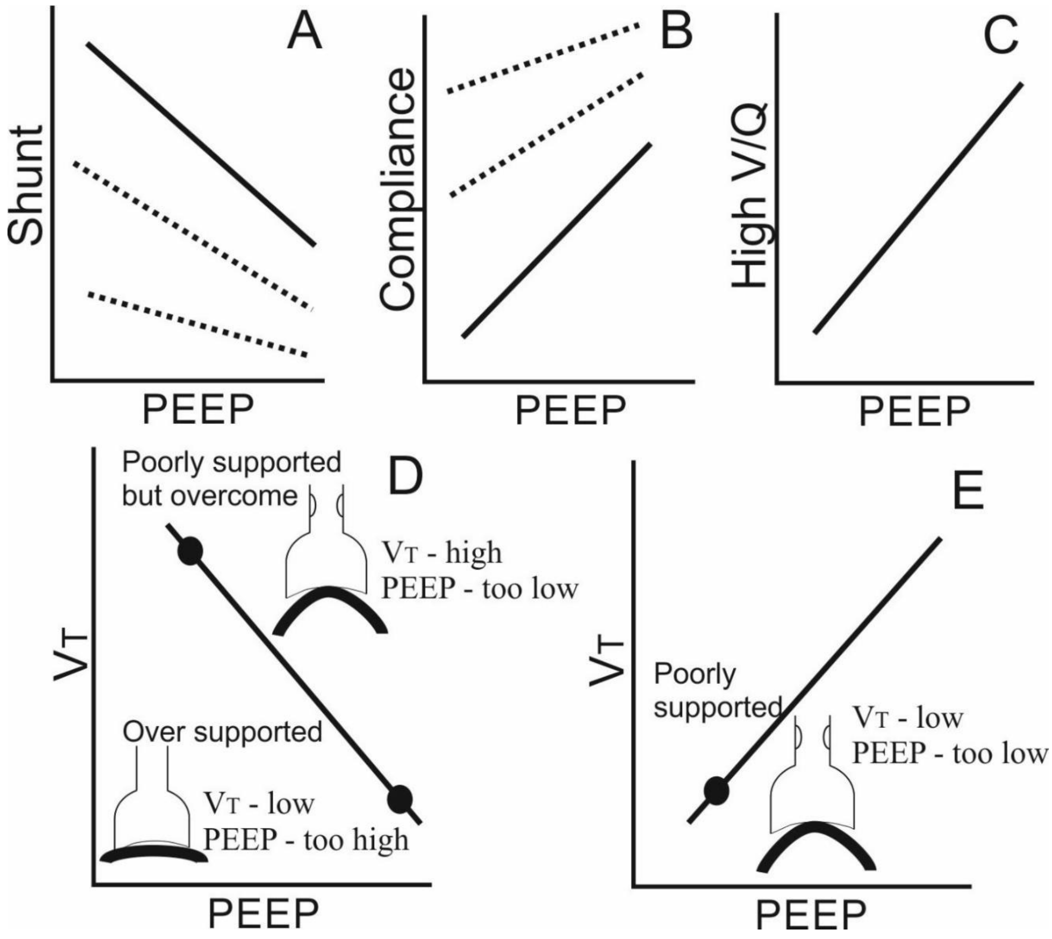

Figure E2: Mathematical models describing PEEP included in the Beacon Caresystem.

Use of the system

The system includes a tablet computer, plus a gas module including volumetric capnography and indirect calorimetry, with both mounted on the ventilator or a separate trolley. The patient is connected to the system via a respiratory tube measuring flow and with side stream sampling for measurement of O_2 and CO_2. The following text includes examples of advice generated by the system as reported in [1,2] beginning with the most simple examples. These examples are presentations of the screen of the tablet computer shown to the user.

In addition to these examples, it is important to note that on occasion the system will present advice to switch ventilator mode. This occurs if the patient is in a controlled ventilator mode and the system identifies a total respiratory frequency or a total minute ventilation that is significantly greater than that set, where the system provides a message to the user suggesting changing to support mode. Similarly, if the patient is in support mode and the model simulated pH is very low then the system provides a message asking the user to consider either control mode or reduction of anesthesia. In modes containing mandatory breaths initiated by the ventilator or by patient effort, and where different settings are available for patient initiated and ventilator initiated breaths, the system provides advice on these depending upon the pattern of patient ventilation, and whether the patient is currently in a period with substantial spontaneous breathing activity. The system functions on a variety of manufactures ventilators and, as illustrated in these examples, provides advice for a variety of modes. Current exceptions to these include APRV, and modes with automatic control of ventilator settings such as ASV© or SmartCare©. Data is collected automatically by the Beacon Caresystem, and includes tidal volumes, both set if the mode dictates, and also measured. The regulation of Vt and it relationship with mode can therefore be explored for both of the study arms. The system records all ventilator settings via connection to the ventilator, all direct measurements of flow and gas concentrations, its own SpO_2 measurement, and the timing and nature of all advice provided. The

extent to which advice has been followed and the effects of application of advice is therefore fully traceable.

Vizcaychipi MP, *et al. BMJ Open* 2020; 10:e042145. doi: 10.1136/bmjopen-2020-042145

Advice in pressure support mode ventilation.

Figure E3 illustrates a typical screen of the CDSS with advice to reduce PS and FIO_2. The hexagon on the right hand side of the screen visualizes the patient state in terms of the balances between over and under ventilation. The three vertical axes on the hexagon present the three balances between over and under oxygenation on the far right, (i.e. the compromise between the risks of oxygen toxicity and hypoxaemia, respectively); over and under ventilation as the central axis (i.e. the compromise between the risks of ventilator induced lung trauma and acidosis, respectively); and on the left the balance between over and under support (i.e. the balance between respiratory muscle atrophy due to over support, or patient stress due to under support). The blue symbol on the hexagon represents the patient's current state, and the grey symbol the simulated state according to the system's advice. The colour of the hexagon represents severity, with the most severe state represented as red, and the system automatically zooming in to yellow and green when possible. The left hand side of the screen shows both the current, simulated and advised settings. The white simulated blocks on the left are scroll wheels which allow the user to perform simulations of other levels of these ventilator settings.

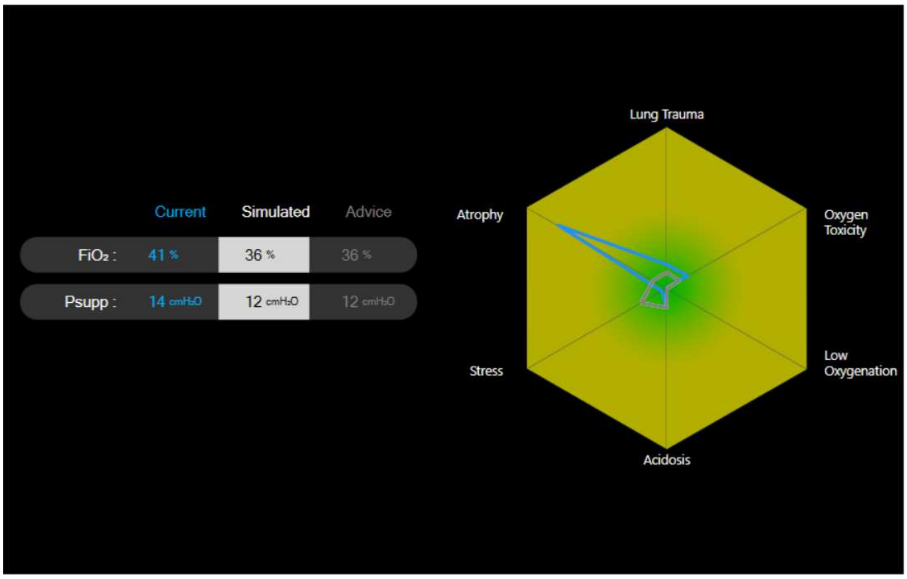

Figure E3 – User interface of the system, with advice to reduce FIO_2 and PS.

Figure E4 illustrates the same screen as E3, but with the corners of the hexagon activated to show the current, simulated and advised values of variables simulated by the physiological models. In this patient, the current FIO_2 setting of 41 %, results in a SaO_2 value of 97.9 % and a current simulated SvO_2 of 89.7 %. The system illustrates that the current balance between over and under oxygenation may be inappropriate with the blue symbol on the hexagon pointing slightly toward oxygen toxicity. The system therefore suggests reducing FIO_2. The system also illustrates an increased risk of respiratory muscle atrophy due to a low respiratory frequency, leading the system to suggest a reduction in PS from 14 to 12 cmH_2O, simulating that this may result in increased Rf.

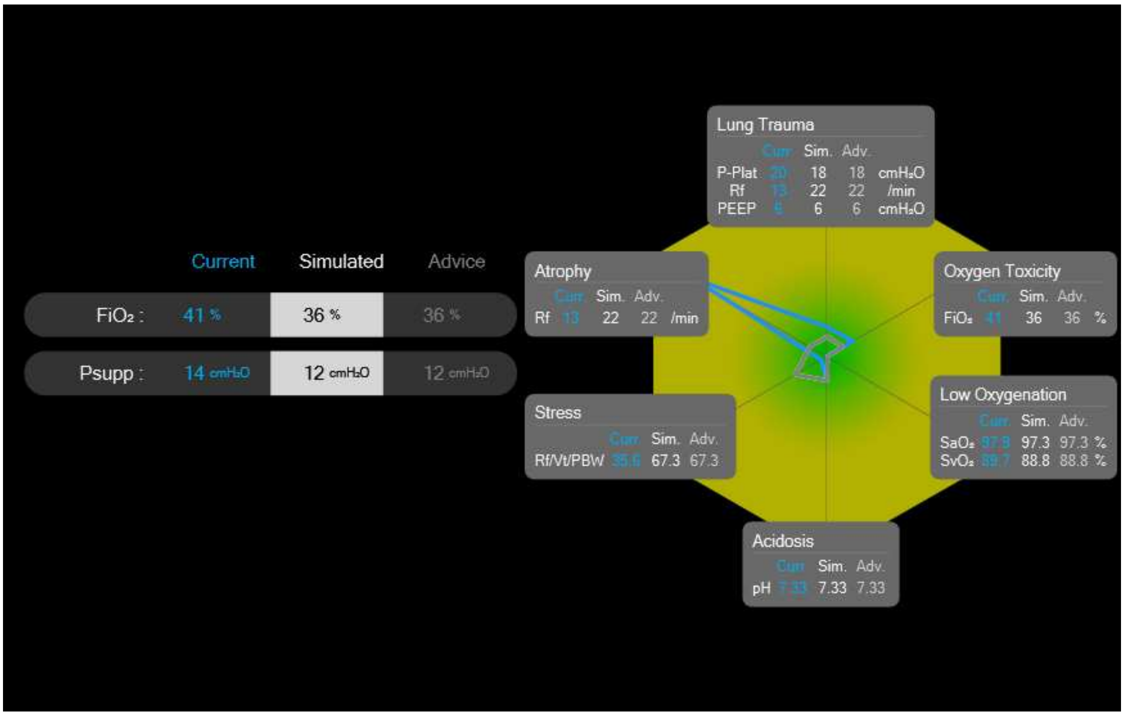

Figure E4 – User interface of the system, with advice to reduce FIO_2 and PS and corners of the hexagon activated to show model simulated physiological variables associated with the different conflicting clinical goals of mechanical ventilation.

Vizcaychipi MP, *et al. BMJ Open* 2020; 10:e042145. doi: 10.1136/bmjopen-2020-042145

Following several reductions in PS and FIO_2, the system advises on PEEP, illustrated in figure E5. The reduction of PS had less than the expected effect on increasing Rf, with the reduction in FIO_2 reducing oxygenation as expected. When exploring advice on PEEP, the CDSS also shows the simulated expected effect of PEEP, showing the simulated levels of shunt, low V/Q (ΔPO_2), high V/Q (ΔPCO_2), dead space (Vd) and dynamic compliance (COMP) at current, simulated and advised settings. The PEEP reduction was expected to have a limited effect on gas exchange parameters, with small increase in shunt, and a small decrease in dynamic compliance. As such, the advice was expected to result in a slight increase in Rf without a worsening in oxygenation.

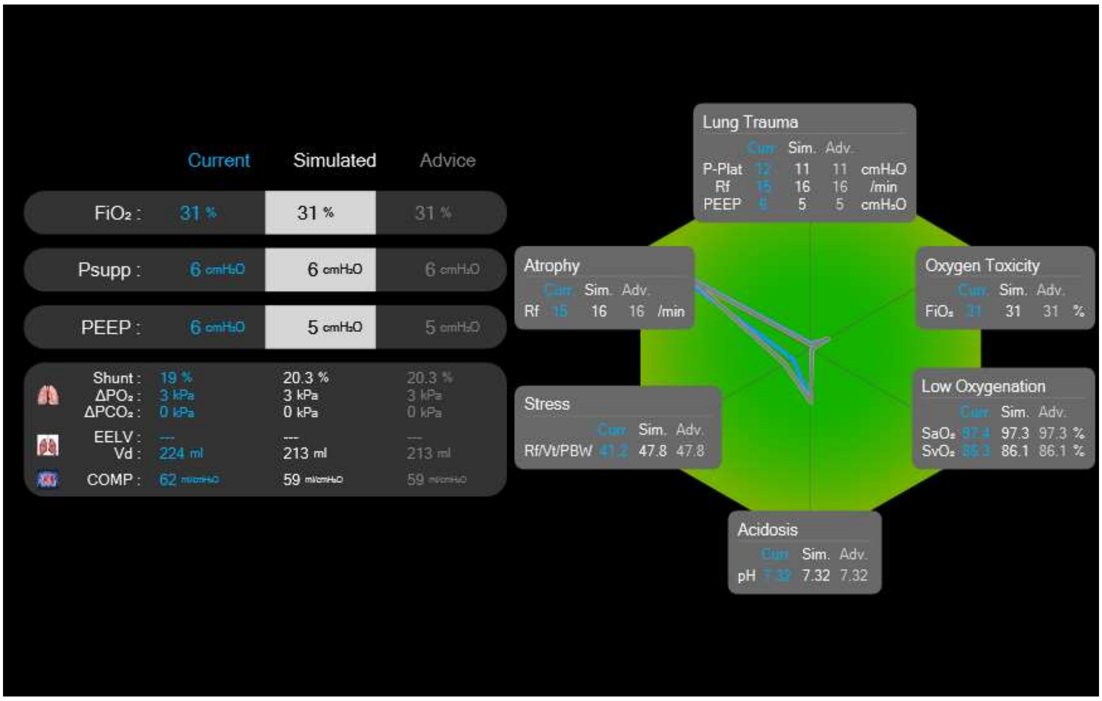

Figure E5 – User interface of the system, with an advice on PEEP.

Vizcaychipi MP, *et al. BMJ Open* 2020; 10:e042145. doi: 10.1136/bmjopen-2020-042145

Advice in control mode ventilaiton.

Figure E6 illustrates an advice including PEEP for a patient in pressure control ventilation. The vertical axis representing the balance between respiratory muscle atrophy and stress is disabled in controlled ventilation modes for cases where the patient has no spontaneous breathing activity. In this patient, the system indicated that the current balance between over and under ventilation might be inappropriate, with the blue symbol on the hexagon pointing toward lung trauma and with the patient being slightly alkalotic. The system suggested reducing PEEP and PC from 6 to 5 cmH_2O and 12 to 11 cmH_2O, respectively, and at the same time increasing Rf from 18 to 19 bpm. The simulated effect of PEEP was a negligible increase in pulmonary shunt, with simulated patient response to the combined advice being to reduce inspiratory pressure, reduce pH and maintain appropriate oxygenation.

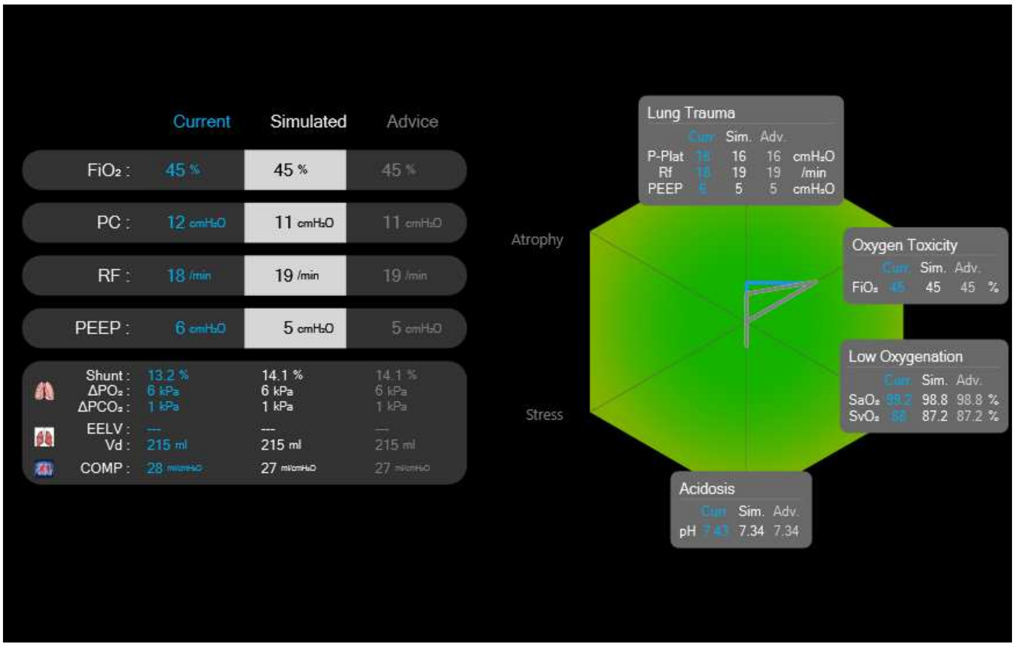

Figure E6 – User interface of the system illustrating an advice including PEEP in a patient ventilated in PC mode.

Figure E7 illustrates the result of following the advice illustrated in figure E8, and the subsequent advice provided by the system. The combined effect of PEEP and PC reduction and increase in Rf was as expected for both oxygenation and respiratory mechanics but less pronounced on pH. The advised level of PEEP was maintained in the next advice, but with an advice to decrease FIO_2 expecting a resulting safe oxygenation. Simulations in the details boxes at the hexagon corners take into account differences between set and measured Rf, where measured Rf was 17 bpm despite set Rf of 19 bpm.

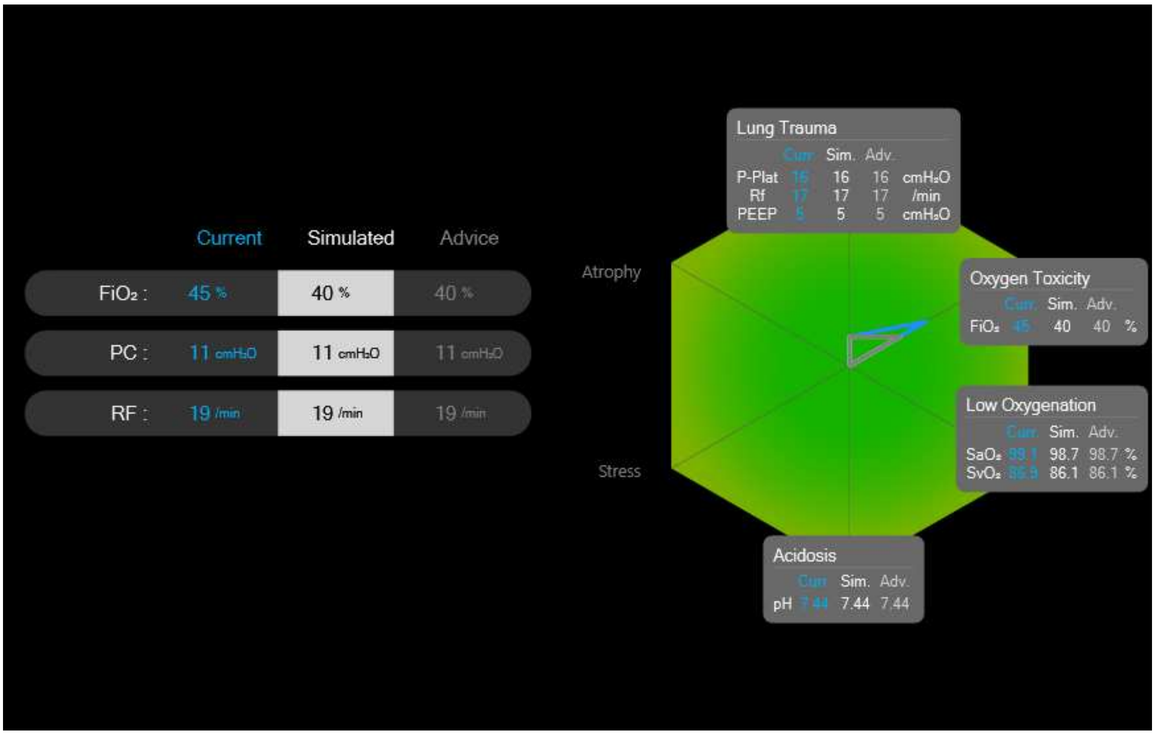

Figure E9 – User interface of the system, with results according to the advice in Figure E6, and the next advice.

SBT setting and review, and extubation check-list

For patients ventilated in pressure support mode with values of PEEP and PS below threshold values illustrated in figure E10, the system provides a counter on the screen which counts down from 30 minutes, and as such indicates that the patient has been within ventilator settings consistent with an SBT and stable with regard other respiratory measurements, for this duration. Cut-off values for these other variables are illustrated in figure E10

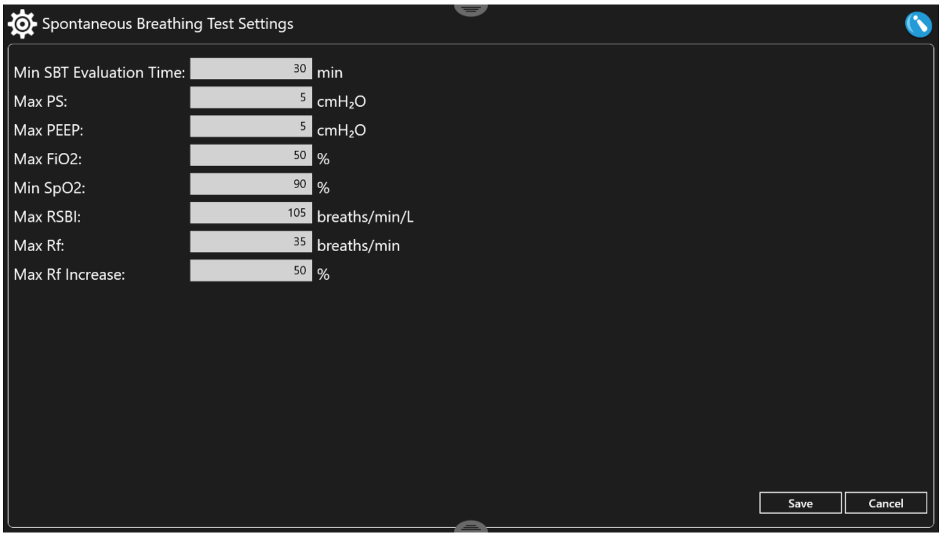

Figure E10 – User interface illustrating variables used to time the SBT counter of the system.

At any point when the counter is running, the user can view the screen illustrated in figure E11. This indicates which variables are within threshold, their values, and for how long this has been the case.

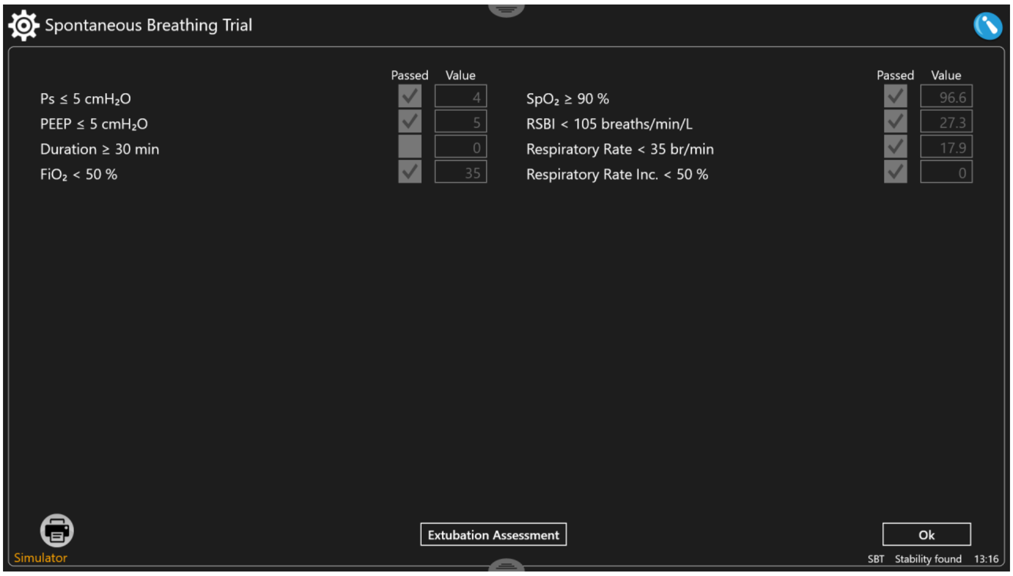

Figure E11 – User interface illustrating status of variables used to track the stability of patients over time in relation to the SBT counter.

When all variables illustrated in figure E11 are within range for the expected duration, the system allows checking of an extubation checklist illustrated in figure E12. Neither the SBT screens E10 and E11, nor the extubation checklist advise on an active SBT test or extubation.

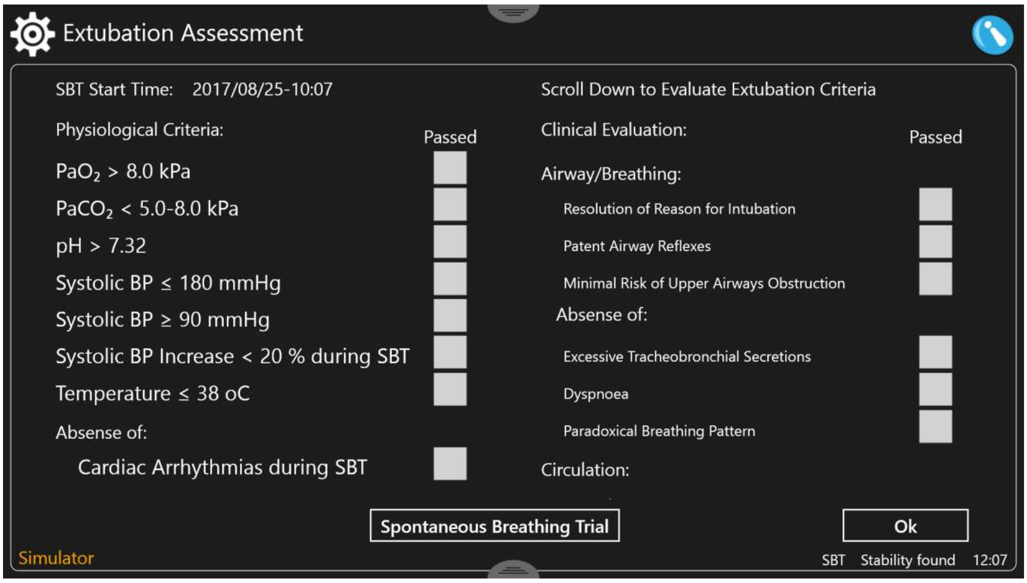

Figure E12a – Extubation checklist, top part of vertical scrolling window.

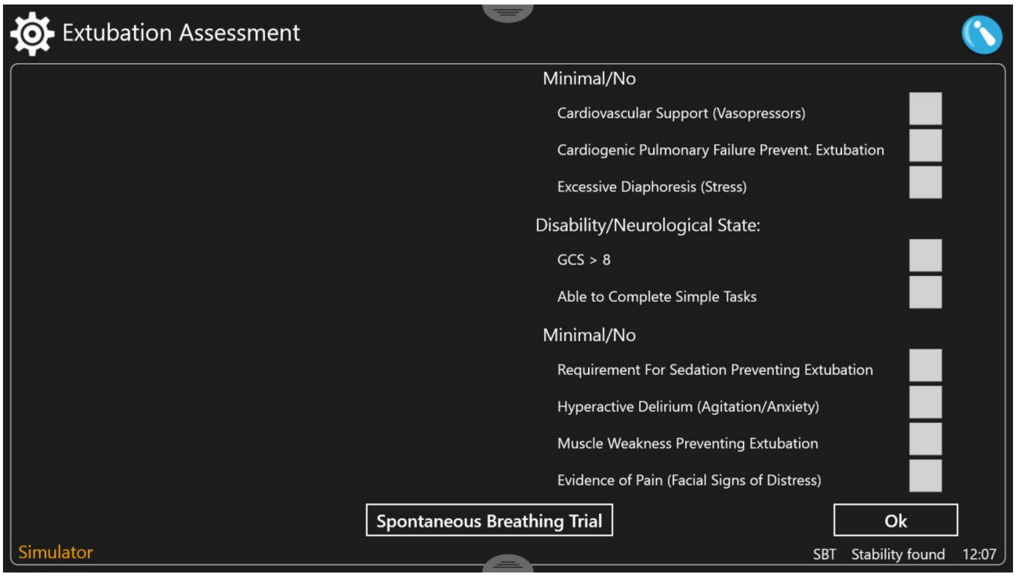

Figure E12b – Extubation checklist, bottom part of vertical scrolling window.

References

1. Karbing DS, Spadaro S, Dey N, Ragazzi R, Marangoni E, Dalla Corte F, Moro F, Lodahl D, Hansen NS, Winding R, Rees SE, Volta CA.. An Open-Loop, Physiologic Model-Based Decision Support System Can Provide Appropriate Ventilator Settings. Crit Care Med. 2018, 46(7):e642-e648

2. Spadaro S, Karbing DS, Dalla Corte F, Mauri T, Moro F, Gioia A, Volta CA, Rees SE. An open-loop, physiological model based decision support system can reduce pressure support while acting to preserve respiratory muscle function. J Crit. Care, 2018 Dec;48:407-413.

3. Rees SE, Karbing DS. Determining the appropriate model complexity for patient-specific advice on mechanical ventilation Biomed Tech (Berl). 201762(2):183-198.

4. Thomsen LP, Karbing DS, Smith BW, Murley D, Weinreich UM, Kjærgaard S, et al. Clinical refinement of the automatic lung parameter estimator (ALPE). J Clin Monit Comput. 2013;27(3):341-50.
