# Supplementary material for: Intensive Care Weaning (iCareWean) protocol on weaning from mechanical ventilation: a single-blinded multicentre randomised control trial comparing an open-loop decision support system and routine care, in the general intensive care unit
Source: BMJ Open. 2020 Sep 2;10(9):e042145. doi: 10.1136/bmjopen-2020-042145 (PMC7470506; doi:10.1136/bmjopen-2020-042145)
Supplement: Author's manuscript [file bmjopen-2020-042145.draft_revisions.pdf]

BMJ Open is committed to open peer review. As part of this commitment we make the peer review history of every article we publish publicly available.

When an article is published we post the peer reviewers' comments and the authors' responses online. We also post the versions of the paper that were used during peer review. These are the versions that the peer review comments apply to.

The versions of the paper that follow are the versions that were submitted during the peer review process. They are not the versions of record or the final published versions. They should not be cited or distributed as the published version of this manuscript.

BMJ Open is an open access journal and the full, final, typeset and author-corrected version of record of the manuscript is available on our site with no access controls, subscription charges or pay-per-view fees (http://bmjopen.bmj.com).

If you have any questions on BMJ Open's open peer review process please email info.bmjopen@bmj.com

BMJ Open

The iCareWean protocol on weaning from mechanical ventilation: A Single-blinded multi-centre randomised control trial comparing an open-loop decision support system and routine care, in the general Intensive Care Unit

Journal:	*BMJ Open*
Manuscript ID	bmjopen-2020-042145
Article Type:	Protocol
Date Submitted by the Author:	26-Jun-2020
Complete List of Authors:	Vizcaychipi, M; Imperial College London, APMIC; Chelsea and Westminster Healthcare NHS Trust, Magill Department of Anaesthesia and Intensive Care Medicine<br>Martins, Laura; Chelsea and Westminster Hospital NHS Foundation Trust, Research Trial Unit<br>White, James; Chelsea and Westminster Hospital NHS Foundation Trust, Magill Department of Anaesthesia<br>Karbing, Dan; Aalborg Universitet, Center for Model-based Medical Decision Support<br>Gupta, Amandeep; West Middlesex University Hospital NHS Trust, Anaesthetic Department<br>Singh, Suveer; Chelsea and Westminster Hospital NHS Foundation Trust, Magill Department of Anaesthesia & Intensive Care medicine<br>Osman, Leyla; Chelsea and Westminster Healthcare NHS Trust, Magill Department of Anaesthetics<br>Moreno-Cuesta, Jeronimo; North Middlesex University Hospital NHS Trust, Anaesthetic Department<br>Rees , Steve ; Aalborg Universitet Institut for Medicin og Sundhedsteknologi
Keywords:	Adult intensive & critical care < ANAESTHETICS, RESPIRATORY MEDICINE (see Thoracic Medicine), Physiology < BASIC SCIENCES

SCHOLARONE™
Manuscripts

I, the Submitting Author has the right to grant and does grant on behalf of all authors of the Work (as defined in the below author licence), an exclusive licence and/or a non-exclusive licence for contributions from authors who are: i) UK Crown employees; ii) where BMJ has agreed a CC-BY licence shall apply, and/or iii) in accordance with the terms applicable for US Federal Government officers or employees acting as part of their official duties; on a worldwide, perpetual, irrevocable, royalty-free basis to BMJ Publishing Group Ltd ("BMJ") its licensees and where the relevant Journal is co-owned by BMJ to the co-owners of the Journal, to publish the Work in this journal and any other BMJ products and to exploit all rights, as set out in our licence.

The Submitting Author accepts and understands that any supply made under these terms is made by BMJ to the Submitting Author unless you are acting as an employee on behalf of your employer or a postgraduate student of an affiliated institution which is paying any applicable article publishing charge ("APC") for Open Access articles. Where the Submitting Author wishes to make the Work available on an Open Access basis (and intends to pay the relevant APC), the terms of reuse of such Open Access shall be governed by a Creative Commons licence – details of these licences and which Creative Commons licence will apply to this Work are set out in our licence referred to above.

Other than as permitted in any relevant BMJ Author's Self Archiving Policies, I confirm this Work has not been accepted for publication elsewhere, is not being considered for publication elsewhere and does not duplicate material already published. I confirm all authors consent to publication of this Work and authorise the granting of this licence.

For peer review only

The iCareWean protocol on weaning from mechanical ventilation: A Single-blinded multi-centre randomised control trial comparing an open-loop decision support system and routine care, in the general Intensive Care Unit.

M. P. Vizcaychipi[1,5*], L. Martins[1] ,J.R. White[1], D.S Karbing[2], , A Gupta[3], S. Singh[1,5], L Osman[1], J. Moreno-Cuesta[4], S.E Rees[2].

1. Magill Department of Anaesthesia, Intensive Care Medicine and Pain Management, Chelsea and Westminster Hospital, C&W NHS Foundation Trust, London, UK.
2. Respiratory and Critical Care group (rcare). Department of Health Science and Technology. Aalborg University, Denmark.
3. Department of Anaesthesia and Intensive Care Medicine, West Middlesex University Hospital, C&W NHS Foundation Trust, London, UK
4. Department of Intensive Care Medicine, North Middlesex University Hospital, London, UK
5. Academic Department of Anaesthesia, Intensive Care & Pain Management, Imperial College London, UK.

**Corresponding Author: Dr Marcela P. Vizcaychipi, email:* m.vizcaychipi@imperial.ac.uk

For peer review only

Abstract

Introduction: Automated systems for ventilator management to date have been either fully heuristic rule-based systems or based on a combination of simple physiological models and rules. These have been shown to reduce the duration of mechanical ventilation in simple to wean patients. At present there are no published studies that evaluate the effect of systems that utilise detailed physiological descriptions of the individual patient.

The BEACON Caresystem© is a model-based decision support system that utilises mathematical models of patients' physiology in combination with models of clinical preferences to provide advice on appropriate ventilator settings. An individual physiological description may be particularly advantageous in selecting the appropriate therapy for a complex, heterogeneous, ICU patient population.

Methods & Analysis: iCareWean is a single-blinded, multicentre, prospective randomised control trial evaluating management of mechanical ventilation as directed by the BEACON Caresystem© compared to that of current care, in the general intensive care setting. The trial will enrol 274 participants across multiple London NHS Intensive Care Units. The trial will utilise a primary outcome of duration of mechanical ventilation until successful extubation.

Ethics & dissemination: Safety oversight will be under the direction of an independent committee of the study sponsor. Study approval was obtained from the regional ethics committee of the HRA, REC reference: 17/LO/0887. IRAS reference: 226610. Results will be disseminated through international critical care conference/symposium and publication in peer-reviewed journal.

Trial Registration details: ClinicalTrials.gov under NCT03249623. This research is registered with the National Institute for Health Research (NIHR) under CPMS ID: 34831.

Keywords: Mechanical ventilation, spontaneous breathing trial, extubation, liberation, weaning, open-loop decision support device, physiological model.

For peer review only

Strengths and limitations of this study

- Single-blinded multi-centre randomised control trial evaluating management of mechanical ventilation as directed by the BEACON Caresystem© compared to that of standard care in the general intensive care setting.
- The BEACON Caresystem© is a model-based decision support system that utilises mathematical models of patients' physiology in combination with models of clinical preferences to provide advice on appropriate ventilator settings.
- Sample size was determined by a power analysis of the duration of mechanical ventilation.
- Patients and the public were not involved in the design of this study.

The study does not assess successful rehabilitation of the patient subsequent to their ICU stay.

For peer review only

Introduction

Optimising mechanical ventilator management has been the focus of much research, seeking to decrease ventilator-associated complications such as ventilator-associated lung injury, ventilator-associated pneumonia, respiratory muscle atrophy, and patient discomfort [1, 2]. Increased duration of mechanical ventilation and prolonged weaning, increases the number of these complications with a vicious cycle that leads to increased morbidity and mortality and associated economic costs [2]. Protocols and computerised tools aiming to reduce length of mechanical ventilation might therefore be beneficial.

Weaning protocols and nurse-lead ventilator management is routinely used in the intensive care setting [2, 3]. Such approaches have been shown to reduce the duration of mechanical ventilation [3]. However, standardising this process is important, and the quality and quantity of nursing care is a critical factor in reducing duration of mechanical ventilation [4].

Standardisation of care through automation or bedside decision support continues to be driven by novel technological innovation, some of which is already established in clinical practice [3, 5]. However, to date much of this has been predicated on clinical guidelines rather than utilising detailed physiological descriptions of the individual patient [6 - 8]. A Cochrane review of weaning trials with current systems concluded that their use may reduce duration of weaning, but highlighted that the population was predominantly composed of those who are "simple to wean" [5]. The patient populations of these studies were characterised as without additional lung pathology and were predominantly ventilated for periods of less than 48 hours [5]. Consequently, it remains to be investigated whether decision support systems can reduce duration of mechanical ventilation in patients with more than a few hours of 'routine' postoperative intubation. The BEACON Caresystem© is a model-based decision support system that utilises mathematical models of patients' physiology in combination with models of clinical preferences to provide advice on appropriate ventilator settings [9]. An individual physiological description may be particularly advantageous in selecting the appropriate therapy for a complex, heterogeneous, ICU patient population. The BEACON Caresystem© is the commercial version of the INVENT system, which has been retrospectively evaluated in post-operative cardiac patients and patients with severe lung disease, and prospectively evaluated in providing advice on correct levels of inspired oxygen [10-13]. To date, the commercial system has been evaluated over short durations of time from 4-8 hours to establish whether physiological measurements are improved [14, 15]. No previous studies evaluating outcome of weaning from mechanical ventilation have been performed.

Here we describe a single-blinded multi-centre randomised control trial evaluating management of mechanical ventilation as directed by the BEACON Caresystem© compared to that of current care in the general intensive care setting.

For peer review only

Methods and Analysis

Administrative Structure

The study is under the auspices of the eHRA, REC reference: 17/LO/0887, IRAS reference: 226610, and is registered at ClinicalTrials.gov under NCT03249623, and with the National Institute for Health Research (NIHR) under CPMS ID: 34831. The Research and Development department of the Chelsea and Westminster NHS Foundation Trust, is sponsor of the study and responsible for study conduct, safety monitoring, personnel training, and randomisation. Data collection occurs at participating centers, with data analysis performed in collaboration with the respiratory and critical care group (rcare), Aalborg University, and independent statisticians of the study sponsor. All data transfer and storage will be performed in accordance with GDPR regulations and the study will monitor and review interim analysis and made recommendations to the steering committee.

Study design

To test the hypothesis that following advice from the BEACON Caresystem© will reduce time to successful extubation, the iCareWean group has designed a single-blinded multi-centre randomised control trial evaluating management of mechanical ventilation as directed by the BEACON Caresystem© compared to that of current care in the general intensive care setting. The Consort flow diagram of the study is illustrated in figure 1.

Description of the BEACON Caresystem©

At the core functionality of the BEACON Caresystem© is a set of physiological models including models of pulmonary gas exchange, acid-base chemistry, lung mechanics, and respiratory drive [9]. The BEACON Caresystem© tunes these models to the individual patient so that they accurately describe the current physiological measurements. Once the models are tuned, they are then used by the system to simulate the effects of a change in ventilator settings. The results of these simulations are then used to calculate the clinical benefit of changing the ventilator settings by balancing the competing goals of mechanical ventilation using mathematical models of clinical preference [9,10]. Appropriate ventilator settings imply a balance between quantified preferences of several competing clinical goals. For oxygenation, the preferred values of inspired oxygen fraction is weighed against the need to prevent hypoxaemia. For settings of ventilation volume, pressures or frequency, the risk of lung trauma is weighed against the need to prevent acidosis. For settings of support in spontaneously breathing patients, the risk of respiratory muscle atrophy on over support is weighed against the excessive work of breathing and potential of respiratory muscle fatigue on under support. Quantification of these risks is used to calculate a total score for any possible ventilator strategy. The system then calculates advice so as to change ventilator settings to improve this score.

The BEACON Caresystem© functions as an "open-loop" system. This means that the advice provided by the system is presented to the clinician. The ventilator settings are then changed by the clinician, and the patient's response to these changes are automatically registered and used by the BEACON Caresystem© to re-tune the physiological models and repeat the process of generating new advice.

For peer review only

The BEACON Caresystem© provides advice on ventilator settings including the following. Values for maximal advice steps are given in table 1.

- Fraction of inspired oxygen (FiO_2)
- Tidal volume (Vt)
- Respiratory frequency (f)
- Positive end expiratory pressure (PEEP)
- Pressure above PEEP

Patient Screening

All patients will be screened for eligibility upon admission to the ICU by the Research team, including research nurses and clinical research fellows. Advice will be obtained from relatives acting as a personal consultee, or Consultant acting as nominated professional consultee. Retrospective consent is then obtained from the patient upon regaining capacity. The study will run from September 2017 to November 2020.

Eligibility Criteria

Inclusion criteria:

1. At least 24 hours of invasive mechanical ventilation.
2. Age ≥ 18 years.
3. Patient is mechanically ventilated in a ventilator mode, and by a ventilator, supported by the BEACON Caresystem©.
4. Haemodynamically stable, where instability is defined by the presence of two or more of the following criteria: acidosis pH < 7.2, poor urine output < 0.5ml/kg/h, use of vasopressors, e.g. noradrenaline > 25 µg/min.

Exclusion criteria:

1. The absence of an arterial catheter for blood sampling.
2. Medical history of home mechanical ventilation which may lead to prolonged stay in the ICU, including long term oxygen therapy and non-invasive ventilation not associated with sleep apnoea.
3. Clinical conditions requiring treatment with ECMO, i.e. an inspired oxygen of 100% for more than 24 hours.
4. Head trauma or other conditions where intra-cranial pressure may be elevated and tight regulation of arterial CO_2 level is paramount.
5. Primary (non-overdose related) neurological patients (Glasgow coma score 24).
6. Severe heart failure classified by grade 4 American Heart Association guidelines.
7. End stage liver disease.
8. Multiple ICU admissions within the medical stratification group, i.e. more than one admission.
9. Corrective orthognathic surgery.
10. ICU admission following oesophageal surgery.
11. Morbidly obese patients defined as either BMI ≥45; or BMI ≥35 with an APACHEII score on admission >24.

For peer review only

12. Pregnancy.
13. Mechanical ventilation initiated for more than 24 hours in other centres.

Randomisation

Figure 1 illustrates the Consort flow diagram for the study. Upon recruitment to the trial, participants are randomised to either to the intervention group where advice is provided by the BEACON Caresystem©, or to the control arm where standard care is provided. Block randomisation is applied with each block stratified to ensure equal distribution between control and intervention arm for the following patient groups, reducing the risk of potential bias by overrepresentation of these groups in the control or intervention arms:

- Surgical or medical ICU admission.
- Presence of absence of chronic obstructive pulmonary disease (COPD).
- Primary thoracic or non-thoracic medical diagnosis.

Study Procedure

In the intervention arm of the trial, the BEACON Caresystem© is activated by the research team and subsequent ventilator management determined by system advice, applied to the ventilator by the clinical team, as illustrated in figure 2 and described below. In the control arm, the BEACON Caresystem© is activated in a mode in which system advice is disabled, but data is still collected by the BEACON Caresystem©. The ventilator management in this arm is determined by standard care.

Intervention arm

Figure 2 illustrates the protocol flow in the intervention arm. At any time during the protocol, adverse events are addressed immediately, with a serious event resulting in exclusion from the study (illustrated as B in figure 2). Minor events are documented and the patient returned to the main protocol flow (illustrated at point A, at the top of figure 2). During intervention, the BEACON Caresystem© can provide a number of different forms of advice to the user which require action. These are illustrated at the top of figure 2, and are described in turn below.

ABG/ALPE - In situations where mathematical model simulations cannot accurately describe continuous measurements, i.e. SpO_2, end-tidal CO_2 ($FetCO_2$), or in support ventilator modes respiratory frequency or tidal volume, then retuning of the models is required. In this situation, the BEACON Caresystem© requests either an arterial blood gas (ABG), or a short procedure varying FiO_2 to assess pulmonary gas exchange (Automatic Lung Parameter Estimator - ALPE), as described and evaluated previously [16-20], and briefly described in the electronic supplementary material.

Ventilator - Advice on changing ventilator settings. New advice is generated only when the system regards current settings as not appropriate. Duration between advice therefore varies, however for PEEP and changes in volume or pressure 10-20 minutes is the minimum period between advice.

Bounds - The BEACON Caresystem© allows for bounds to be set for the limits of advice on ventilator settings. If advice is available which is outside these bounds, the protocol dictates that the bedside nurse consults with the clinician on duty.

For peer review only

Mode/sedation - If the BEACON Caresystem© detects substantial spontaneous breathing during control ventilator modes, advice is provided to consider changing to support mode. Additionally, if pH falls below a defined value in support mode, advice is provided to consider changing to control mode or modifying sedation. In either situation the protocol dictates that the bedside nurse consults with the clinician on duty.

Spontaneous Breathing Trial (SBT) - During weaning from mechanical ventilation the BEACON Caresystem© monitors the levels of PS and PEEP. When these levels are reduced to values below clinical guideline thresholds [21], the BEACON Caresystem© automatically monitors variables evaluating spontaneous breathing criteria. If these remain within limits and for a duration determined by international guidelines [21], the system prompts the user to assess the appropriateness of extubation and provides a checklist to help. The primary function of the BEACON Caresystem© with regards weaning is to reduce levels of support where possible, it does not suggest an active SBT or extubation. As such, the decision to extubate is according to standard clinical practice for both arms of the study, meaning that at any time the clinical team can perform an active SBT or extubate the patient without confirmation from the system. Reintubation is documented by the research team and the participant enters a standard care pathway, which involves the deactivation of the BEACON Caresystem©. The participant is then followed up until the end of the protocol (Figure 1).

The protocol ends with transfer or discharge from ICU or death (Figure 1).

Primary & Secondary Outcomes

The primary outcomes include:

- Duration of mechanical ventilation to successful extubation defined as the time from the start of mechanical ventilation, defined as either the time of intubation in the ICU, or the time of admission to the ICU following previous intubation for surgery, and until successful extubation, with successful extubation defined as ≥48 hours of unassisted spontaneous breathing after extubation.

Unassisted spontaneous breathing is defined here to include supplementary oxygen and support with high flow nasal catheters. Patients with non-invasive mask ventilation are considered to be assisted.

The secondary objectives of the trial are to compare the following variables in the standard care group and BEACON Caresystem© group:

- Duration of mechanical ventilation to successful extubation following randomisation defined as the time from randomisation and until successful extubation, with successful extubation defined as ≥48 hours of unassisted spontaneous breathing after extubation.
- Time from control mode to support mode defined as the time following randomisation, from initiation of control modes of ventilation and until initiation of support modes of ventilation.
- Time from support mode to successful extubation defined as the time following randomisation, from initiation of support modes of ventilation and until successful extubation.

For peer review only - http://bmjopen.bmj.com/site/about/guidelines.xhtml

- Number of changes in ventilator settings per day defined as the daily registered number of changes to ventilator settings from randomisation until successful extubation.
- Time to first SBT defined as the time from randomisation to the first performed SBT.
- Time to first successful SBT defined as the time from randomisation to the first successful SBT.
- Time to first extubation defined as the time from randomisation to the first extubation attempt.
- Time to first disconnection from mechanical ventilation defined as the time from randomisation to the point at which mechanical ventilation is first discontinued.
- Percentage of time in control mode ventilation defined as the time from randomisation which is spent in controlled modes of mechanical ventilation in percent of duration of mechanical ventilation.
- Percentage of time in support mode ventilation defined as the time from randomisation which is spent in support modes of mechanical ventilation in percent of duration of mechanical ventilation.
- Use of sedatives defined as the cumulative use of sedative drugs from randomisation until successful extubation.
- Use of neuromuscular blockading agents defined as the cumulative use of neuromuscular blockading agents from randomisation until successful extubation.
- Number of intubation free days defined as the number of days without intubation from randomisation until successful extubation.
- Number of reintubations defined as the number of reintubations following extubation from randomisation until successful extubation.
- Number of tracheostomies defined as the number of patients having tracheostomy performed from randomisation until successful extubation or protocol end.
- Number of patients on prolonged mechanical ventilation defined as the number of patients on mechanical ventilation ongoing for more than 21 days after the start of mechanical ventilation.
- Number and types of adverse events related to mechanical ventilation defined as the incidence of adverse events directly related to mechanical ventilation.
- ICU mortality defined as the mortality from randomisation and until death or ICU discharge.
- Length of stay defined as the duration of ICU admission from the start of mechanical ventilation and until death or ICU discharge.
- Mobilization times defined as the time from randomisation until a) first mobilization, e.g. sitting on the edge of the bed, standing up and marching on the spot; and b) regaining independency, e.g. able to drink/eat or comb hair.
- Daily patient physiological blood gas status defined as daily arterial blood gas values of SaO_2, PaO_2, $PaCO_2$, pH from randomisation until successful extubation.
- Daily average physiological status defined as daily averages of measured values of oxygenation (SpO_2), end-tidal CO_2 fraction ($EtCO_2$), ventilation (respiratory rate, tidal volume, anatomical dead space), pulmonary mechanics (Airway pressure, respiratory system compliance), and ventilator settings as measured routinely at the bedside.

Statistical considerations

Sample size was determined by a power analysis of duration of mechanical ventilation. Extrapolation of the 2016 ICU admission data from two London based NHS hospitals provided a sample size of 548 patients over an 18 month period. In these patients, mean total duration of mechanical ventilation

For peer review only

measured in days was 7.2, with a standard deviation of 7.0 days. A study population of 137 patients in each randomisation group, would allow detection of a 2 day or 30% improvement in the duration of mechanical ventilation in the BEACON Caresystem© group assuming a similar improvement in SD, with an alpha level of 0.05 and power of 0.8. A 2 day or 30% reduction in duration of mechanical ventilation is similar to that used previously [22].

Primary and secondary outcomes will be measured across the protocol duration and comparison made by independent samples *t*-test, or appropriate non-parametric testing if not normally distributed. Complementary analysis will be performed using Kaplan-Meier curves.

The study is not sufficiently powered to allow subgroup analysis for the groups specified in the randomisation strategy

An independent data safety monitoring committee will be established as part of the study, with this committee responsible for defining stopping criteria. Safety data will be looked at after the first 80 patients have been recruited, with an interim analysis performed following 50% recruitment.

Safety, Ethics and Dissemination

Adverse events

All adverse events will be monitored and documented until the end of study. Adverse events are reported to the trial Sponsor according to their standard operating procedure for further investigation. As patients are critically ill it is expected that unrelated adverse events will occur as part of hospital stay. Adverse events are therefore only defined in relation to changes in mechanical ventilation, for patients allocated to either the control or intervention arms.

1. Serious adverse events are defined as:
   - Death, suspected to be caused directly by the settings of mechanical ventilation.

2. Adverse events include the following events suspected to be caused directly by the settings of mechanical ventilation:
   - Pneumothorax
   - Self-extubation
   - Hypoxaemia (SpO_2 <90%; PaO_2 <8.00 kPa)
   - Alkalaemia (pH >7.5)
   - Acidaemia (pH <7.2)

Safety Considerations, systems advice

The potential risks and safety considerations related to the use of the The BEACON Caresystem© have been considered and include:

1. Inappropriate suggestions for changes in ventilator settings

Inappropriate changes in ventilator settings, if applied, can have serious consequences. The general strategy employed in this protocol has three components:

i. The BEACON Caresystem© only provides advice in the situation where the models can accurately describe all psychological measurements. The ability of BEACON Caresystem© to do so is automatically assessed by the system throughout the protocol and failure to do so means that no advice is provided, which is made visible to the user.

For peer review only

ii. Maximal limits have been applied to the amount for which each ventilator setting can be changed in a single advice, meaning that inappropriate advice should have a reduced impact. The maximal advice changes for each ventilator setting are given in Table 1.

iii. The risks of changing each of the ventilator settings have been considered individually. Low values of inspiratory pressure or volume can result in reduction of minute ventilation and increase in $PaCO_2$ and consequently a fall in arterial pH. To assess this, CO_2 is monitored continuously by the BEACON Caresystem© using capnography. High values of inspiratory pressure can result in lung damage. Peak inspiratory pressure (PIP) is monitored continuously by the BEACON Caresystem©, and all mechanical ventilators provide alarms at high values. Low values of respiratory frequency have no apparent detriment other than those specified in reduction of volume. High values of respiratory frequency can result in air trapping. The maximum step in respiratory frequency is limited to 3 breaths/min if the current frequency is ≥ 18 allowing the onset of trapping to be identified clinically.

iv. Although there are small long term risks associated with increasing inspiratory oxygen due to absorption atelectasis or oxygen toxicity, the major acute risk of changes in inspired oxygenation is arterial hypoxaemia. If the current SpO_2 ≤92%, then the maximum reduction in FiO_2 is limited to 2%. In addition, pulse oximetry is monitored continuously by the BEACON Caresystem© during the study and an alarm sounded if levels are ≤90%.

To encourage use of the system and perform an evaluation where advice is applied, the application of advice is mandatory. As clinical practice is variable and advice is unlikely to be applied in all cases, the application and effects of advice are monitored by the Beacon Caresystem, and can be interrogated to ensure that proposed changes have been made and that the effects are considered safe in cases of doubt.

2. System malfunction

In the event of system malfunction the patient enters standard care until the malfunction is detected and corrected by biomedical engineers.

3. Sampling and measurement of arterial blood gas values

Arterial blood gas sampling is part of routine ICU care. Additional arterial blood gas sampling may be required by the BEACON Caresystem© than those performed in clinical practice. The BEACON Caresystem© requirements, estimated from current experience, are expected to be a maximum of 6 per day in the intervention group.

All other measurements used are obtained from non-invasive means including pulse oximetry and measurement of respiratory gas content, flow and pressure.

Ethical approval and Consent

Study approval was obtained from the regional ethics committee of the HRA, REC reference: 17/LO/0887. IRAS reference: 226610. The majority of the trial population lack the capacity to consent. Therefore, participant information sheets (PIS) and advice is requested from a third party acting as a consultee; in most cases this person will be a Personal Consultee, who is someone who knows the person lacking capacity and is able to advise the researcher about that person's wishes and feelings in relation to the project and whether they should join the research. However, where the personal consultee is not available, the researcher may nominate a professional person to assist

For peer review only

in determining the participation of a person who lacks capacity. A Nominated Professional Consultee will be appointed by the researcher to provide advice regarding the patient's suitability to participation in the research. In the event that a personal consultee is identified after the nominated professional consultee advice has been obtained, the above process for personal consultee declaration will be followed. A patient information sheet will be distributed immediately following the patient being identified as eligible for the study. If a surviving patient regains capacity, they will be approached by the research team to obtain their consent to continue in the study. If the patient refuses consent or withdraws the intervention will be stopped but the regular/expected medical care will still be provided. Without consent, no additional information about the patient will be collected for the purposes of the study. However, to maintain integrity of the randomised trial, all information collected up to that time will still be used and analysed as part of the study.

Dissemination Plan

The dissemination strategy will involve the presentation of the results at international critical care conferences and publication in a peer-reviewed journal.

Patient and public involvement

Patients and the public were not involved in the design of this study. Patients randomised into the study will be informed of the results of the study once the outcome of the study is published.

Discussion

This paper presents the study protocol of single-blinded multi-centre randomised control trial evaluating management of mechanical ventilation as directed by the BEACON Caresystem© compared to that of standard care in the general intensive care setting. To test the hypothesis that following advice from the BEACON Caresystem© will reduce time to successful extubation, patients are randomised to care including advice from the system, or standard care. In addition, as secondary outcomes, the study will investigate whether application of the advice changes the time spent in control ventilation modes, the number of changes made to the ventilator, the use of medication such as sedative or neuromuscular blocking agents, the changes in clinical procedures such as re-intubation or tracheostomy, and clinical and physiological status of the patient. Although not powered to detect differences between groups, it will also investigate the length of stay at the ICU and patient mortality.

This study is the next step in evaluation of the advice of the BEACON Caresystem©. A large number of studies have been performed validating the physiological models included in the system, as summarized in [9], and studies of short duration have been performed evaluating the physiological response to application of advice [14,15]. This use of the system over the whole duration of ICU stay allows for assessment of more meaningful clinical outcomes, relating to the time spent on mechanical ventilation. In addition, the study includes only patients intubated for a minimum of 24 hours, meaning that short term post-operative patients who may have rather uncomplicated intubation periods, are not included.

The study includes limitations. The study was powered on duration of mechanical ventilation rather than duration to successful ventilation, this being the historical data available during study design.

For peer review only

The study is not powered to detect changes in mortality, and does not follow up on the patients after transfer from the ICU. In the context of this study, successful extubation is defined as the lack of need for non-invasive ventilator support for 48 hours after extubation, rather than successful rehabilitation of the patient subsequent to their ICU stay.

The results of this study compare care provided with the system's advice with current clinical practice at study sites. As there is no standard of care or usual method of weaning in the United Kingdom, this study does not compare the system's advice with that of best practice defined by clinical guidelines. To avoid comparing two interventional changes, no attempt will be made to standardize care in the control arm according to best practice.

As patients are studied for many days it is not possible to store continuous waveform data for all patients at all times. Systematic analysis of patient-ventilator asynchrony will not therefore be possible.

This study excluded patients if ventilated for <24 hours. In doing so, patients ventilated post-surgery defined previously as "simple to wean" are not included. This should not imply that the patients studied here are necessarily difficult to wean, but rather that they represent a broad cross-section of mechanically ventilated patients not simply recovering from the effects of anaesthesia.

In addition, it is important to note that substantial training has been provided to all sites in the use of the BEACON Caresystem© and the same clinical team treat patients for both the control and intervention arm. It is possible then that both training and use of the system influence routine care, potentially biasing study results.

Conclusion

This paper describes the study protocol of a randomised control trial evaluating the advice of the BEACON Caresystem© in a general medical ICU population. This protocol represents an important step evaluating the use of advice over the whole of the ICU stay, and only in patients intubated greater than 24 hours. The results will indicate whether the BEACON Caresystem© is a useful tool in optimising ventilation strategy.

Acknowledgements:

The authors would like to thank Dr Sundhiya Mandelia, Senior statistician for reviewing the method section of this protocol. Mr Damon Foster and Dr Essam Ramhamadany for their support and contributions to the setup of the clinical study. The critical care nurses and physicians working in the NHS who feedback on the protocol.

For peer review only

References:

1. The ARDS Network. Ventilation with Lower tidal volumes as compared with traditional tidal volumes for acute lung injury and acute respiratory distress syndrome. *NEJM* 2004; 342: 1301-1308.
2. Pham T, Brochard LJ, Slutsky AS. Mechanical Ventilation: State of the Art. *Mayo Clin Proc* 2017; 92(9):1382-1400.
3. Blackwood B, Alderdice F, Burns K, Cardwell C, Lavery G, O'Halloran P. Use of weaning protocols for reducing duration of mechanical ventilation in critically ill adult patients: Cochrane systematic review and meta-analysis. *BMJ* 2011; 342:c7238.
4. Danckers M, Grosu H, Jean R, Cruz RB, Fidellaga A, Han Q, Awerbuch E, Jadhav N, Rose K, Khouli H. Nurse-driven, protocol-directed weaning from mechanical ventilation improves clinical outcomes and is well accepted by intensive care physicians. *J Crit Care* 2013;28:433-41.
5. Rose L, Schultz MJ, Cardwell CR, Jouvet P, McAuley DF, Blackwood B. Automated versus non-automated weaning for reducing the duration of mechanical ventilation for critically ill adults and children. *Cochrane Database of Systematic Reviews* 2014; 6: CD009235.
6. Lellouche F, Mancebo J, Jolliet P, Roeseler J, Schortgen F, Dojat M, Cabello B, Bouadma L, Rodriguez P, Maggiore S, Reynaert M, Mersmann S, Brochard L. A multicenter randomized trial of computer-driven protocolized weaning from mechanical ventilation. *Am J Respir Crit Care Med* 2006; 15;174(8):894-900.
7. Sulemanji D, Marchese A, Garbarini P, Wysocki M, Kacmarek RM. Adaptive Support Ventilation: An Appropriate Mechanical Ventilation Strategy for Acute Respiratory Distress Syndrome? *Anesthesiology* 2009; 111(4):863-70.
8. Schädler, D, Miestinger, G, Becher, T, Frerichs, I, Weiler, N, Hörmann, C. Automated control of mechanical ventilation during general anaesthesia: study protocol of bicentric observational study (AVAS). *BMJ Open* 2017; 7e014742.
9. Rees SE, Karbing DS. Determining the appropriate model complexity for patient-specific advice on mechanical ventilation. *Biomed Tech (Berl)* 2017; 62(2):183-198.
10. Rees SE, Allerød C, Murley D, Zhao Y, Smith BW, Kjærgaard S, Thorgaard P, Andreassen S. Using physiological models and decision theory for selecting appropriate ventilator settings. Journal of Clinical Monitoring and Computing, 2006; Dec;20(6):421-429
11. Karbing DS, Allerød C, Thomsen LP, Espersen K, Thorgaard P, Andreassen S, Kjærgaard S, Rees SE. Retrospective evaluation of a decision support system for controlled mechanical ventilation. Med Biol Eng Comput. 2012 Jan;50(1):43-51.
12. Karbing DS, Allerød C, Thorgaard P, CariusAM, FrilevL, Andreassen S, Kjærgaard S, Rees SE. Prospective evaluation of a decision support system for setting inspired oxygen in intensive care patients. Journal of Critical Care, 2010, 25(3):367-74.
13. C Allerød, S.E Rees, B.S Rasmussen, D.S Karbing, S Kjærgaard, P Thorgaard, S Andreassen. A Decision Support System for suggesting ventilator settings: Retrospective evaluation in cardiac surgery patients ventilated in the ICU. Computer Methods and Programs in Biomedicine, 2008, vol. 92, nr. 2, s. 205-212
14. Karbing DS, Spadaro S, Dey N, Ragazzi R, Marangoni E, Dalla Corte F, Moro F, Lodahl D, Hansen NS, Winding R, Rees SE, Volta CA.. An Open-Loop, Physiologic Model-Based Decision Support System Can Provide Appropriate Ventilator Settings. Crit Care Med. 2018, 46(7):e642-e648

For peer review only - http://bmjopen.bmj.com/site/about/guidelines.xhtml

15. Spadaro S, Karbing DS, Dalla Corte F, Mauri T, Moro F, Gioia A, Volta CA, Rees SE. An open-loop, physiological model based decision support system can reduce pressure support while acting to preserve respiratory muscle function. J Crit. Care, 2018 Dec; 48:407-413.
16. S.E Rees, S Kjærgaard, P Thorgaard, J Malczynski, E Toft, S Andreassen. The Automatic Lung Parameter Estimator (ALPE) system: Non-invasive estimation of pulmonary gas exchange parameters in 10-15 minutes. Journal of Clinical Monitoring and Computing, 2002, Vol 17, No.1, pp 43-52.
17. Karbing DS, Kjærgaard S, Andreassen S, Espersen K, Rees SE. Minimal model quantification of pulmonary gas exchange in intensive care patients. Med Eng Phys. 2011 Mar;33(2):240-8.
18. S.E Rees, S Kjærgaard, S Andreassen, G. Hedenstierna. Reproduction of MIGET retention and excretion data using a simple model of gas exchange in lung damage caused by oleic acid infusion. Journal of Applied Physiology, 2006 Sep;101(3):826-32.
19. S.E Rees, S Kjærgaard, S Andreassen, G. Hedenstierna. Reproduction of inert gas and oxygenation data – a comparison of the MIGET and a simple model of pulmonary gas exchange. Intensive Care Medicine 2010;36:2117-2124.
20. D.S Karbing, S Kjaergaard, B.W Smith, K Espersen, C Allerod, S Andreassen, S.E Rees. Variation in the PaO2/FiO2 ratio with FiO2: Mathematical and experimental description, and clinical relevance. Critical Care. 2007; Vol. 11, No. 6.
21. Boles JM, Bion J, Connors A, Herridge M, Marsh B, Melot C, Peral R, Silverman H, Stanchina M, Vieillard-Baron A, welte T. Weaning from mechanical ventilation. Eur Respir J. 2007; 29:1033-1056.
22. Lellouche F, Mancebo J, Jolliet P, Roeseler J, Schortgen F, Dojat M, Cabello B, Bouadma L, Rodriguez P, Maggiore S, Reynaert M, Mersmann S, Brochard L. A multicenter randomized trial of computer-driven protocolized weaning from mechanical ventilation. Am J Respir Crit Care Med. 2006 Oct 15;174(8):894-900.

Authors' Contributions: MPV, DSK and SER conceived, designed and wrote the protocol. MPV drafted, reviewed and amended the manuscript. LM, edited figures, drafted R&D documents. JRW drafted Standard operating procedure, created original figures and drafted the initial protocol manuscript. SS, AG, LO, LM, JMC, SER and MPV reviewed and approved the final draft of the manuscript.

Funding Statement: This work was supported by Mermaid Care A/S and the National Institute of Health Research (NIHR).

Competing Interests Statement: SER and DSK are minor shareholders of Mermaid Care A/S who manufacture the Beacon Caresystem©. They have also acted as consultants for Mermaid Care A/S. SER is an unpaid board member of Mermaid Care A/S. Rest of the authors do not have competing interests to declare.

For peer review only

Tables and Figures legends/captions

Table 1. Maximal steps in mechanical ventilation advice provided by BEACON Caresystem©

Figure 1. Consort flow diagram for protocol.

Figure 2. Flowchart of BEACON Caresystem© intervention arm. Arterial Blood Gas (ABG)/ Automatic Lung Parameter Estimator (ALPE), Spontaneous Breathing Trial (SBT), Clinical Research Form (CRF).

For peer review only

Maximal allowed step in ventilator settings	Stepping up	Stepping down
FIO_2	10 %	5% or 2% when $SpO_2 \leq$ 92 %
Vt (volume modes)	50 ml	50 ml
Respiratory frequency (f)	5 breaths/min or 3 breaths/min when f ≥ 18	5 breaths/min
Pressure above PEEP (pressure modes)	2 cm H_2O	2 cm H_2O
PEEP	3 cm H_20 if PEEP < 10 cm H_20, otherwise 2 cm H_20	2 cm H_2O

Maximal steps in mechanical ventilation advice provided by BEACON Caresystem© .

254x190mm (96 x 96 DPI)

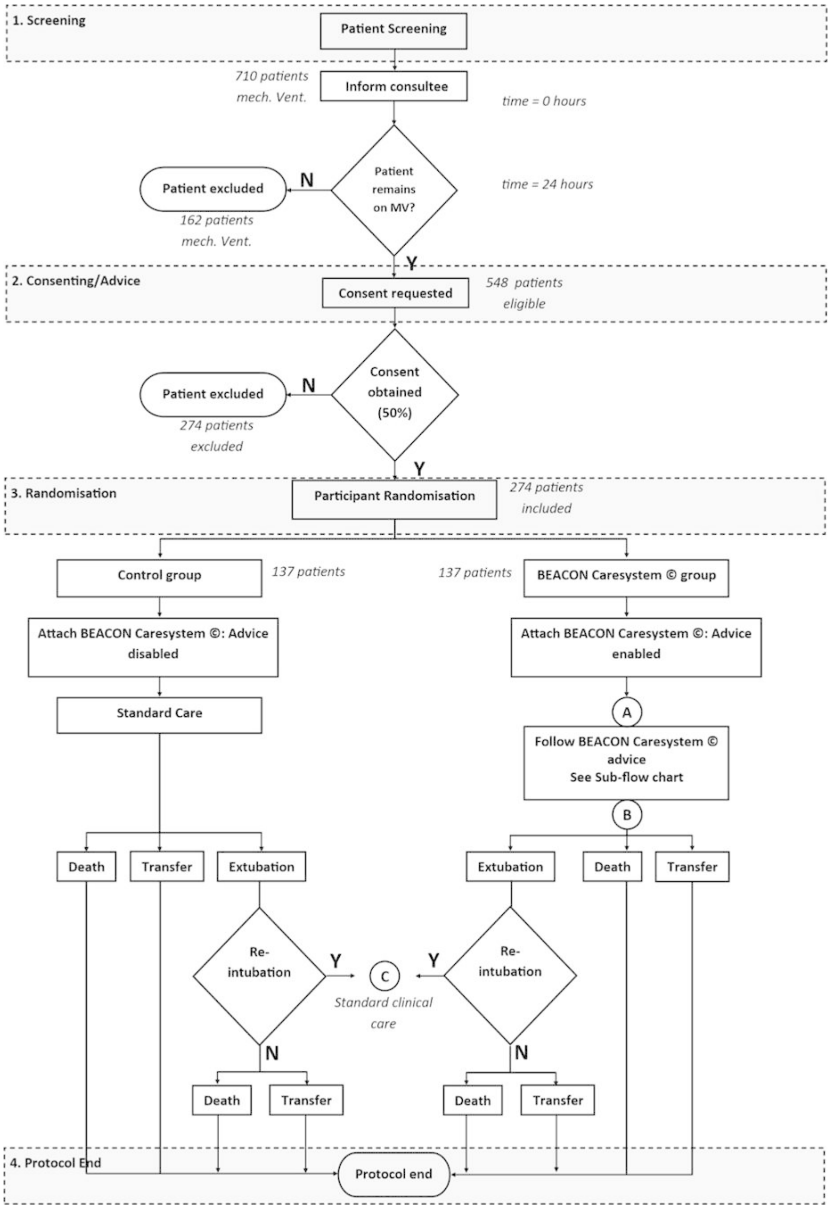

Consort flow diagram for protocol.

156x227mm (150 x 150 DPI)

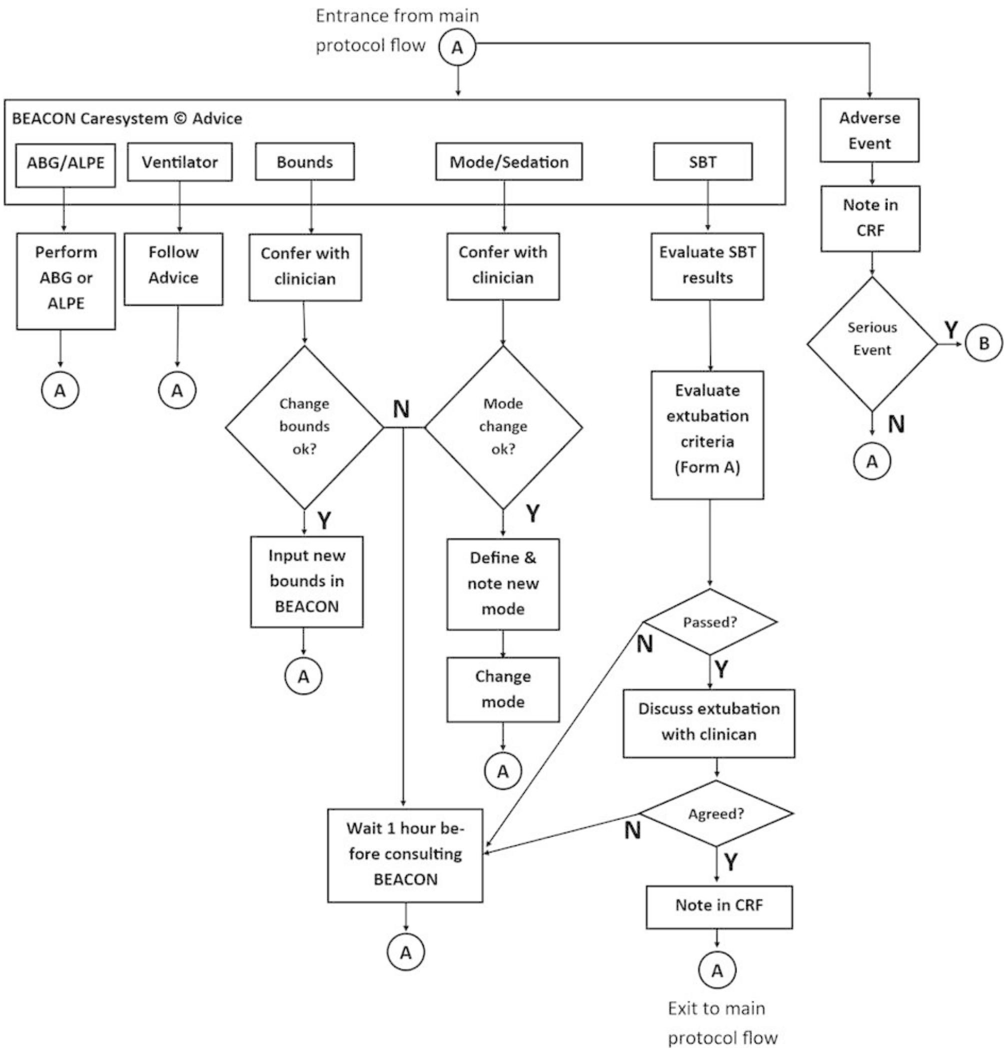

Flowchart of BEACON Caresystem© intervention arm. Arterial Blood Gas (ABG)/ Automatic Lung Parameter Estimator (ALPE), Spontaneous Breathing Trial (SBT), Clinical Research Form (CRF).

159x165mm (150 x 150 DPI)

SUPPLEMENTARY MATERIAL

The following is supplementary material for:

The iCareWean protocol on weaning from mechanical ventilation: A Single-blinded multi-centre randomised control trial comparing an open-loop decision support system and routine care, in the general Intensive Care Unit.

M. P. Vizcaychipi[1,5*],L. Martins[1], J.R. White[1], D.S Karbing[2], , A Gupta[3], S. Singh[1,5], L Osman[1], J. Moreno-Cuesta[4], S.E Rees[2].

1. Magill Department of Anaesthesia, Intensive Care Medicine and Pain Management, Chelsea and Westminster Hospital, C&W NHS Foundation Trust, London, UK.
2. Respiratory and Critical Care group (rcare). Department of Health Science and Technology. Aalborg University, Denmark.
3. Department of Anaesthesia and Intensive Care Medicine, West Middlesex University Hospital, C&W NHS Foundation Trust, London, UK
4. Department of Intensive Care Medicine, North Middlesex University Hospital, London, UK
5. Academic Department of Anaesthesia, Intensive Care & Pain Management, Imperial College London, UK.

**Corresponding Author: Dr Marcela P. Vizcaychipi, email: m.vizcaychipi@imperial.ac.uk*

For peer review only - http://bmjopen.bmj.com/site/about/guidelines.xhtml

The purpose of this supplementary material is to provide the reader with an understanding of the structure and function of the Beacon Caresystem©, described here as the clinical decision support system (CDSS). The material includes four sections describing: the mathematical models included in the system, including description of the models used to describe the effects of PEEP; the use of the system; a section illustrating the presentation of advice to the clinician; and a section illustrating screens relating to SBT and the extubation checklist. Much of the material presented here, including patient examples is taken from supplementary material from previous publications (1,2).

Mathematical models included in the CDSS

Figure E1 illustrates the structure of the mathematical models with a full description of model formulation and evaluation published recently (1,2,3). The system includes mathematical models of pulmonary gas exchange; respiratory mechanics; acid-base chemistry of blood, interstitial fluid, tissues and cerebral spinal fluid; respiratory drive and ventilation. In addition, the models include the effects of PEEP on gas exchange, pulmonary mechanics and ventilation as illustrated in figure E2 and described below. All models are tuned to the individual patient's physiological status through measurement of respiratory gas flows and pressures; calorimetry and capnography measurements of respiratory gas fractions of O_2 and CO_2, and subsequent calculation of oxygen utilisation (VO_2) and carbon dioxide production (VCO_2); pulse oximetry measurement of arterial oxygen saturation; and arterial blood measurements of acid-base, oxygenation and haemoglobin fractions. The model of pulmonary gas exchange is tuned to the appropriate matching of ventilation and perfusion to account for O_2 and CO_2 differences between arterial and end tidal gas values. To do so an arterial blood gas (ABG) is required on system start up. In some patients, the system also requires modification of FIO_2 to two levels for 2-5 minutes at each level to tune the pulmonary gas exchange model to the patient, a procedure previously called the automatic lung parameter estimator (ALPE) technique(3,4).

For peer review only - http://bmjopen.bmj.com/site/about/guidelines.xhtml

The respiratory mechanics model is tuned to dynamic compliance. The model of acid-base chemistry of the blood is tuned to measured values of arterial pH, PCO_2, PO_2, and SO_2, and haemoglobin concentration, with the acid-base chemistry of the cerebrospinal fluid (CSF) regulated to arterial bicarbonate values to account for chemical changes in respiratory drive. The respiratory drive model is tuned to describe the relationship between alveolar ventilation (VA) and arterial acid-base and oxygenation status. The ventilation model is tuned to the measured serial dead space (V*ds*) to allow calculation of alveolar ventilation. A series of algorithms are present in the CDSS to re-tune the models as new measurements present, or if the patient condition changes. These models are used to simulate the effect of changes in ventilator settings, with the system generating advice based upon simulated values.

For peer review only

For peer review only - http://bmjopen.bmj.com/site/about/guidelines.xhtml

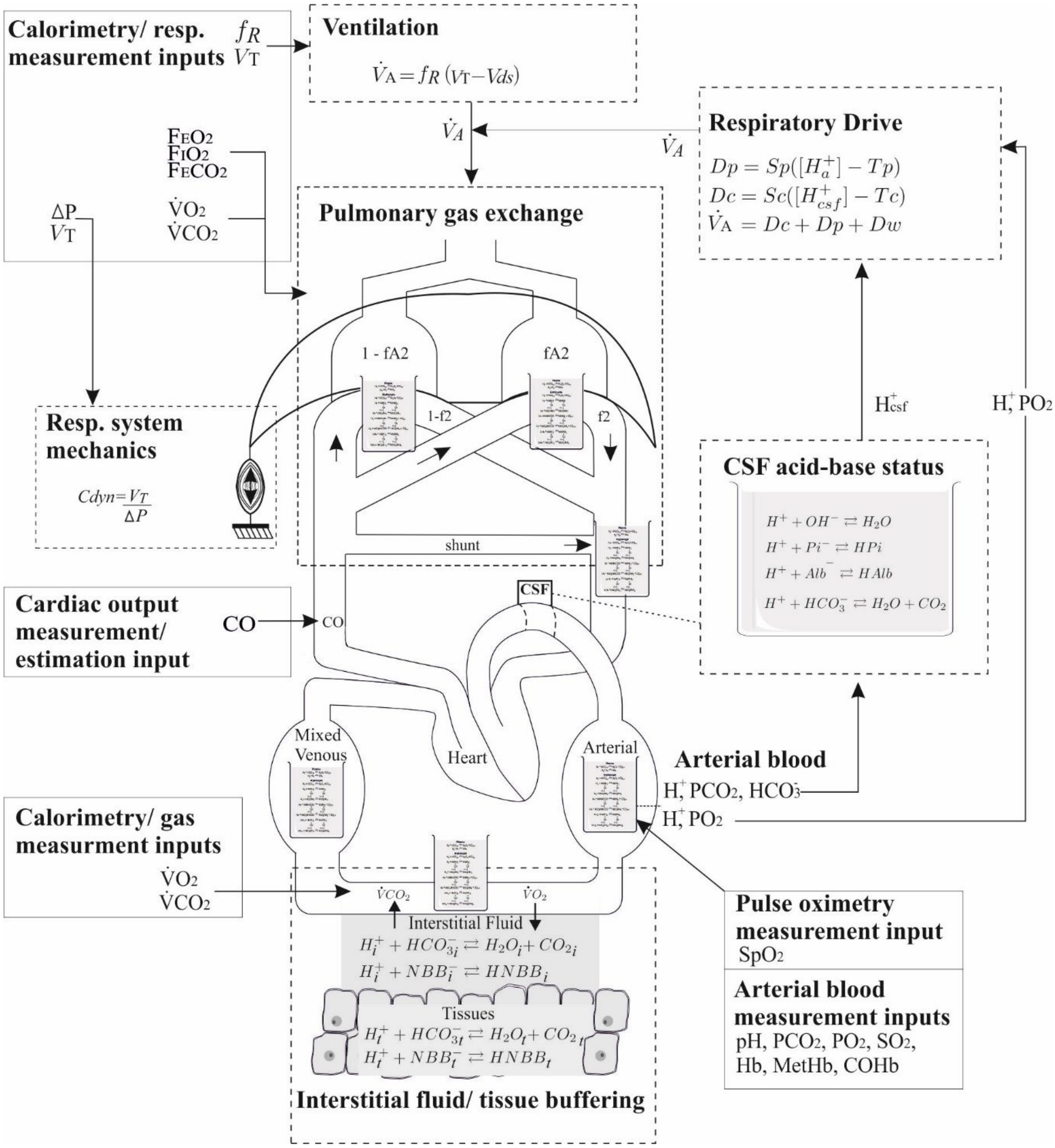

Figure E1: Mathematical models included in the CDSS, modified from (3). Mathematical models are indicated by dashed boxes with the exception of the acid-base model of blood, which is illustrated in each blood compartment as used. Measured input values are indicated by solid boxes.

For peer review only - http://bmjopen.bmj.com/site/about/guidelines.xhtml

Figure E2 illustrates the mathematical models built to describe the effects of PEEP. These are formulated as empirical linear models, with the system only advising on PEEP changes of a maximum of 3 cmH_2O in any step and the slope of these relationships modified automatically on measuring the response to PEEP. These models are integrated with those in figure E1 to enable simulation of the patient specific effects of PEEP and other ventilator settings simultaneously, enabling the system to advise on changing settings toward the best compromise for the individual patient given the model simulations.

For patients with acute respiratory failure, PEEP is usually set to improve gas exchange and lung mechanics, and the sub-figures A, B and C of figure E2 illustrate the baseline expected response to changes in PEEP of pulmonary shunt, respiratory system compliance and high ventilation/perfusion (V/Q). Changes in shunt, compliance and high V/Q automatically result in model-simulated changes in oxygenation, ventilation volumes and pressures, and carbon dioxide partial pressures through the models illustrated in figure E1. For shunt and compliance, the initial slopes of the models are dependent on the initial levels of shunt and compliance, with a greater improvement expected for a greater severity of respiratory abnormality. The slopes of these models are adapted automatically according to patient response to changes in PEEP, from measurements of oxygenation (SpO_2), respiratory volumes and pressures, and end tidal CO_2 values. Any increase in end tidal CO_2 values that are simulated to result in severe acidosis result in the system requesting an arterial blood gas. The decision to increase PEEP is then based on the potential benefit of improved oxygenation and respiratory volumes and pressures, given the tuning of the models to the patient's state. For patients responding poorly to increases in PEEP, the absolute slope of shunt and compliance models would be substantially reduced following measurement of the response to PEEP changes. The decision to reduce PEEP at high SpO_2 levels depends upon the FIO_2 level. FIO_2 will always be optimized first, such that the competing goals of sufficient oxygenation and oxygen toxicity are balanced. Following this, the system will calculate the likely effects on oxygenation and respiratory volumes and pressures on reducing PEEP from the models illustrated on subfigures A, B and C of figure E2, combined with the models of E1. For patients recovering from respiratory injury, without substantial pulmonary shunt

For peer review only - http://bmjopen.bmj.com/site/about/guidelines.xhtml

or low respiratory system compliance, the system will calculate that the potential negative effects of reducing PEEP will be outweighed by the positive effects of reducing plateau pressure.

For patients on pressure support ventilation without substantial problems related to gas exchange or respiratory system mechanics, PEEP is often used to support the respiratory muscles, preventing abnormal respiratory muscle activity as indicated by abnormal breathing patterns. Figure E2, D and E represent the empirical linear models included in the system to account for changes in tidal volume (VT) due to PEEP. Figure E2D illustrates two potential abnormal situations; the first is a very high VT at a low PEEP value, and the second a very low VT at a high PEEP, possibly representing the situation where the diaphragm may be over distended due to high PEEP. In these situations, the initial slope of the PEEP versus VT relationship is negative. Figure E2E illustrates the situation of a low tidal volume at a low value of PEEP, possible due to under-support of the respiratory muscles. In this situation, the initial slope of the PEEP versus VT relationship is positive. As with other PEEP models, all slopes are adapted following measurement of the response to PEEP changes.

For peer review only

For peer review only - http://bmjopen.bmj.com/site/about/guidelines.xhtml

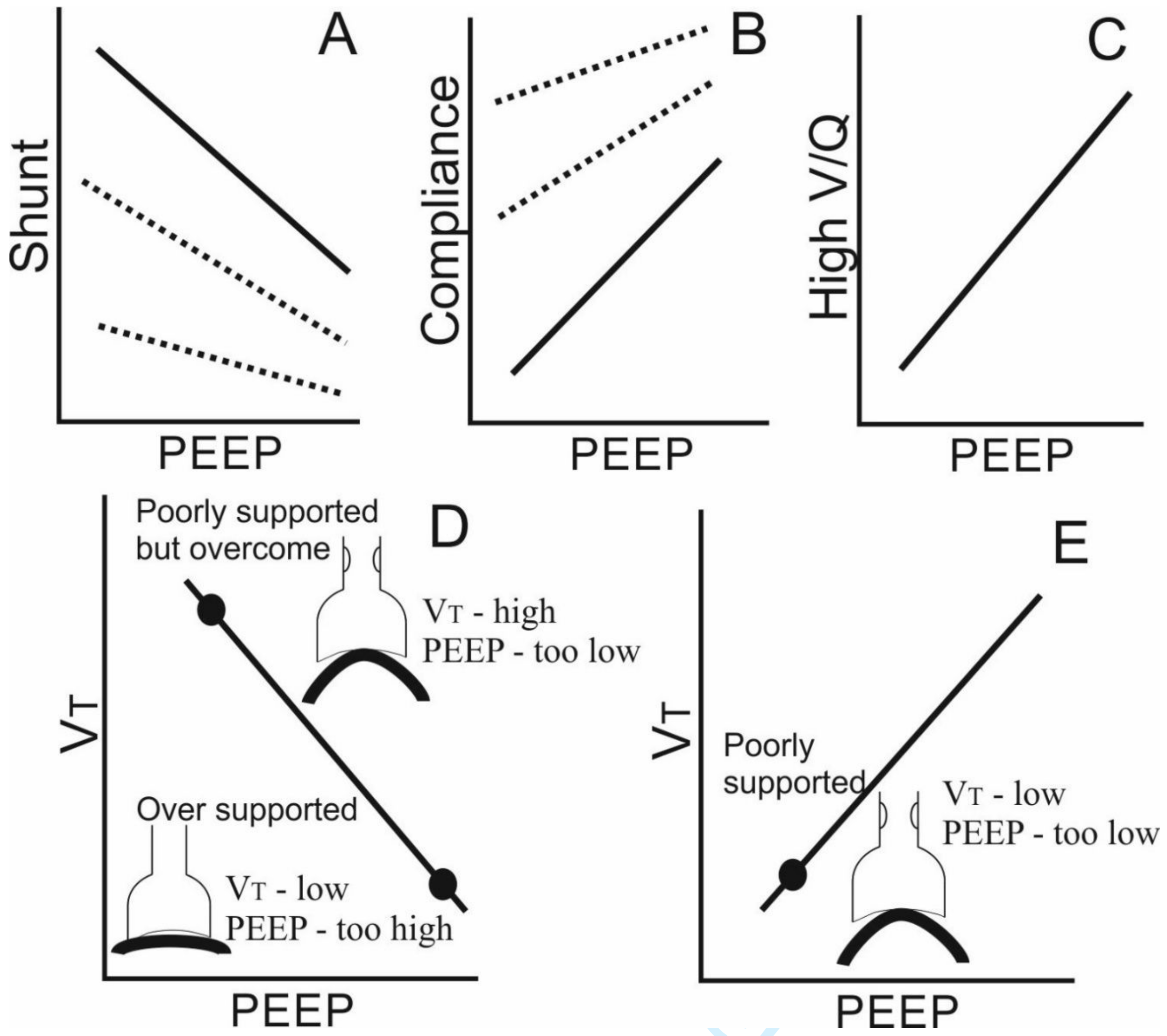

Figure E2: Mathematical models describing PEEP included in the Beacon Caresystem.

For peer review only - http://bmjopen.bmj.com/site/about/guidelines.xhtml

Use of the system

The system includes a tablet computer, plus a gas module including volumetric capnography and indirect calorimetry, with both mounted on the ventilator or a separate trolley. The patient is connected to the system via a respiratory tube measuring flow and with side stream sampling for measurement of O_2 and CO_2. The following text includes examples of advice generated by the system as reported in [1,2] beginning with the most simple examples. These examples are presentations of the screen of the tablet computer shown to the user.

In addition to these examples, it is important to note that on occasion the system will present advice to switch ventilator mode. This occurs if the patient is in a controlled ventilator mode and the system identifies a total respiratory frequency or a total minute ventilation that is significantly greater than that set, where the system provides a message to the user suggesting changing to support mode. Similarly, if the patient is in support mode and the model simulated pH is very low then the system provides a message asking the user to consider either control mode or reduction of anesthesia. In modes containing mandatory breaths initiated by the ventilator or by patient effort, and where different settings are available for patient initiated and ventilator initiated breaths, the system provides advice on these depending upon the pattern of patient ventilation, and whether the patient is currently in a period with substantial spontaneous breathing activity. The system functions on a variety of manufactures ventilators and, as illustrated in these examples, provides advice for a variety of modes. Current exceptions to these include APRV, and modes with automatic control of ventilator settings such as ASV© or SmartCare©. Data is collected automatically by the Beacon Caresystem, and includes tidal volumes, both set if the mode dictates, and also measured. The regulation of Vt and it relationship with mode can therefore be explored for both of the study arms. The system records all ventilator settings via connection to the ventilator, all direct measurements of flow and gas concentrations, its own SpO_2 measurement, and the timing and nature of all advice provided. The

For peer review only

For peer review only - http://bmjopen.bmj.com/site/about/guidelines.xhtml

extent to which advice has been followed and the effects of application of advice is therefore fully traceable.

For peer review only

For peer review only - http://bmjopen.bmj.com/site/about/guidelines.xhtml

Advice in pressure support mode ventilation.

Figure E3 illustrates a typical screen of the CDSS with advice to reduce PS and FIO_2. The hexagon on the right hand side of the screen visualizes the patient state in terms of the balances between over and under ventilation. The three vertical axes on the hexagon present the three balances between over and under oxygenation on the far right, (i.e. the compromise between the risks of oxygen toxicity and hypoxaemia, respectively); over and under ventilation as the central axis (i.e. the compromise between the risks of ventilator induced lung trauma and acidosis, respectively); and on the left the balance between over and under support (i.e. the balance between respiratory muscle atrophy due to over support, or patient stress due to under support). The blue symbol on the hexagon represents the patient's current state, and the grey symbol the simulated state according to the system's advice. The colour of the hexagon represents severity, with the most severe state represented as red, and the system automatically zooming in to yellow and green when possible. The left hand side of the screen shows both the current, simulated and advised settings. The white simulated blocks on the left are scroll wheels which allow the user to perform simulations of other levels of these ventilator settings.

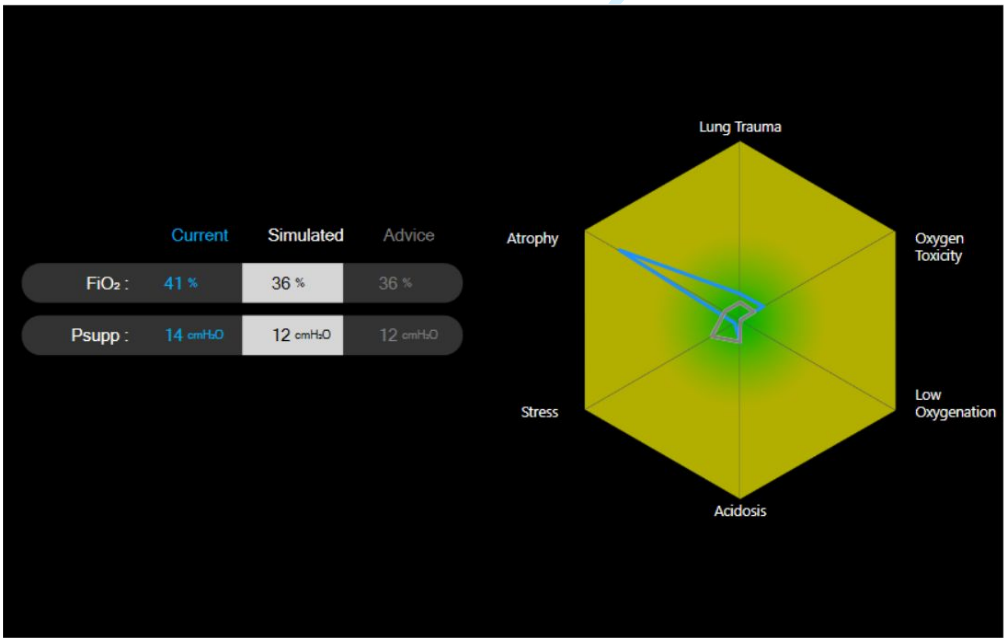

Figure E3 – User interface of the system, with advice to reduce FIO_2 and PS.

For peer review only - http://bmjopen.bmj.com/site/about/guidelines.xhtml

Figure E4 illustrates the same screen as E3, but with the corners of the hexagon activated to show the current, simulated and advised values of variables simulated by the physiological models. In this patient, the current FIO_2 setting of 41 %, results in a SaO_2 value of 97.9 % and a current simulated SvO_2 of 89.7 %. The system illustrates that the current balance between over and under oxygenation may be inappropriate with the blue symbol on the hexagon pointing slightly toward oxygen toxicity. The system therefore suggests reducing FIO_2. The system also illustrates an increased risk of respiratory muscle atrophy due to a low respiratory frequency, leading the system to suggest a reduction in PS from 14 to 12 cmH_2O, simulating that this may result in increased Rf.

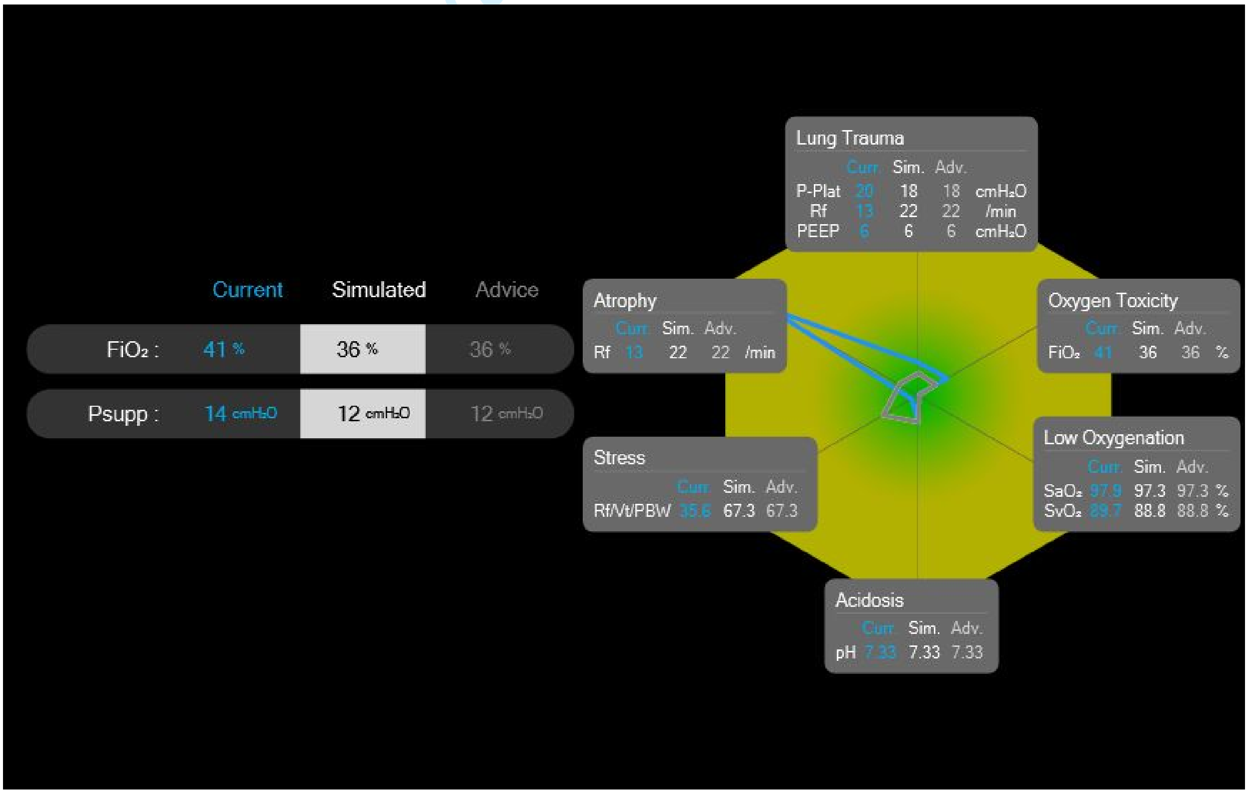

Figure E4 – User interface of the system, with advice to reduce FIO_2 and PS and corners of the hexagon activated to show model simulated physiological variables associated with the different conflicting clinical goals of mechanical ventilation.

For peer review only - http://bmjopen.bmj.com/site/about/guidelines.xhtml

Following several reductions in PS and FIO_2, the system advises on PEEP, illustrated in figure E5. The reduction of PS had less than the expected effect on increasing Rf, with the reduction in FIO_2 reducing oxygenation as expected. When exploring advice on PEEP, the CDSS also shows the simulated expected effect of PEEP, showing the simulated levels of shunt, low V/Q (ΔPO_2), high V/Q (ΔPCO_2), dead space (Vd) and dynamic compliance (COMP) at current, simulated and advised settings. The PEEP reduction was expected to have a limited effect on gas exchange parameters, with small increase in shunt, and a small decrease in dynamic compliance. As such, the advice was expected to result in a slight increase in Rf without a worsening in oxygenation.

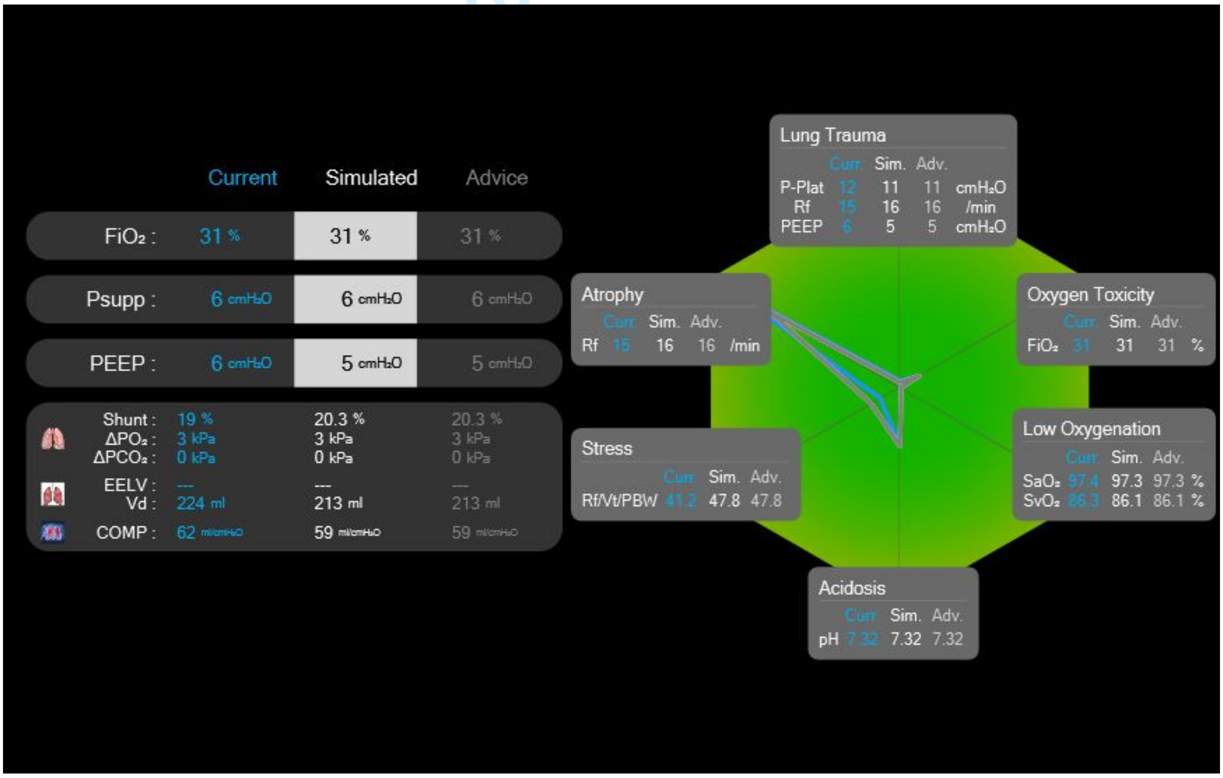

Figure E5 – User interface of the system, with an advice on PEEP.

For peer review only - http://bmjopen.bmj.com/site/about/guidelines.xhtml

Advice in control mode ventilaiton.

Figure E6 illustrates an advice including PEEP for a patient in pressure control ventilation. The vertical axis representing the balance between respiratory muscle atrophy and stress is disabled in controlled ventilation modes for cases where the patient has no spontaneous breathing activity. In this patient, the system indicated that the current balance between over and under ventilation might be inappropriate, with the blue symbol on the hexagon pointing toward lung trauma and with the patient being slightly alkalotic. The system suggested reducing PEEP and PC from 6 to 5 cmH_2O and 12 to 11 cmH_2O, respectively, and at the same time increasing Rf from 18 to 19 bpm. The simulated effect of PEEP was a negligible increase in pulmonary shunt, with simulated patient response to the combined advice being to reduce inspiratory pressure, reduce pH and maintain appropriate oxygenation.

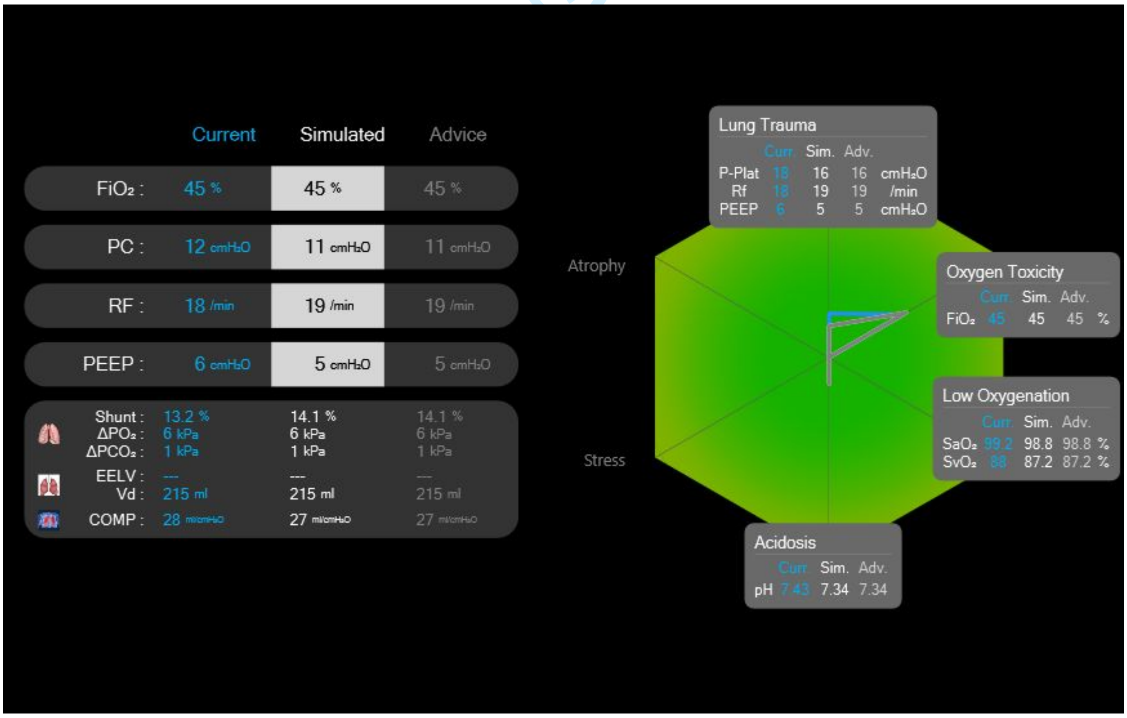

Figure E6 – User interface of the system illustrating an advice including PEEP in a patient ventilated in PC mode.

For peer review only - http://bmjopen.bmj.com/site/about/guidelines.xhtml

Figure E7 illustrates the result of following the advice illustrated in figure E8, and the subsequent advice provided by the system. The combined effect of PEEP and PC reduction and increase in Rf was as expected for both oxygenation and respiratory mechanics but less pronounced on pH. The advised level of PEEP was maintained in the next advice, but with an advice to decrease FIO_2 expecting a resulting safe oxygenation. Simulations in the details boxes at the hexagon corners take into account differences between set and measured Rf, where measured Rf was 17 bpm despite set Rf of 19 bpm.

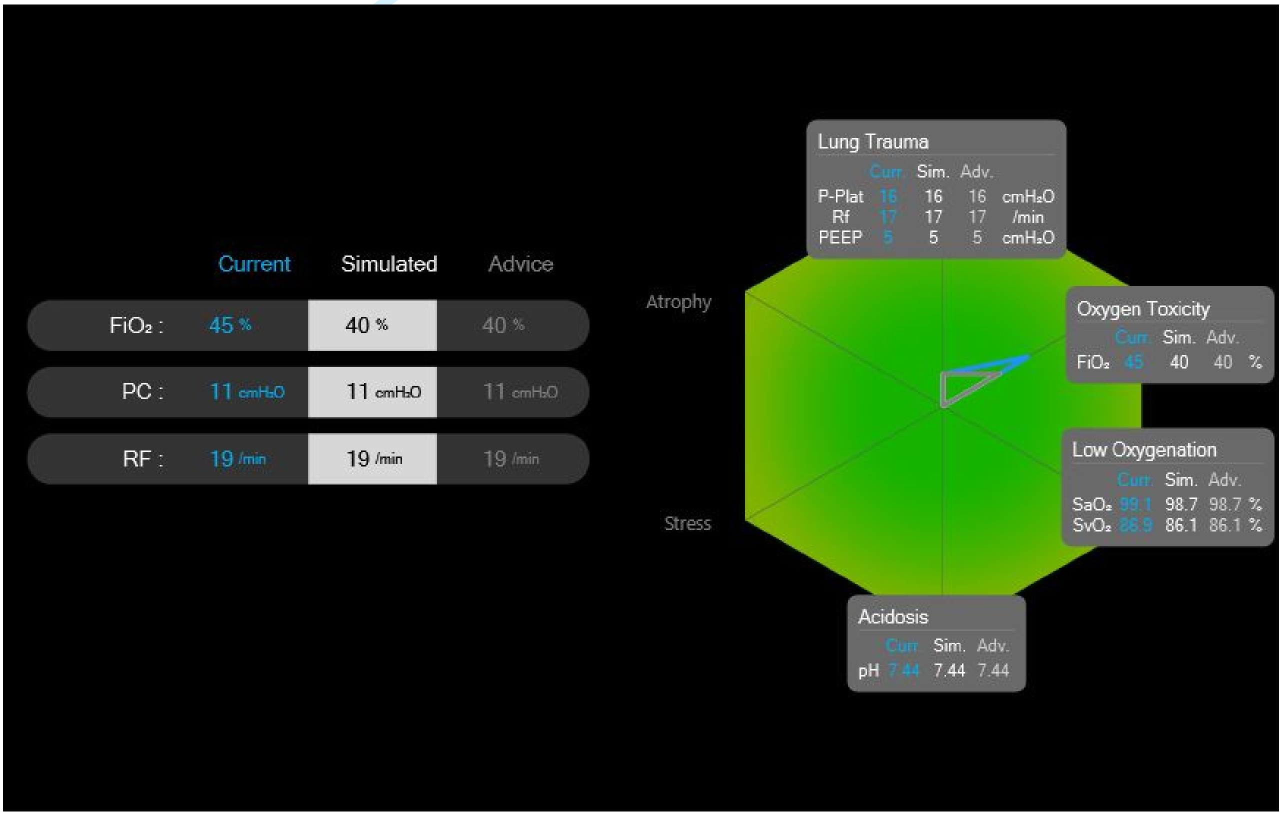

Figure E9 – User interface of the system, with results according to the advice in Figure E6, and the next advice.

For peer review only - http://bmjopen.bmj.com/site/about/guidelines.xhtml

SBT setting and review, and extubation check-list

For patients ventilated in pressure support mode with values of PEEP and PS below threshold values illustrated in figure E10, the system provides a counter on the screen which counts down from 30 minutes, and as such indicates that the patient has been within ventilator settings consistent with an SBT and stable with regard other respiratory measurements, for this duration. Cut-off values for these other variables are illustrated in figure E10

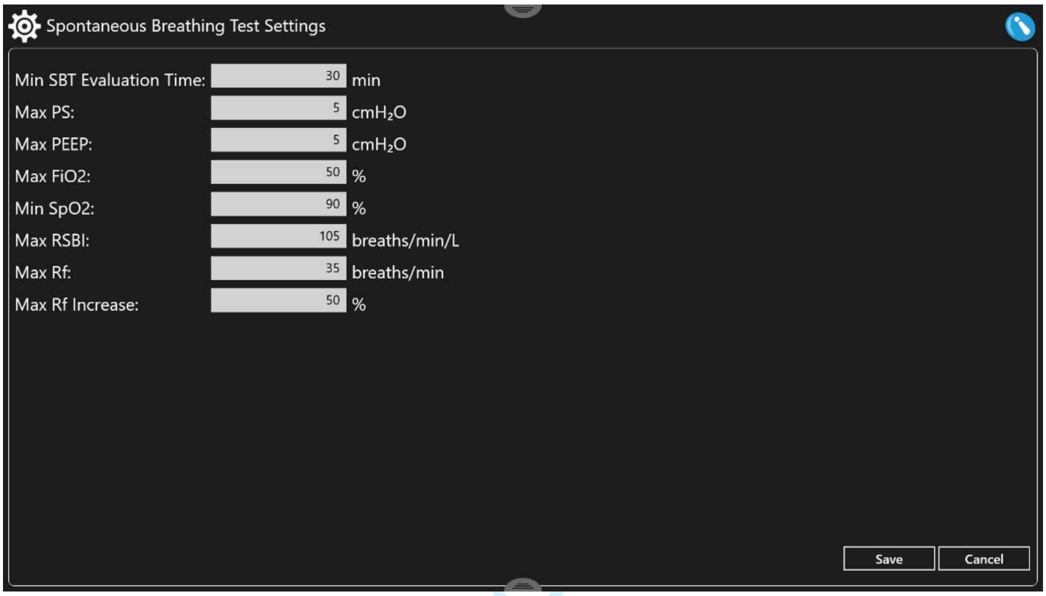

Figure E10 – User interface illustrating variables used to time the SBT counter of the system.

At any point when the counter is running, the user can view the screen illustrated in figure E11. This indicates which variables are within threshold, their values, and for how long this has been the case.

review only

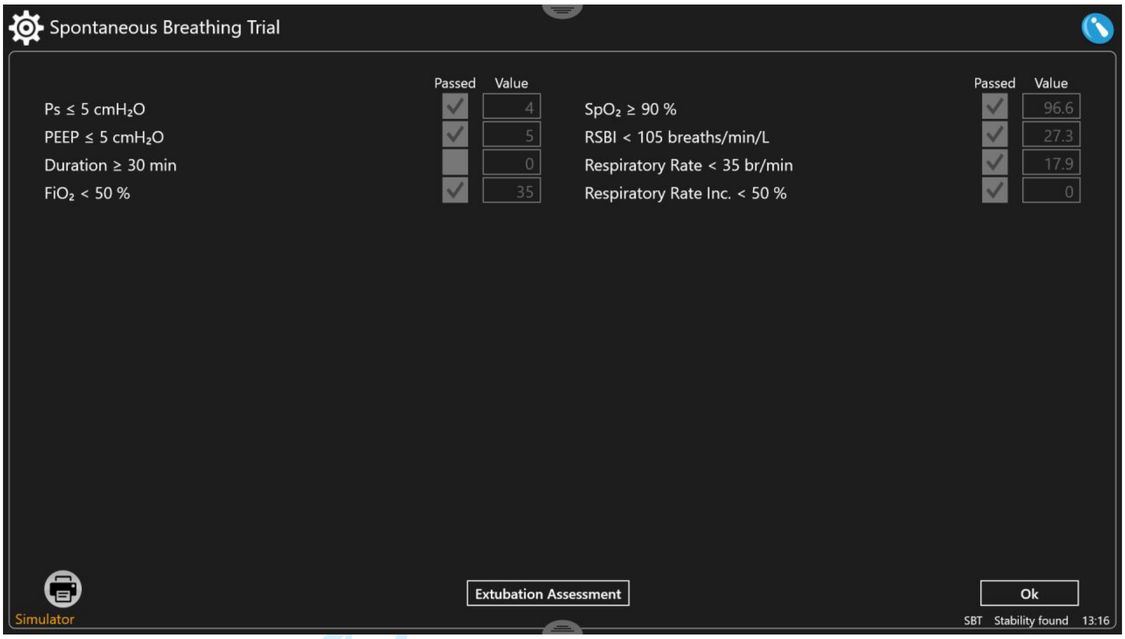

Figure E11 – User interface illustrating status of variables used to track the stability of patients over time in relation to the SBT counter.

When all variables illustrated in figure E11 are within range for the expected duration, the system allows checking of an extubation checklist illustrated in figure E12. Neither the SBT screens E10 and E11, nor the extubation checklist advise on an active SBT test or extubation.

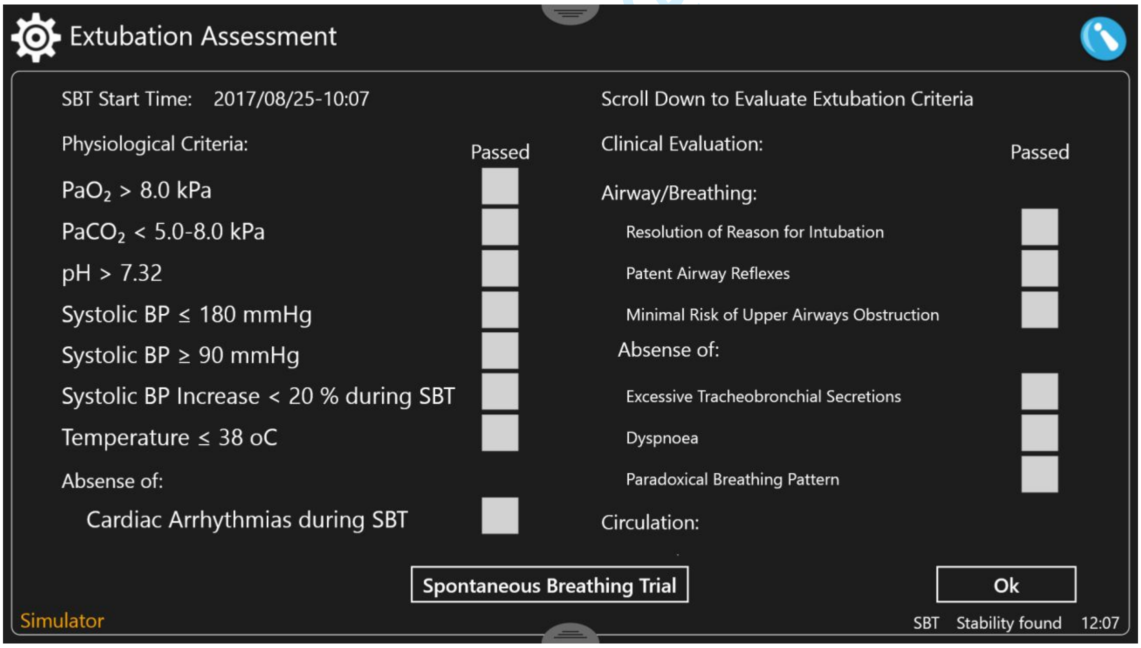

Figure E12a – Extubation checklist, top part of vertical scrolling window.

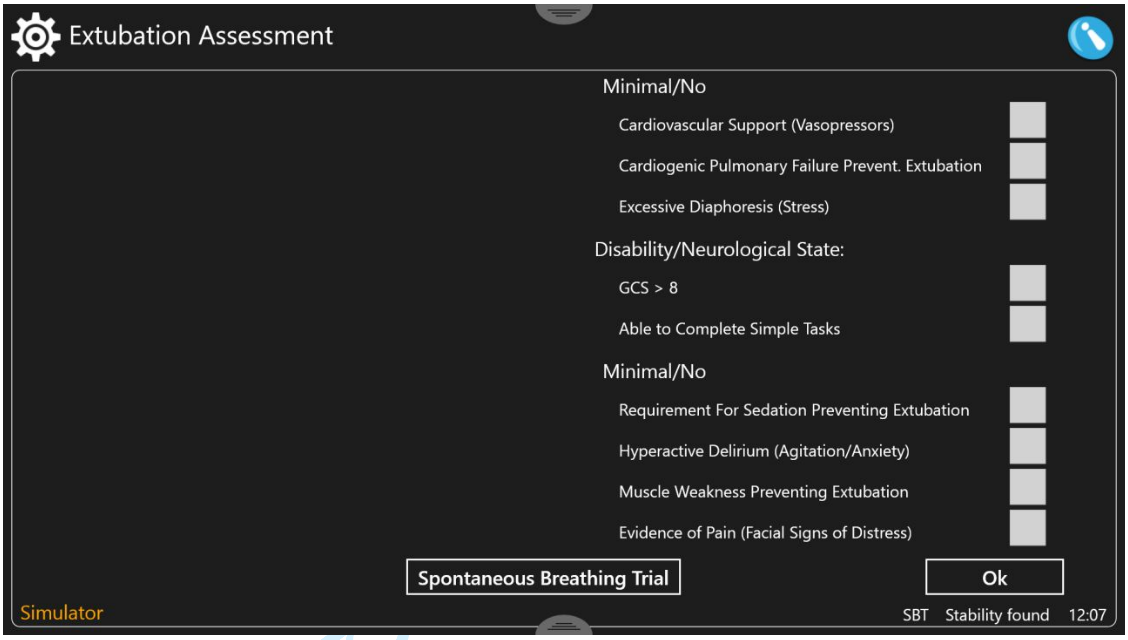

Figure E12b – Extubation checklist, bottom part of vertical scrolling window.

For peer review only - http://bmjopen.bmj.com/site/about/guidelines.xhtml

References

1. Karbing DS, Spadaro S, Dey N, Ragazzi R, Marangoni E, Dalla Corte F, Moro F, Lodahl D, Hansen NS, Winding R, Rees SE, Volta CA.. An Open-Loop, Physiologic Model-Based Decision Support System Can Provide Appropriate Ventilator Settings. Crit Care Med. 2018, 46(7):e642-e648

2. Spadaro S, Karbing DS, Dalla Corte F, Mauri T, Moro F, Gioia A, Volta CA, Rees SE. An open-loop, physiological model based decision support system can reduce pressure support while acting to preserve respiratory muscle function. J Crit. Care, 2018 Dec;48:407-413.

3. Rees SE, Karbing DS. Determining the appropriate model complexity for patient-specific advice on mechanical ventilation Biomed Tech (Berl). 201762(2):183-198.

4. Thomsen LP, Karbing DS, Smith BW, Murley D, Weinreich UM, Kjærgaard S, et al. Clinical refinement of the automatic lung parameter estimator (ALPE). J Clin Monit Comput. 2013;27(3):341-50.

For peer review only

BMJ Open

The iCareWean protocol on weaning from mechanical ventilation: A Single-blinded multi-centre randomised control trial comparing an open-loop decision support system and routine care, in the general Intensive Care Unit

Journal:	*BMJ Open*
Manuscript ID	bmjopen-2020-042145.R1
Article Type:	Protocol
Date Submitted by the Author:	09-Jul-2020
Complete List of Authors:	Vizcaychipi, M; Imperial College London, APMIC; Chelsea and Westminster Healthcare NHS Trust, Magill Department of Anaesthesia and Intensive Care Medicine<br>Martins, Laura; Chelsea and Westminster Hospital NHS Foundation Trust, Research Trial Unit<br>White, James; Chelsea and Westminster Hospital NHS Foundation Trust, Magill Department of Anaesthesia<br>Karbing, Dan; Aalborg Universitet, Center for Model-based Medical Decision Support<br>Gupta, Amandeep; West Middlesex University Hospital NHS Trust, Anaesthetic Department<br>Singh, Suveer; Chelsea and Westminster Hospital NHS Foundation Trust, Magill Department of Anaesthesia & Intensive Care medicine<br>Osman, Leyla; Chelsea and Westminster Healthcare NHS Trust, Magill Department of Anaesthetics<br>Moreno-Cuesta, Jeronimo; North Middlesex University Hospital NHS Trust, Anaesthetic Department<br>Rees , Steve ; Aalborg Universitet Institut for Medicin og Sundhedsteknologi
**Primary Subject Heading**:	Intensive care
Secondary Subject Heading:	Anaesthesia, Medical education and training, Respiratory medicine
Keywords:	Adult intensive & critical care < ANAESTHETICS, RESPIRATORY MEDICINE (see Thoracic Medicine), Physiology < BASIC SCIENCES

SCHOLARONE™
Manuscripts

I, the Submitting Author has the right to grant and does grant on behalf of all authors of the Work (as defined in the below author licence), an exclusive licence and/or a non-exclusive licence for contributions from authors who are: i) UK Crown employees; ii) where BMJ has agreed a CC-BY licence shall apply, and/or iii) in accordance with the terms applicable for US Federal Government officers or employees acting as part of their official duties; on a worldwide, perpetual, irrevocable, royalty-free basis to BMJ Publishing Group Ltd ("BMJ") its licensees and where the relevant Journal is co-owned by BMJ to the co-owners of the Journal, to publish the Work in this journal and any other BMJ products and to exploit all rights, as set out in our licence.

The Submitting Author accepts and understands that any supply made under these terms is made by BMJ to the Submitting Author unless you are acting as an employee on behalf of your employer or a postgraduate student of an affiliated institution which is paying any applicable article publishing charge ("APC") for Open Access articles. Where the Submitting Author wishes to make the Work available on an Open Access basis (and intends to pay the relevant APC), the terms of reuse of such Open Access shall be governed by a Creative Commons licence – details of these licences and which Creative Commons licence will apply to this Work are set out in our licence referred to above.

Other than as permitted in any relevant BMJ Author's Self Archiving Policies, I confirm this Work has not been accepted for publication elsewhere, is not being considered for publication elsewhere and does not duplicate material already published. I confirm all authors consent to publication of this Work and authorise the granting of this licence.

For peer review only

The iCareWean protocol on weaning from mechanical ventilation: A Single-blinded multi-centre randomised control trial comparing an open-loop decision support system and routine care, in the general Intensive Care Unit.

M. P. Vizcaychipi[1,5*], L. Martins[1] ,J.R. White[1], D.S Karbing[2], , A Gupta[3], S. Singh[1,5], L Osman[1], J. Moreno-Cuesta[4], S.E Rees[2].

1. Magill Department of Anaesthesia, Intensive Care Medicine and Pain Management, Chelsea and Westminster Hospital, C&W NHS Foundation Trust, London, UK.
2. Respiratory and Critical Care group (rcare). Department of Health Science and Technology. Aalborg University, Denmark.
3. Department of Anaesthesia and Intensive Care Medicine, West Middlesex University Hospital, C&W NHS Foundation Trust, London, UK
4. Department of Intensive Care Medicine, North Middlesex University Hospital, London, UK
5. Academic Department of Anaesthesia, Intensive Care & Pain Management, Imperial College London, UK.

**Corresponding Author: Dr Marcela P. Vizcaychipi, email:* m.vizcaychipi@imperial.ac.uk

For peer review only

Abstract

Introduction: Automated systems for ventilator management to date have been either fully heuristic rule-based systems or based on a combination of simple physiological models and rules. These have been shown to reduce the duration of mechanical ventilation in simple to wean patients. At present there are no published studies that evaluate the effect of systems that utilise detailed physiological descriptions of the individual patient.

The BEACON Caresystem© is a model-based decision support system that utilises mathematical models of patients' physiology in combination with models of clinical preferences to provide advice on appropriate ventilator settings. An individual physiological description may be particularly advantageous in selecting the appropriate therapy for a complex, heterogeneous, ICU patient population.

Methods & Analysis: iCareWean is a single-blinded, multicentre, prospective randomised control trial evaluating management of mechanical ventilation as directed by the BEACON Caresystem© compared to that of current care, in the general intensive care setting. The trial will enrol 274 participants across multiple London NHS Intensive Care Units. The trial will utilise a primary outcome of duration of mechanical ventilation until successful extubation.

Ethics & dissemination: Safety oversight will be under the direction of an independent committee of the study sponsor. Study approval was obtained from the regional ethics committee of the HRA, REC reference: 17/LO/0887. IRAS reference: 226610. Results will be disseminated through international critical care conference/symposium and publication in peer-reviewed journal.

Trial Registration details: ClinicalTrials.gov under NCT03249623. This research is registered with the National Institute for Health Research (NIHR) under CPMS ID: 34831.

Keywords: Mechanical ventilation, spontaneous breathing trial, extubation, liberation, weaning, open-loop decision support device, physiological model.

For peer review only

Strengths and limitations of this study

- Single-blinded multi-centre randomised control trial evaluating management of mechanical ventilation as directed by the BEACON Caresystem© compared to that of standard care in the general intensive care setting.
- The BEACON Caresystem© is a model-based decision support system that utilises mathematical models of patients' physiology in combination with models of clinical preferences to provide advice on appropriate ventilator settings.
- Sample size was determined by a power analysis of the duration of mechanical ventilation.
- Patients and the public were not involved in the design of this study.

The study does not assess successful rehabilitation of the patient subsequent to their ICU stay.

For peer review only

Introduction

Optimising mechanical ventilator management has been the focus of much research, seeking to decrease ventilator-associated complications such as ventilator-associated lung injury, ventilator-associated pneumonia, respiratory muscle atrophy, and patient discomfort [1, 2]. Increased duration of mechanical ventilation and prolonged weaning, increases the number of these complications with a vicious cycle that leads to increased morbidity and mortality and associated economic costs [2]. Protocols and computerised tools aiming to reduce length of mechanical ventilation might therefore be beneficial.

Weaning protocols and nurse-lead ventilator management is routinely used in the intensive care setting [2, 3]. Such approaches have been shown to reduce the duration of mechanical ventilation [3]. However, standardising this process is important, and the quality and quantity of nursing care is a critical factor in reducing duration of mechanical ventilation [4].

Standardisation of care through automation or bedside decision support continues to be driven by novel technological innovation, some of which is already established in clinical practice [3, 5]. However, to date much of this has been predicated on clinical guidelines rather than utilising detailed physiological descriptions of the individual patient [6 - 8]. A Cochrane review of weaning trials with current systems concluded that their use may reduce duration of weaning, but highlighted that the population was predominantly composed of those who are “simple to wean” [5]. The patient populations of these studies were characterised as without additional lung pathology and were predominantly ventilated for periods of less than 48 hours [5]. Consequently, it remains to be investigated whether decision support systems can reduce duration of mechanical ventilation in patients with more than a few hours of ‘routine’ postoperative intubation. The BEACON Caresystem© is a model-based decision support system that utilises mathematical models of patients’ physiology in combination with models of clinical preferences to provide advice on appropriate ventilator settings [9]. An individual physiological description may be particularly advantageous in selecting the appropriate therapy for a complex, heterogeneous, ICU patient population. The BEACON Caresystem© is the commercial version of the INVENT system, which has been retrospectively evaluated in post-operative cardiac patients and patients with severe lung disease, and prospectively evaluated in providing advice on correct levels of inspired oxygen [10-13]. To date, the commercial system has been evaluated over short durations of time from 4-8 hours to establish whether physiological measurements are improved [14, 15]. No previous studies evaluating outcome of weaning from mechanical ventilation have been performed.

Here we describe a single-blinded multi-centre randomised control trial evaluating management of mechanical ventilation as directed by the BEACON Caresystem© compared to that of current care in the general intensive care setting.

For peer review only

Methods and Analysis

Administrative Structure

The study is under the auspices of the eHRA, REC reference: 17/LO/0887, IRAS reference: 226610, and is registered at ClinicalTrials.gov under NCT03249623, and with the National Institute for Health Research (NIHR) under CPMS ID: 34831. The Research and Development department of the Chelsea and Westminster NHS Foundation Trust, is sponsor of the study and responsible for study conduct, safety monitoring, personnel training, and randomisation. Data collection occurs at participating centers, with data analysis performed in collaboration with the respiratory and critical care group (rcare), Aalborg University, and independent statisticians of the study sponsor. All data transfer and storage will be performed in accordance with GDPR regulations and the study will monitor and review interim analysis and made recommendations to the steering committee.

Study design

To test the hypothesis that following advice from the BEACON Caresystem© will reduce time to successful extubation, the iCareWean group has designed a single-blinded multi-centre randomised control trial evaluating management of mechanical ventilation as directed by the BEACON Caresystem© compared to that of current care in the general intensive care setting. The Consort flow diagram of the study is illustrated in figure 1.

Description of the BEACON Caresystem©

At the core functionality of the BEACON Caresystem© is a set of physiological models including models of pulmonary gas exchange, acid-base chemistry, lung mechanics, and respiratory drive [9]. The BEACON Caresystem© tunes these models to the individual patient so that they accurately describe the current physiological measurements. Once the models are tuned, they are then used by the system to simulate the effects of a change in ventilator settings. The results of these simulations are then used to calculate the clinical benefit of changing the ventilator settings by balancing the competing goals of mechanical ventilation using mathematical models of clinical preference [9,10]. Appropriate ventilator settings imply a balance between quantified preferences of several competing clinical goals. For oxygenation, the preferred values of inspired oxygen fraction is weighed against the need to prevent hypoxaemia. For settings of ventilation volume, pressures or frequency, the risk of lung trauma is weighed against the need to prevent acidosis. For settings of support in spontaneously breathing patients, the risk of respiratory muscle atrophy on over support is weighed against the excessive work of breathing and potential of respiratory muscle fatigue on under support. Quantification of these risks is used to calculate a total score for any possible ventilator strategy. The system then calculates advice so as to change ventilator settings to improve this score.

The BEACON Caresystem© functions as an “open-loop” system. This means that the advice provided by the system is presented to the clinician. The ventilator settings are then changed by the clinician, and the patient’s response to these changes are automatically registered and used by the BEACON Caresystem© to re-tune the physiological models and repeat the process of generating new advice.

For peer review only

The BEACON Caresystem© provides advice on ventilator settings including the following. Values for maximal advice steps are given in table 1.

- Fraction of inspired oxygen (FiO_2)
- Tidal volume (Vt)
- Respiratory frequency (f)
- Positive end expiratory pressure (PEEP)
- Pressure above PEEP

Table 1. Maximal steps in mechanical ventilation advice provided by BEACON Caresystem©

Maximal allowed step in ventilator settings	Stepping up	Stepping down
FIO_2	10 %	5% or 2% when $SpO_2 \leq$ 92 %
Vt (volume modes)	50 ml	50 ml
Respiratory frequency (f)	5 breaths/min or 3 breaths/min when f ≥ 18	5 breaths/min
Pressure above PEEP (pressure modes)	2 cm H_2O	2 cm H_2O
PEEP	3 cm H_20 if PEEP < 10 cm H_20, otherwise 2 cm H_20	2 cm H_2O

Patient Screening

All patients will be screened for eligibility upon admission to the ICU by the Research team, including research nurses and clinical research fellows. Advice will be obtained from relatives acting as a personal consultee, or Consultant acting as nominated professional consultee. Retrospective consent is then obtained from the patient upon regaining capacity. The study will run from September 2017 to November 2020.

Eligibility Criteria

Inclusion criteria:

1. At least 24 hours of invasive mechanical ventilation.
2. Age ≥ 18 years.
3. Patient is mechanically ventilated in a ventilator mode, and by a ventilator, supported by the BEACON Caresystem©.
4. Haemodynamically stable, where instability is defined by the presence of two or more of the following criteria: acidosis pH < 7.2, poor urine output < 0.5ml/kg/h, use of vasopressors, e.g. noradrenaline > 25 µg/min.

Exclusion criteria:

1. The absence of an arterial catheter for blood sampling.

For peer review only

2. Medical history of home mechanical ventilation which may lead to prolonged stay in the ICU, including long term oxygen therapy and non-invasive ventilation not associated with sleep apnoea.
3. Clinical conditions requiring treatment with ECMO, i.e. an inspired oxygen of 100% for more than 24 hours.
4. Head trauma or other conditions where intra-cranial pressure may be elevated and tight regulation of arterial CO_2 level is paramount.
5. Primary (non-overdose related) neurological patients (Glasgow coma score 24).
6. Severe heart failure classified by grade 4 American Heart Association guidelines.
7. End stage liver disease.
8. Multiple ICU admissions within the medical stratification group, i.e. more than one admission.
9. Corrective orthognathic surgery.
10. ICU admission following oesophageal surgery.
11. Morbidly obese patients defined as either BMI ≥45; or BMI ≥35 with an APACHEII score on admission >24.
12. Pregnancy.
13. Mechanical ventilation initiated for more than 24 hours in other centres.

Randomisation

Figure 1 illustrates the Consort flow diagram for the study. Upon recruitment to the trial, participants are randomised to either to the intervention group where advice is provided by the BEACON Caresystem©, or to the control arm where standard care is provided. Block randomisation is applied with each block stratified to ensure equal distribution between control and intervention arm for the following patient groups, reducing the risk of potential bias by overrepresentation of these groups in the control or intervention arms:

- Surgical or medical ICU admission.
- Presence of absence of chronic obstructive pulmonary disease (COPD).
- Primary thoracic or non-thoracic medical diagnosis.

Study Procedure

In the intervention arm of the trial, the BEACON Caresystem© is activated by the research team and subsequent ventilator management determined by system advice, applied to the ventilator by the clinical team, as illustrated in figure 2 and described below. In the control arm, the BEACON Caresystem© is activated in a mode in which system advice is disabled, but data is still collected by the BEACON Caresystem©. The ventilator management in this arm is determined by standard care.

Intervention arm

Figure 2 illustrates the protocol flow in the intervention arm. At any time during the protocol, adverse events are addressed immediately, with a serious event resulting in exclusion from the study (illustrated as B in figure 2). Minor events are documented and the patient returned to the main protocol flow (illustrated at point A, at the top of figure 2). During intervention, the BEACON Caresystem© can provide a number of different forms of advice to the user which require action. These are illustrated at the top of figure 2, and are described in turn below.

For peer review only

ABG/ALPE - In situations where mathematical model simulations cannot accurately describe continuous measurements, i.e. SpO_2, end-tidal CO_2 ($FetCO_2$), or in support ventilator modes respiratory frequency or tidal volume, then retuning of the models is required. In this situation, the BEACON Caresystem© requests either an arterial blood gas (ABG), or a short procedure varying FiO_2 to assess pulmonary gas exchange (Automatic Lung Parameter Estimator - ALPE), as described and evaluated previously [16-20], and briefly described in the electronic supplementary material.

Ventilator - Advice on changing ventilator settings. New advice is generated only when the system regards current settings as not appropriate. Duration between advice therefore varies, however for PEEP and changes in volume or pressure 10-20 minutes is the minimum period between advice.

Bounds - The BEACON Caresystem© allows for bounds to be set for the limits of advice on ventilator settings. If advice is available which is outside these bounds, the protocol dictates that the bedside nurse consults with the clinician on duty.

Mode/sedation - If the BEACON Caresystem© detects substantial spontaneous breathing during control ventilator modes, advice is provided to consider changing to support mode. Additionally, if pH falls below a defined value in support mode, advice is provided to consider changing to control mode or modifying sedation. In either situation the protocol dictates that the bedside nurse consults with the clinician on duty.

Spontaneous Breathing Trial (SBT) - During weaning from mechanical ventilation the BEACON Caresystem© monitors the levels of PS and PEEP. When these levels are reduced to values below clinical guideline thresholds [21], the BEACON Caresystem© automatically monitors variables evaluating spontaneous breathing criteria. If these remain within limits and for a duration determined by international guidelines [21], the system prompts the user to assess the appropriateness of extubation and provides a checklist to help. The primary function of the BEACON Caresystem© with regards weaning is to reduce levels of support where possible, it does not suggest an active SBT or extubation. As such, the decision to extubate is according to standard clinical practice for both arms of the study, meaning that at any time the clinical team can perform an active SBT or extubate the patient without confirmation from the system. Reintubation is documented by the research team and the participant enters a standard care pathway, which involves the deactivation of the BEACON Caresystem©. The participant is then followed up until the end of the protocol (Figure 1).

The protocol ends with transfer or discharge from ICU or death (Figure 1).

Primary & Secondary Outcomes

The primary outcomes include:

- Duration of mechanical ventilation to successful extubation defined as the time from the start of mechanical ventilation, defined as either the time of intubation in the ICU, or the time of admission to the ICU following previous intubation for surgery, and until successful extubation, with successful extubation defined as ≥48 hours of unassisted spontaneous breathing after extubation.

For peer review only - http://bmjopen.bmj.com/site/about/guidelines.xhtml

Unassisted spontaneous breathing is defined here to include supplementary oxygen and support with high flow nasal catheters. Patients with non-invasive mask ventilation are considered to be assisted.

The secondary objectives of the trial are to compare the following variables in the standard care group and BEACON Caresystem© group:

- Duration of mechanical ventilation to successful extubation following randomisation defined as the time from randomisation and until successful extubation, with successful extubation defined as ≥48 hours of unassisted spontaneous breathing after extubation.
- Time from control mode to support mode defined as the time following randomisation, from initiation of control modes of ventilation and until initiation of support modes of ventilation.
- Time from support mode to successful extubation defined as the time following randomisation, from initiation of support modes of ventilation and until successful extubation.
- Number of changes in ventilator settings per day defined as the daily registered number of changes to ventilator settings from randomisation until successful extubation.
- Time to first SBT defined as the time from randomisation to the first performed SBT.
- Time to first successful SBT defined as the time from randomisation to the first successful SBT.
- Time to first extubation defined as the time from randomisation to the first extubation attempt.
- Time to first disconnection from mechanical ventilation defined as the time from randomisation to the point at which mechanical ventilation is first discontinued.
- Percentage of time in control mode ventilation defined as the time from randomisation which is spent in controlled modes of mechanical ventilation in percent of duration of mechanical ventilation.
- Percentage of time in support mode ventilation defined as the time from randomisation which is spent in support modes of mechanical ventilation in percent of duration of mechanical ventilation.
- Use of sedatives defined as the cumulative use of sedative drugs from randomisation until successful extubation.
- Use of neuromuscular blockading agents defined as the cumulative use of neuromuscular blockading agents from randomisation until successful extubation.
- Number of intubation free days defined as the number of days without intubation from randomisation until successful extubation.
- Number of reintubations defined as the number of reintubations following extubation from randomisation until successful extubation.
- Number of tracheostomies defined as the number of patients having tracheostomy performed from randomisation until successful extubation or protocol end.
- Number of patients on prolonged mechanical ventilation defined as the number of patients on mechanical ventilation ongoing for more than 21 days after the start of mechanical ventilation.
- Number and types of adverse events related to mechanical ventilation defined as the incidence of adverse events directly related to mechanical ventilation.
- ICU mortality defined as the mortality from randomisation and until death or ICU discharge.
- Length of stay defined as the duration of ICU admission from the start of mechanical ventilation and until death or ICU discharge.

For peer review only

- Mobilization times defined as the time from randomisation until a) first mobilization, e.g. sitting on the edge of the bed, standing up and marching on the spot; and b) regaining independency, e.g. able to drink/eat or comb hair.
- Daily patient physiological blood gas status defined as daily arterial blood gas values of SaO_2, PaO_2, $PaCO_2$, pH from randomisation until successful extubation.
- Daily average physiological status defined as daily averages of measured values of oxygenation (SpO_2), end-tidal CO_2 fraction ($EtCO_2$), ventilation (respiratory rate, tidal volume, anatomical dead space), pulmonary mechanics (Airway pressure, respiratory system compliance), and ventilator settings as measured routinely at the bedside.

Statistical considerations

Sample size was determined by a power analysis of duration of mechanical ventilation. Extrapolation of the 2016 ICU admission data from two London based NHS hospitals provided a sample size of 548 patients over an 18 month period. In these patients, mean total duration of mechanical ventilation measured in days was 7.2, with a standard deviation of 7.0 days. A study population of 137 patients in each randomisation group, would allow detection of a 2 day or 30% improvement in the duration of mechanical ventilation in the BEACON Caresystem© group assuming a similar improvement in SD, with an alpha level of 0.05 and power of 0.8. A 2 day or 30% reduction in duration of mechanical ventilation is similar to that used previously [22].

Primary and secondary outcomes will be measured across the protocol duration and comparison made by independent samples *t*-test, or appropriate non-parametric testing if not normally distributed. Complementary analysis will be performed using Kaplan-Meier curves.

The study is not sufficiently powered to allow subgroup analysis for the groups specified in the randomisation strategy

An independent data safety monitoring committee will be established as part of the study, with this committee responsible for defining stopping criteria. Safety data will be looked at after the first 80 patients have been recruited, with an interim analysis performed following 50% recruitment.

Safety, Ethics and Dissemination

Adverse events

All adverse events will be monitored and documented until the end of study. Adverse events are reported to the trial Sponsor according to their standard operating procedure for further investigation. As patients are critically ill it is expected that unrelated adverse events will occur as part of hospital stay. Adverse events are therefore only defined in relation to changes in mechanical ventilation, for patients allocated to either the control or intervention arms.

1. Serious adverse events are defined as:
    - Death, suspected to be caused directly by the settings of mechanical ventilation.

2. Adverse events include the following events suspected to be caused directly by the settings of mechanical ventilation:
    - Pneumothorax
    - Self-extubation
    - Hypoxaemia (SpO2 <90%; PaO2 <8.00 kPa)
    - Alkalaemia (pH >7.5)

For peer review only

- Acidaemia (pH <7.2)

Safety Considerations, systems advice

The potential risks and safety considerations related to the use of the The BEACON Caresystem© have been considered and include:

1. Inappropriate suggestions for changes in ventilator settings

Inappropriate changes in ventilator settings, if applied, can have serious consequences. The general strategy employed in this protocol has three components:

i. The BEACON Caresystem© only provides advice in the situation where the models can accurately describe all psychological measurements. The ability of BEACON Caresystem© to do so is automatically assessed by the system throughout the protocol and failure to do so means that no advice is provided, which is made visible to the user.
ii. Maximal limits have been applied to the amount for which each ventilator setting can be changed in a single advice, meaning that inappropriate advice should have a reduced impact. The maximal advice changes for each ventilator setting are given in Table 1.
iii. The risks of changing each of the ventilator settings have been considered individually. Low values of inspiratory pressure or volume can result in reduction of minute ventilation and increase in $PaCO_2$ and consequently a fall in arterial pH. To assess this, CO_2 is monitored continuously by the BEACON Caresystem© using capnography. High values of inspiratory pressure can result in lung damage. Peak inspiratory pressure (PIP) is monitored continuously by the BEACON Caresystem©, and all mechanical ventilators provide alarms at high values. Low values of respiratory frequency have no apparent detriment other than those specified in reduction of volume. High values of respiratory frequency can result in air trapping. The maximum step in respiratory frequency is limited to 3 breaths/min if the current frequency is ≥ 18 allowing the onset of trapping to be identified clinically.
iv. Although there are small long term risks associated with increasing inspiratory oxygen due to absorption atelectasis or oxygen toxicity, the major acute risk of changes in inspired oxygenation is arterial hypoxaemia. If the current SpO_2 ≤92%, then the maximum reduction in FiO_2 is limited to 2%. In addition, pulse oximetry is monitored continuously by the BEACON Caresystem© during the study and an alarm sounded if levels are ≤90%.

To encourage use of the system and perform an evaluation where advice is applied, the application of advice is mandatory. As clinical practice is variable and advice is unlikely to be applied in all cases, the application and effects of advice are monitored by the Beacon Caresystem, and can be interrogated to ensure that proposed changes have been made and that the effects are considered safe in cases of doubt.

2. System malfunction

In the event of system malfunction the patient enters standard care until the malfunction is detected and corrected by biomedical engineers.

3. Sampling and measurement of arterial blood gas values

Arterial blood gas sampling is part of routine ICU care. Additional arterial blood gas sampling may be required by the BEACON Caresystem© than those performed in clinical practice. The BEACON Caresystem© requirements, estimated from current experience, are expected to be a maximum of 6 per day in the intervention group.

For peer review only

All other measurements used are obtained from non-invasive means including pulse oximetry and measurement of respiratory gas content, flow and pressure.

Ethical approval and Consent

Study approval was obtained from the regional ethics committee of the HRA, REC reference: 17/LO/0887. IRAS reference: 226610. The majority of the trial population lack the capacity to consent. Therefore, participant information sheets (PIS) and advice is requested from a third party acting as a consultee; in most cases this person will be a Personal Consultee, who is someone who knows the person lacking capacity and is able to advise the researcher about that person's wishes and feelings in relation to the project and whether they should join the research. However, where the personal consultee is not available, the researcher may nominate a professional person to assist in determining the participation of a person who lacks capacity. A Nominated Professional Consultee will be appointed by the researcher to provide advice regarding the patient's suitability to participation in the research. In the event that a personal consultee is identified after the nominated professional consultee advice has been obtained, the above process for personal consultee declaration will be followed. A patient information sheet will be distributed immediately following the patient being identified as eligible for the study. If a surviving patient regains capacity, they will be approached by the research team to obtain their consent to continue in the study. If the patient refuses consent or withdraws the intervention will be stopped but the regular/expected medical care will still be provided. Without consent, no additional information about the patient will be collected for the purposes of the study. However, to maintain integrity of the randomised trial, all information collected up to that time will still be used and analysed as part of the study.

Dissemination Plan

The dissemination strategy will involve the presentation of the results at international critical care conferences and publication in a peer-reviewed journal.

Patient and public involvement

Patients and the public were not involved in the design of this study. Patients randomised into the study will be informed of the results of the study once the outcome of the study is published.

Discussion

This paper presents the study protocol of single-blinded multi-centre randomised control trial evaluating management of mechanical ventilation as directed by the BEACON Caresystem© compared to that of standard care in the general intensive care setting. To test the hypothesis that following advice from the BEACON Caresystem© will reduce time to successful extubation, patients are randomised to care including advice from the system, or standard care. In addition, as secondary outcomes, the study will investigate whether application of the advice changes the time spent in control ventilation modes, the number of changes made to the ventilator, the use of medication such as sedative or neuromuscular blocking agents, the changes in clinical procedures such as re-intubation or tracheostomy, and clinical and physiological status of the patient. Although not powered to detect differences between groups, it will also investigate the length of stay at the ICU and patient mortality.

For peer review only

This study is the next step in evaluation of the advice of the BEACON Caresystem©. A large number of studies have been performed validating the physiological models included in the system, as summarized in [9], and studies of short duration have been performed evaluating the physiological response to application of advice [14,15]. This use of the system over the whole duration of ICU stay allows for assessment of more meaningful clinical outcomes, relating to the time spent on mechanical ventilation. In addition, the study includes only patients intubated for a minimum of 24 hours, meaning that short term post-operative patients who may have rather uncomplicated intubation periods, are not included.

The study includes limitations. The study was powered on duration of mechanical ventilation rather than duration to successful ventilation, this being the historical data available during study design. The study is not powered to detect changes in mortality, and does not follow up on the patients after transfer from the ICU. In the context of this study, successful extubation is defined as the lack of need for non-invasive ventilator support for 48 hours after extubation, rather than successful rehabilitation of the patient subsequent to their ICU stay.

The results of this study compare care provided with the system's advice with current clinical practice at study sites. As there is no standard of care or usual method of weaning in the United Kingdom, this study does not compare the system's advice with that of best practice defined by clinical guidelines. To avoid comparing two interventional changes, no attempt will be made to standardize care in the control arm according to best practice.

As patients are studied for many days it is not possible to store continuous waveform data for all patients at all times. Systematic analysis of patient-ventilator asynchrony will not therefore be possible.

This study excluded patients if ventilated for <24 hours. In doing so, patients ventilated post-surgery defined previously as "simple to wean" are not included. This should not imply that the patients studied here are necessarily difficult to wean, but rather that they represent a broad cross-section of mechanically ventilated patients not simply recovering from the effects of anaesthesia.

In addition, it is important to note that substantial training has been provided to all sites in the use of the BEACON Caresystem© and the same clinical team treat patients for both the control and intervention arm. It is possible then that both training and use of the system influence routine care, potentially biasing study results.

This paper describes the study protocol of a randomised control trial evaluating the advice of the BEACON Caresystem© in a general medical ICU population. This protocol represents an important step evaluating the use of advice over the whole of the ICU stay, and only in patients intubated greater than 24 hours. The results will indicate whether the BEACON Caresystem© is a useful tool in optimising ventilation strategy.

Acknowledgements:

The authors would like to thank Dr Sundhiya Mandelia, Senior statistician for reviewing the method section of this protocol. Mr Damon Foster and Dr Essam Ramhamadany for their support and contributions to the setup of the clinical study. The critical care nurses and physicians working in the NHS who feedback on the protocol.

For peer review only

For peer review only

References:

1. The ARDS Network. Ventilation with Lower tidal volumes as compared with traditional tidal volumes for acute lung injury and acute respiratory distress syndrome. *NEJM* 2004; 342: 1301-1308.
2. Pham T, Brochard LJ, Slutsky AS. Mechanical Ventilation: State of the Art. *Mayo Clin Proc* 2017; 92(9):1382-1400.
3. Blackwood B, Alderdice F, Burns K, Cardwell C, Lavery G, O'Halloran P. Use of weaning protocols for reducing duration of mechanical ventilation in critically ill adult patients: Cochrane systematic review and meta-analysis. *BMJ* 2011; 342:c7238.
4. Danckers M, Grosu H, Jean R, Cruz RB, Fidellaga A, Han Q, Awerbuch E, Jadhav N, Rose K, Khouli H. Nurse-driven, protocol-directed weaning from mechanical ventilation improves clinical outcomes and is well accepted by intensive care physicians. *J Crit Care* 2013;28:433-41.
5. Rose L, Schultz MJ, Cardwell CR, Jouvet P, McAuley DF, Blackwood B. Automated versus non-automated weaning for reducing the duration of mechanical ventilation for critically ill adults and children. *Cochrane Database of Systematic Reviews* 2014; 6: CD009235.
6. Lellouche F, Mancebo J, Jolliet P, Roeseler J, Schortgen F, Dojat M, Cabello B, Bouadma L, Rodriguez P, Maggiore S, Reynaert M, Mersmann S, Brochard L. A multicenter randomized trial of computer-driven protocolized weaning from mechanical ventilation. *Am J Respir Crit Care Med* 2006; 15;174(8):894-900.
7. Sulemanji D, Marchese A, Garbarini P, Wysocki M, Kacmarek RM. Adaptive Support Ventilation: An Appropriate Mechanical Ventilation Strategy for Acute Respiratory Distress Syndrome? *Anesthesiology* 2009; 111(4):863-70.
8. Schädler, D, Miestinger, G, Becher, T, Frerichs, I, Weiler, N, Hörmann, C. Automated control of mechanical ventilation during general anaesthesia: study protocol of bicentric observational study (AVAS). *BMJ Open* 2017; 7e014742.
9. Rees SE, Karbing DS. Determining the appropriate model complexity for patient-specific advice on mechanical ventilation. *Biomed Tech (Berl)* 2017; 62(2):183-198.
10. Rees SE, Allerød C, Murley D, Zhao Y, Smith BW, Kjærgaard S, Thorgaard P, Andreassen S. Using physiological models and decision theory for selecting appropriate ventilator settings. Journal of Clinical Monitoring and Computing, 2006; Dec;20(6):421-429
11. Karbing DS, Allerød C, Thomsen LP, Espersen K, Thorgaard P, Andreassen S, Kjærgaard S, Rees SE. Retrospective evaluation of a decision support system for controlled mechanical ventilation. Med Biol Eng Comput. 2012 Jan;50(1):43-51.
12. Karbing DS, Allerød C, Thorgaard P, CariusAM, FrilevL, Andreassen S, Kjærgaard S, Rees SE. Prospective evaluation of a decision support system for setting inspired oxygen in intensive care patients. Journal of Critical Care, 2010, 25(3):367-74.
13. C Allerød, S.E Rees, B.S Rasmussen, D.S Karbing, S Kjærgaard, P Thorgaard, S Andreassen. A Decision Support System for suggesting ventilator settings: Retrospective evaluation in cardiac surgery patients ventilated in the ICU. Computer Methods and Programs in Biomedicine, 2008, vol. 92, nr. 2, s. 205-212
14. Karbing DS, Spadaro S, Dey N, Ragazzi R, Marangoni E, Dalla Corte F, Moro F, Lodahl D, Hansen NS, Winding R, Rees SE, Volta CA.. An Open-Loop, Physiologic Model-Based Decision Support System Can Provide Appropriate Ventilator Settings. Crit Care Med. 2018, 46(7):e642-e648

For peer review only

15. Spadaro S, Karbing DS, Dalla Corte F, Mauri T, Moro F, Gioia A, Volta CA, Rees SE. An open-loop, physiological model based decision support system can reduce pressure support while acting to preserve respiratory muscle function. J Crit. Care, 2018 Dec; 48:407-413.
16. S.E Rees, S Kjærgaard, P Thorgaard, J Malczynski, E Toft, S Andreassen. The Automatic Lung Parameter Estimator (ALPE) system: Non-invasive estimation of pulmonary gas exchange parameters in 10-15 minutes. Journal of Clinical Monitoring and Computing, 2002, Vol 17, No.1, pp 43-52.
17. Karbing DS, Kjærgaard S, Andreassen S, Espersen K, Rees SE. Minimal model quantification of pulmonary gas exchange in intensive care patients. Med Eng Phys. 2011 Mar;33(2):240-8.
18. S.E Rees, S Kjærgaard, S Andreassen, G. Hedenstierna. Reproduction of MIGET retention and excretion data using a simple model of gas exchange in lung damage caused by oleic acid infusion. Journal of Applied Physiology, 2006 Sep;101(3):826-32.
19. S.E Rees, S Kjærgaard, S Andreassen, G. Hedenstierna. Reproduction of inert gas and oxygenation data – a comparison of the MIGET and a simple model of pulmonary gas exchange. Intensive Care Medicine 2010;36:2117-2124.
20. D.S Karbing, S Kjaergaard, B.W Smith, K Espersen, C Allerod, S Andreassen, S.E Rees. Variation in the PaO2/FiO2 ratio with FiO2: Mathematical and experimental description, and clinical relevance. Critical Care. 2007; Vol. 11, No. 6.
21. Boles JM, Bion J, Connors A, Herridge M, Marsh B, Melot C, Peral R, Silverman H, Stanchina M, Vieillard-Baron A, welte T. Weaning from mechanical ventilation. Eur Respir J. 2007; 29:1033-1056.
22. Lellouche F, Mancebo J, Jolliet P, Roeseler J, Schortgen F, Dojat M, Cabello B, Bouadma L, Rodriguez P, Maggiore S, Reynaert M, Mersmann S, Brochard L. A multicenter randomized trial of computer-driven protocolized weaning from mechanical ventilation. Am J Respir Crit Care Med. 2006 Oct 15;174(8):894-900.

Authors' Contributions: MPV, DSK and SER conceived, designed and wrote the protocol. MPV drafted, reviewed and amended the manuscript. LM, edited figures, drafted R&D documents. JRW drafted Standard operating procedure, created original figures and drafted the initial protocol manuscript. SS, AG, LO, LM, JMC, SER and MPV reviewed and approved the final draft of the manuscript.

Funding Statement: This work was supported by Mermaid Care A/S and the National Institute of Health Research (NIHR).

Competing Interests Statement: SER and DSK are minor shareholders of Mermaid Care A/S who manufacture the Beacon Caresystem©. They have also acted as consultants for Mermaid Care A/S. SER is an unpaid board member of Mermaid Care A/S. Rest of the authors do not have competing interests to declare.

For peer review only

Figures legends/captions

Figure 1. Consort flow diagram for protocol.

Figure 2. Flowchart of BEACON Caresystem© intervention arm. Arterial Blood Gas (ABG)/ Automatic Lung Parameter Estimator (ALPE), Spontaneous Breathing Trial (SBT), Clinical Research Form (CRF).

For peer review only

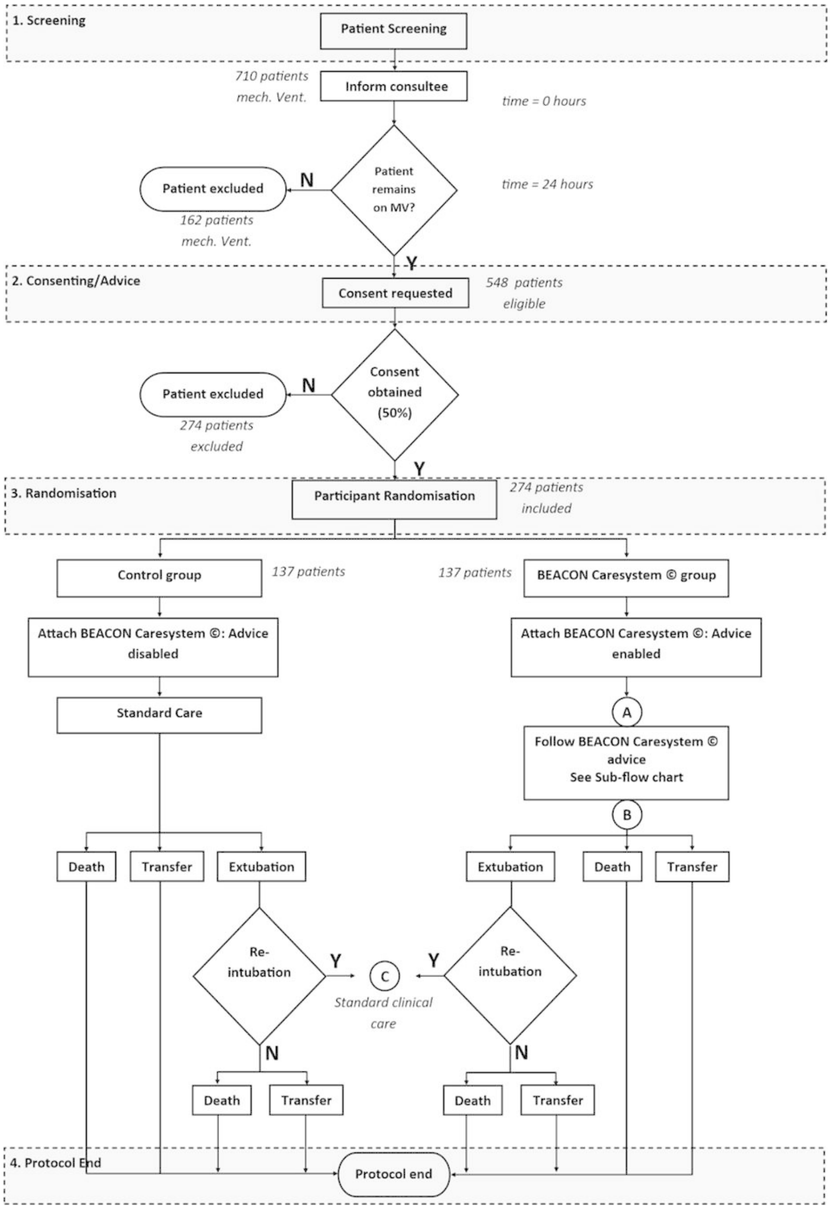

Consort flow diagram for protocol.

156x227mm (150 x 150 DPI)

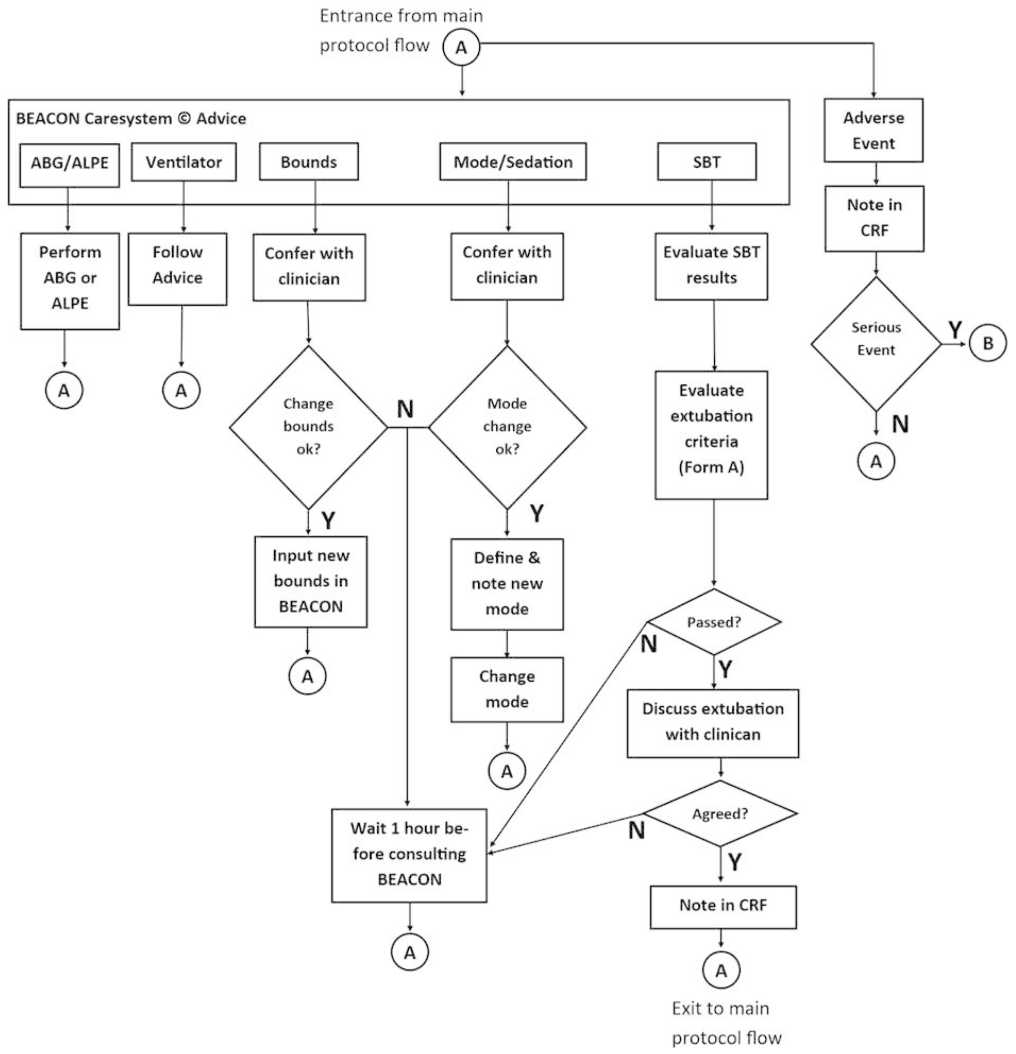

Flowchart of BEACON Caresystem© intervention arm. Arterial Blood Gas (ABG)/ Automatic Lung Parameter Estimator (ALPE), Spontaneous Breathing Trial (SBT), Clinical Research Form (CRF).

159x165mm (150 x 150 DPI)

SUPPLEMENTARY MATERIAL

The following is supplementary material for:

The iCareWean protocol on weaning from mechanical ventilation: A Single-blinded multi-centre randomised control trial comparing an open-loop decision support system and routine care, in the general Intensive Care Unit.

M. P. Vizcaychipi[1,5*],L. Martins[1], J.R. White[1], D.S Karbing[2], , A Gupta[3], S. Singh[1,5], L Osman[1], J. Moreno-Cuesta[4], S.E Rees[2].

1. Magill Department of Anaesthesia, Intensive Care Medicine and Pain Management, Chelsea and Westminster Hospital, C&W NHS Foundation Trust, London, UK.
2. Respiratory and Critical Care group (rcare). Department of Health Science and Technology. Aalborg University, Denmark.
3. Department of Anaesthesia and Intensive Care Medicine, West Middlesex University Hospital, C&W NHS Foundation Trust, London, UK
4. Department of Intensive Care Medicine, North Middlesex University Hospital, London, UK
5. Academic Department of Anaesthesia, Intensive Care & Pain Management, Imperial College London, UK.

**Corresponding Author: Dr Marcela P. Vizcaychipi, email: m.vizcaychipi@imperial.ac.uk*

For peer review only - http://bmjopen.bmj.com/site/about/guidelines.xhtml

The purpose of this supplementary material is to provide the reader with an understanding of the structure and function of the Beacon Caresystem©, described here as the clinical decision support system (CDSS). The material includes four sections describing: the mathematical models included in the system, including description of the models used to describe the effects of PEEP; the use of the system; a section illustrating the presentation of advice to the clinician; and a section illustrating screens relating to SBT and the extubation checklist. Much of the material presented here, including patient examples is taken from supplementary material from previous publications (1,2).

Mathematical models included in the CDSS

Figure E1 illustrates the structure of the mathematical models with a full description of model formulation and evaluation published recently (1,2,3). The system includes mathematical models of pulmonary gas exchange; respiratory mechanics; acid-base chemistry of blood, interstitial fluid, tissues and cerebral spinal fluid; respiratory drive and ventilation. In addition, the models include the effects of PEEP on gas exchange, pulmonary mechanics and ventilation as illustrated in figure E2 and described below. All models are tuned to the individual patient's physiological status through measurement of respiratory gas flows and pressures; calorimetry and capnography measurements of respiratory gas fractions of O_2 and CO_2, and subsequent calculation of oxygen utilisation (VO_2) and carbon dioxide production (VCO_2); pulse oximetry measurement of arterial oxygen saturation; and arterial blood measurements of acid-base, oxygenation and haemoglobin fractions. The model of pulmonary gas exchange is tuned to the appropriate matching of ventilation and perfusion to account for O_2 and CO_2 differences between arterial and end tidal gas values. To do so an arterial blood gas (ABG) is required on system start up. In some patients, the system also requires modification of FIO_2 to two levels for 2-5 minutes at each level to tune the pulmonary gas exchange model to the patient, a procedure previously called the automatic lung parameter estimator (ALPE) technique(3,4).

For peer review only - http://bmjopen.bmj.com/site/about/guidelines.xhtml

The respiratory mechanics model is tuned to dynamic compliance. The model of acid-base chemistry of the blood is tuned to measured values of arterial pH, PCO_2, PO_2, and SO_2, and haemoglobin concentration, with the acid-base chemistry of the cerebrospinal fluid (CSF) regulated to arterial bicarbonate values to account for chemical changes in respiratory drive. The respiratory drive model is tuned to describe the relationship between alveolar ventilation (VA) and arterial acid-base and oxygenation status. The ventilation model is tuned to the measured serial dead space (V*ds*) to allow calculation of alveolar ventilation. A series of algorithms are present in the CDSS to re-tune the models as new measurements present, or if the patient condition changes. These models are used to simulate the effect of changes in ventilator settings, with the system generating advice based upon simulated values.

For peer review only - http://bmjopen.bmj.com/site/about/guidelines.xhtml

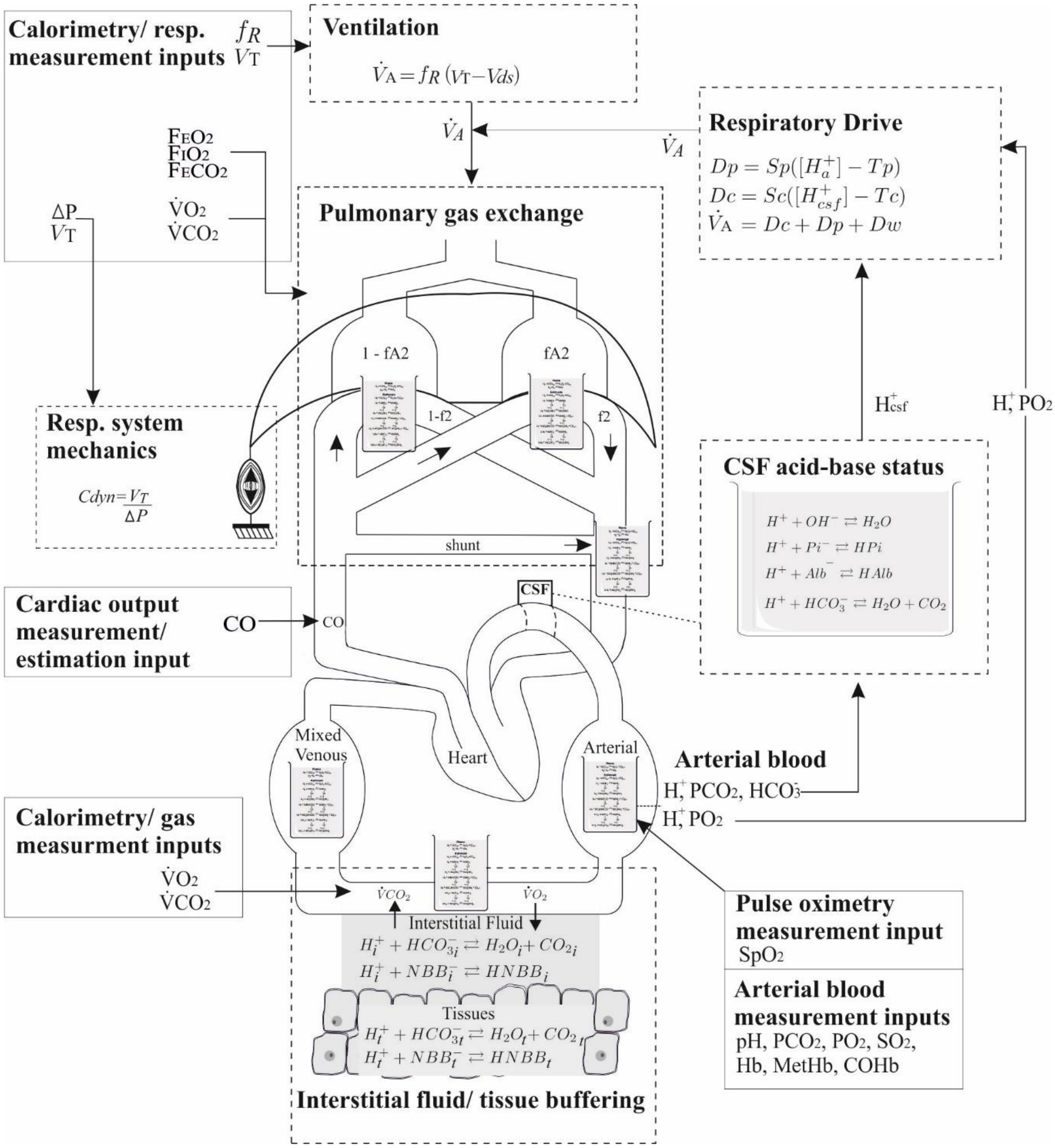

Figure E1: Mathematical models included in the CDSS, modified from (3). Mathematical models are indicated by dashed boxes with the exception of the acid-base model of blood, which is illustrated in each blood compartment as used. Measured input values are indicated by solid boxes.

For peer review only - http://bmjopen.bmj.com/site/about/guidelines.xhtml

Figure E2 illustrates the mathematical models built to describe the effects of PEEP. These are formulated as empirical linear models, with the system only advising on PEEP changes of a maximum of 3 cmH_2O in any step and the slope of these relationships modified automatically on measuring the response to PEEP. These models are integrated with those in figure E1 to enable simulation of the patient specific effects of PEEP and other ventilator settings simultaneously, enabling the system to advise on changing settings toward the best compromise for the individual patient given the model simulations.

For patients with acute respiratory failure, PEEP is usually set to improve gas exchange and lung mechanics, and the sub-figures A, B and C of figure E2 illustrate the baseline expected response to changes in PEEP of pulmonary shunt, respiratory system compliance and high ventilation/perfusion (V/Q). Changes in shunt, compliance and high V/Q automatically result in model-simulated changes in oxygenation, ventilation volumes and pressures, and carbon dioxide partial pressures through the models illustrated in figure E1. For shunt and compliance, the initial slopes of the models are dependent on the initial levels of shunt and compliance, with a greater improvement expected for a greater severity of respiratory abnormality. The slopes of these models are adapted automatically according to patient response to changes in PEEP, from measurements of oxygenation (SpO_2), respiratory volumes and pressures, and end tidal CO_2 values. Any increase in end tidal CO_2 values that are simulated to result in severe acidosis result in the system requesting an arterial blood gas. The decision to increase PEEP is then based on the potential benefit of improved oxygenation and respiratory volumes and pressures, given the tuning of the models to the patient's state. For patients responding poorly to increases in PEEP, the absolute slope of shunt and compliance models would be substantially reduced following measurement of the response to PEEP changes. The decision to reduce PEEP at high SpO_2 levels depends upon the FIO_2 level. FIO_2 will always be optimized first, such that the competing goals of sufficient oxygenation and oxygen toxicity are balanced. Following this, the system will calculate the likely effects on oxygenation and respiratory volumes and pressures on reducing PEEP from the models illustrated on subfigures A, B and C of figure E2, combined with the models of E1. For patients recovering from respiratory injury, without substantial pulmonary shunt

For peer review only - http://bmjopen.bmj.com/site/about/guidelines.xhtml

or low respiratory system compliance, the system will calculate that the potential negative effects of reducing PEEP will be outweighed by the positive effects of reducing plateau pressure.

For patients on pressure support ventilation without substantial problems related to gas exchange or respiratory system mechanics, PEEP is often used to support the respiratory muscles, preventing abnormal respiratory muscle activity as indicated by abnormal breathing patterns. Figure E2, D and E represent the empirical linear models included in the system to account for changes in tidal volume (VT) due to PEEP. Figure E2D illustrates two potential abnormal situations; the first is a very high VT at a low PEEP value, and the second a very low VT at a high PEEP, possibly representing the situation where the diaphragm may be over distended due to high PEEP. In these situations, the initial slope of the PEEP versus VT relationship is negative. Figure E2E illustrates the situation of a low tidal volume at a low value of PEEP, possible due to under-support of the respiratory muscles. In this situation, the initial slope of the PEEP versus VT relationship is positive. As with other PEEP models, all slopes are adapted following measurement of the response to PEEP changes.

For peer review only

For peer review only - http://bmjopen.bmj.com/site/about/guidelines.xhtml

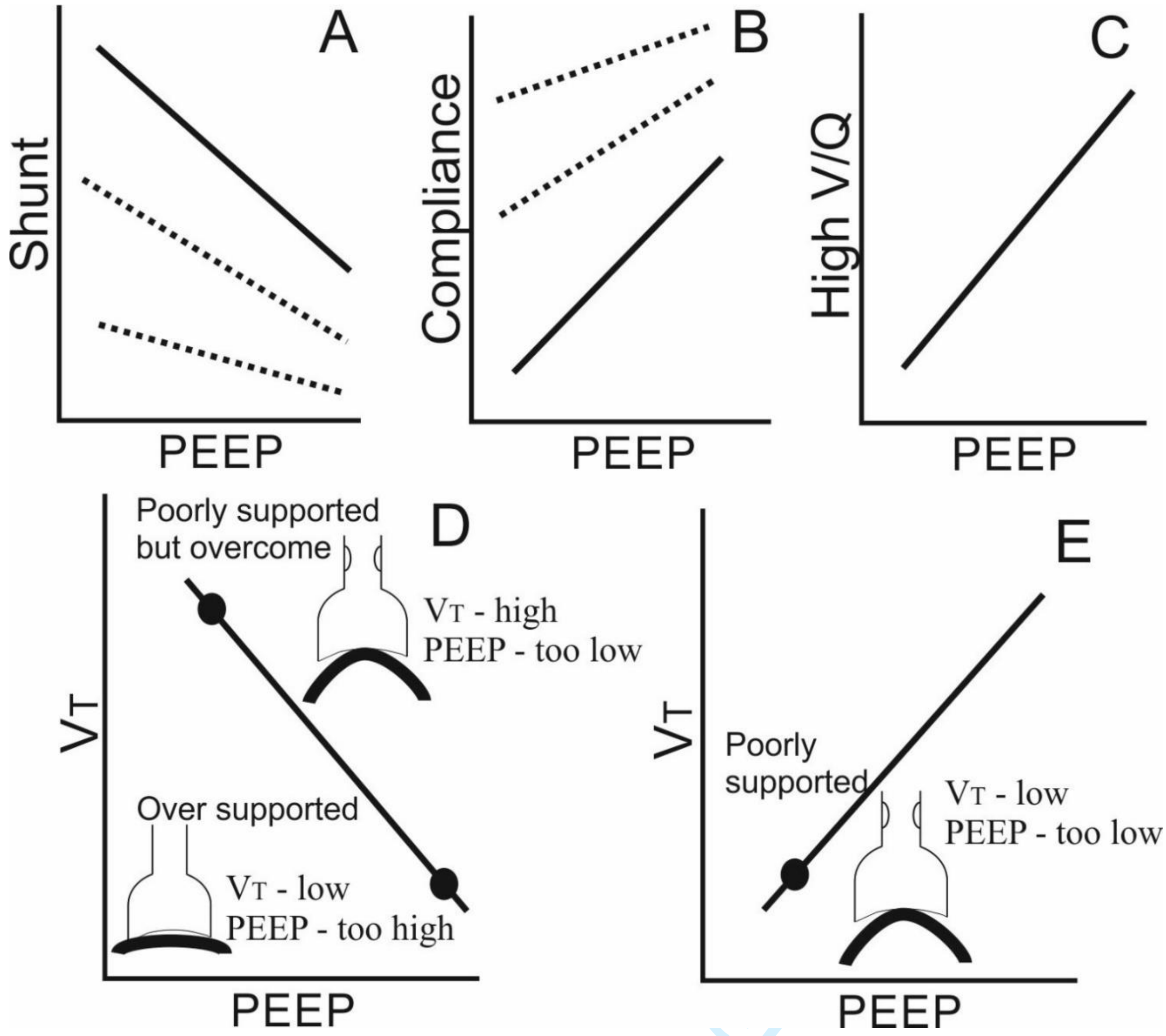

Figure E2: Mathematical models describing PEEP included in the Beacon Caresystem.

For peer review only - http://bmjopen.bmj.com/site/about/guidelines.xhtml

Use of the system

The system includes a tablet computer, plus a gas module including volumetric capnography and indirect calorimetry, with both mounted on the ventilator or a separate trolley. The patient is connected to the system via a respiratory tube measuring flow and with side stream sampling for measurement of O_2 and CO_2. The following text includes examples of advice generated by the system as reported in [1,2] beginning with the most simple examples. These examples are presentations of the screen of the tablet computer shown to the user.

In addition to these examples, it is important to note that on occasion the system will present advice to switch ventilator mode. This occurs if the patient is in a controlled ventilator mode and the system identifies a total respiratory frequency or a total minute ventilation that is significantly greater than that set, where the system provides a message to the user suggesting changing to support mode. Similarly, if the patient is in support mode and the model simulated pH is very low then the system provides a message asking the user to consider either control mode or reduction of anesthesia. In modes containing mandatory breaths initiated by the ventilator or by patient effort, and where different settings are available for patient initiated and ventilator initiated breaths, the system provides advice on these depending upon the pattern of patient ventilation, and whether the patient is currently in a period with substantial spontaneous breathing activity. The system functions on a variety of manufactures ventilators and, as illustrated in these examples, provides advice for a variety of modes. Current exceptions to these include APRV, and modes with automatic control of ventilator settings such as ASV© or SmartCare©. Data is collected automatically by the Beacon Caresystem, and includes tidal volumes, both set if the mode dictates, and also measured. The regulation of Vt and it relationship with mode can therefore be explored for both of the study arms. The system records all ventilator settings via connection to the ventilator, all direct measurements of flow and gas concentrations, its own SpO_2 measurement, and the timing and nature of all advice provided. The

For peer review only - http://bmjopen.bmj.com/site/about/guidelines.xhtml

extent to which advice has been followed and the effects of application of advice is therefore fully traceable.

For peer review only

For peer review only - http://bmjopen.bmj.com/site/about/guidelines.xhtml

Advice in pressure support mode ventilation.

Figure E3 illustrates a typical screen of the CDSS with advice to reduce PS and FIO_2. The hexagon on the right hand side of the screen visualizes the patient state in terms of the balances between over and under ventilation. The three vertical axes on the hexagon present the three balances between over and under oxygenation on the far right, (i.e. the compromise between the risks of oxygen toxicity and hypoxaemia, respectively); over and under ventilation as the central axis (i.e. the compromise between the risks of ventilator induced lung trauma and acidosis, respectively); and on the left the balance between over and under support (i.e. the balance between respiratory muscle atrophy due to over support, or patient stress due to under support). The blue symbol on the hexagon represents the patient's current state, and the grey symbol the simulated state according to the system's advice. The colour of the hexagon represents severity, with the most severe state represented as red, and the system automatically zooming in to yellow and green when possible. The left hand side of the screen shows both the current, simulated and advised settings. The white simulated blocks on the left are scroll wheels which allow the user to perform simulations of other levels of these ventilator settings.

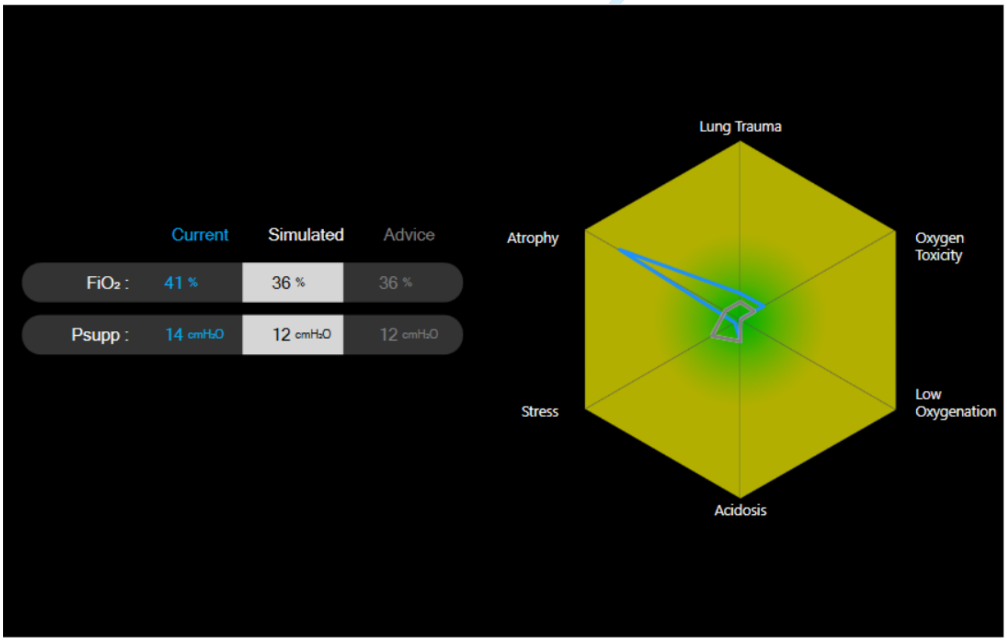

Figure E3 – User interface of the system, with advice to reduce FIO_2 and PS.

For peer review only - http://bmjopen.bmj.com/site/about/guidelines.xhtml

Figure E4 illustrates the same screen as E3, but with the corners of the hexagon activated to show the current, simulated and advised values of variables simulated by the physiological models. In this patient, the current FIO_2 setting of 41 %, results in a SaO_2 value of 97.9 % and a current simulated SvO_2 of 89.7 %. The system illustrates that the current balance between over and under oxygenation may be inappropriate with the blue symbol on the hexagon pointing slightly toward oxygen toxicity. The system therefore suggests reducing FIO_2. The system also illustrates an increased risk of respiratory muscle atrophy due to a low respiratory frequency, leading the system to suggest a reduction in PS from 14 to 12 cmH_2O, simulating that this may result in increased Rf.

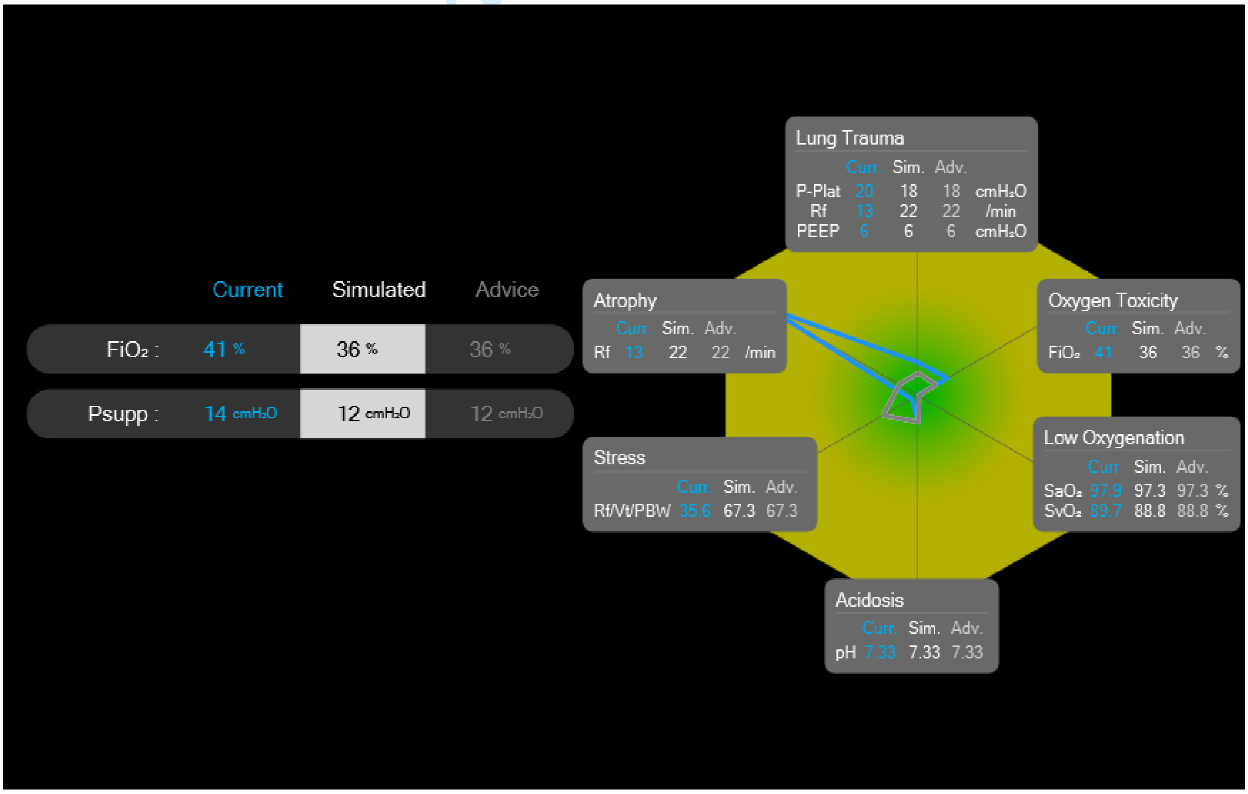

Figure E4 – User interface of the system, with advice to reduce FIO_2 and PS and corners of the hexagon activated to show model simulated physiological variables associated with the different conflicting clinical goals of mechanical ventilation.

For peer review only - http://bmjopen.bmj.com/site/about/guidelines.xhtml

Following several reductions in PS and FIO_2, the system advises on PEEP, illustrated in figure E5. The reduction of PS had less than the expected effect on increasing Rf, with the reduction in FIO_2 reducing oxygenation as expected. When exploring advice on PEEP, the CDSS also shows the simulated expected effect of PEEP, showing the simulated levels of shunt, low V/Q (ΔPO_2), high V/Q (ΔPCO_2), dead space (Vd) and dynamic compliance (COMP) at current, simulated and advised settings. The PEEP reduction was expected to have a limited effect on gas exchange parameters, with small increase in shunt, and a small decrease in dynamic compliance. As such, the advice was expected to result in a slight increase in Rf without a worsening in oxygenation.

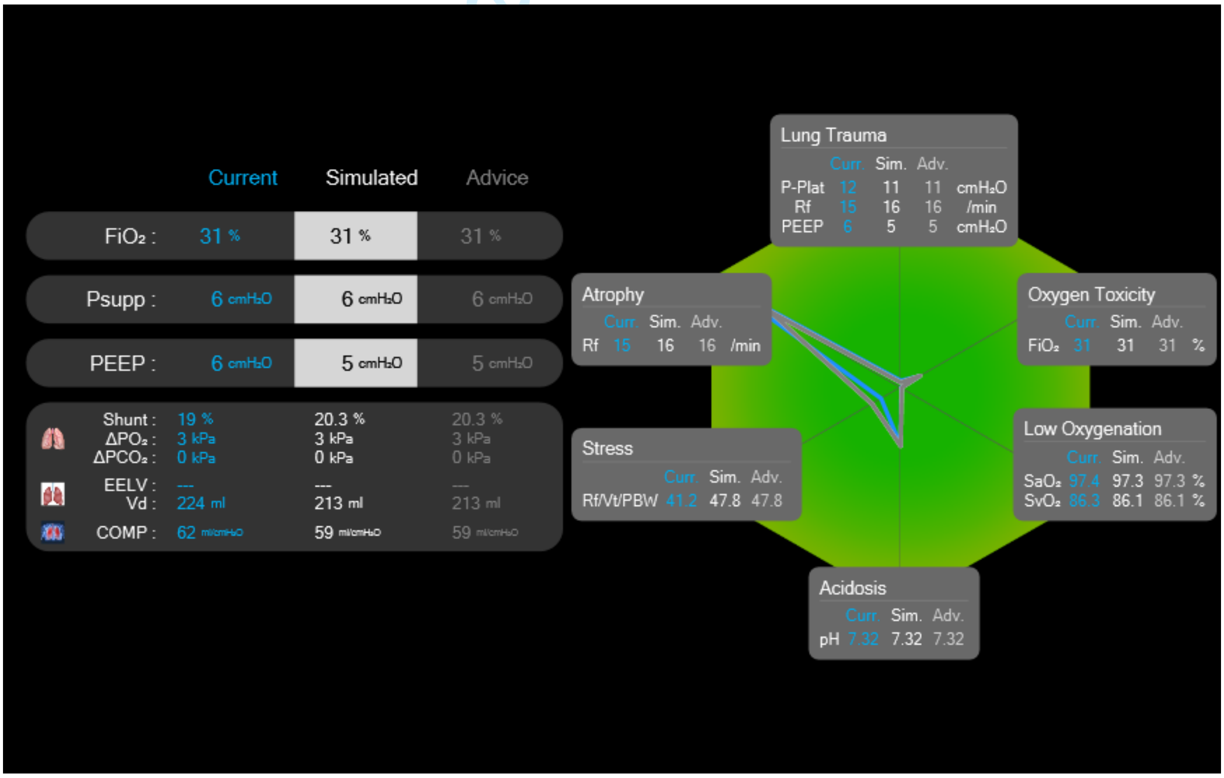

Figure E5 – User interface of the system, with an advice on PEEP.

For peer review only - http://bmjopen.bmj.com/site/about/guidelines.xhtml

Advice in control mode ventilaiton.

Figure E6 illustrates an advice including PEEP for a patient in pressure control ventilation. The vertical axis representing the balance between respiratory muscle atrophy and stress is disabled in controlled ventilation modes for cases where the patient has no spontaneous breathing activity. In this patient, the system indicated that the current balance between over and under ventilation might be inappropriate, with the blue symbol on the hexagon pointing toward lung trauma and with the patient being slightly alkalotic. The system suggested reducing PEEP and PC from 6 to 5 cmH_2O and 12 to 11 cmH_2O, respectively, and at the same time increasing Rf from 18 to 19 bpm. The simulated effect of PEEP was a negligible increase in pulmonary shunt, with simulated patient response to the combined advice being to reduce inspiratory pressure, reduce pH and maintain appropriate oxygenation.

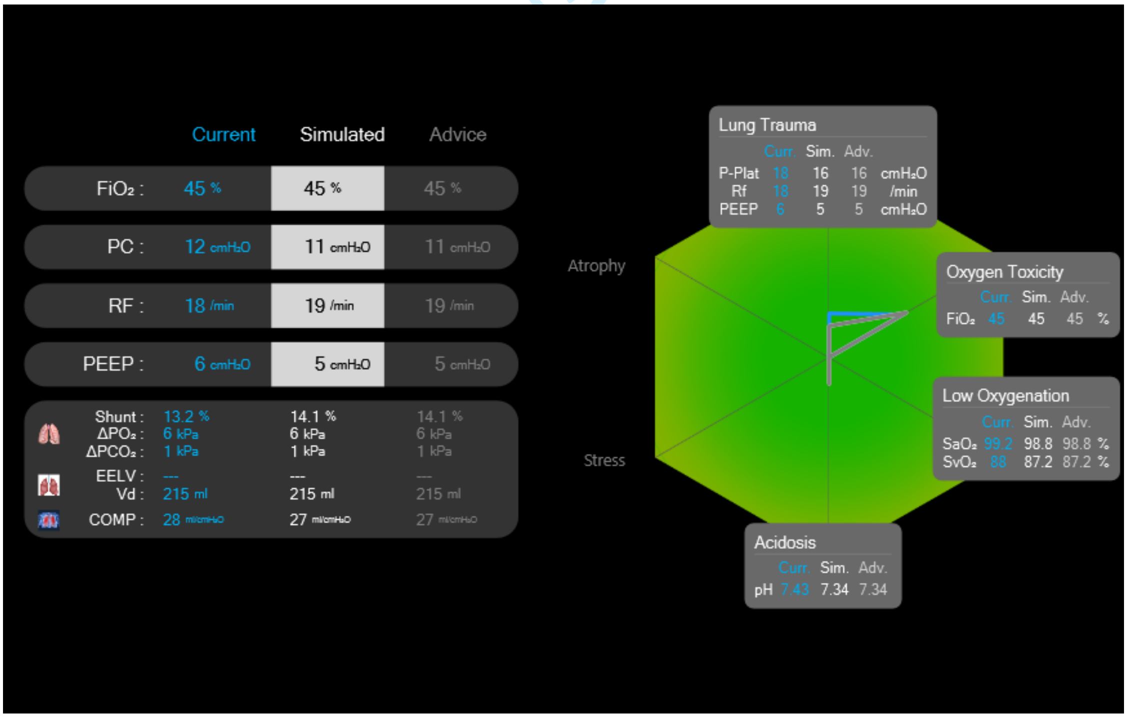

Figure E6 – User interface of the system illustrating an advice including PEEP in a patient ventilated in PC mode.

For peer review only - http://bmjopen.bmj.com/site/about/guidelines.xhtml

Figure E7 illustrates the result of following the advice illustrated in figure E8, and the subsequent advice provided by the system. The combined effect of PEEP and PC reduction and increase in Rf was as expected for both oxygenation and respiratory mechanics but less pronounced on pH. The advised level of PEEP was maintained in the next advice, but with an advice to decrease FIO_2 expecting a resulting safe oxygenation. Simulations in the details boxes at the hexagon corners take into account differences between set and measured Rf, where measured Rf was 17 bpm despite set Rf of 19 bpm.

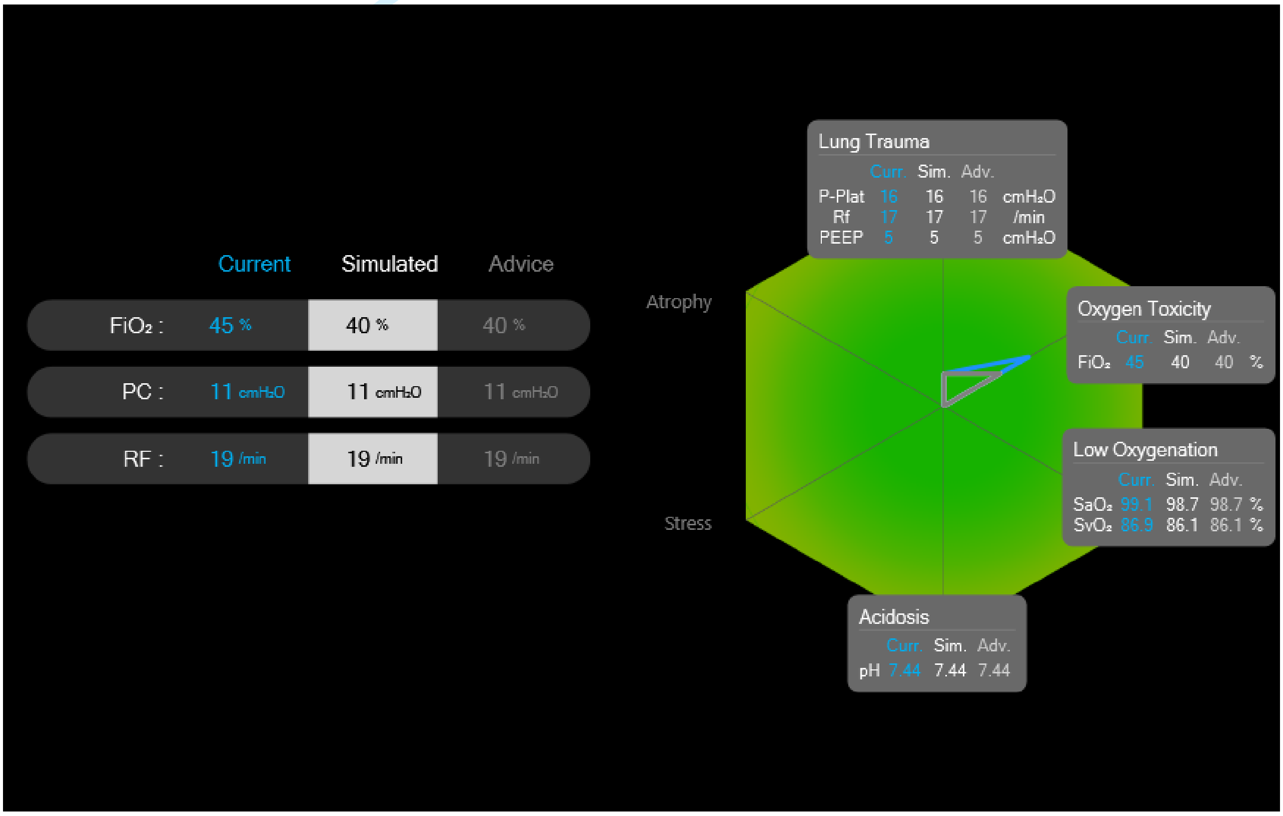

Figure E9 – User interface of the system, with results according to the advice in Figure E6, and the next advice.

For peer review only - http://bmjopen.bmj.com/site/about/guidelines.xhtml

SBT setting and review, and extubation check-list

For patients ventilated in pressure support mode with values of PEEP and PS below threshold values illustrated in figure E10, the system provides a counter on the screen which counts down from 30 minutes, and as such indicates that the patient has been within ventilator settings consistent with an SBT and stable with regard other respiratory measurements, for this duration. Cut-off values for these other variables are illustrated in figure E10

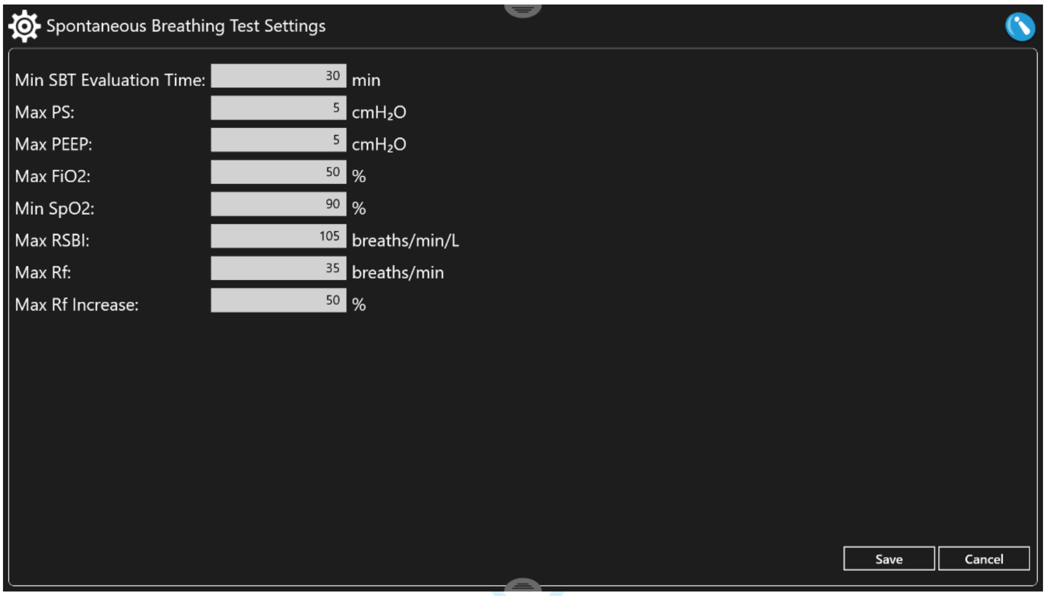

Figure E10 – User interface illustrating variables used to time the SBT counter of the system.

At any point when the counter is running, the user can view the screen illustrated in figure E11. This indicates which variables are within threshold, their values, and for how long this has been the case.

For peer review only - http://bmjopen.bmj.com/site/about/guidelines.xhtml

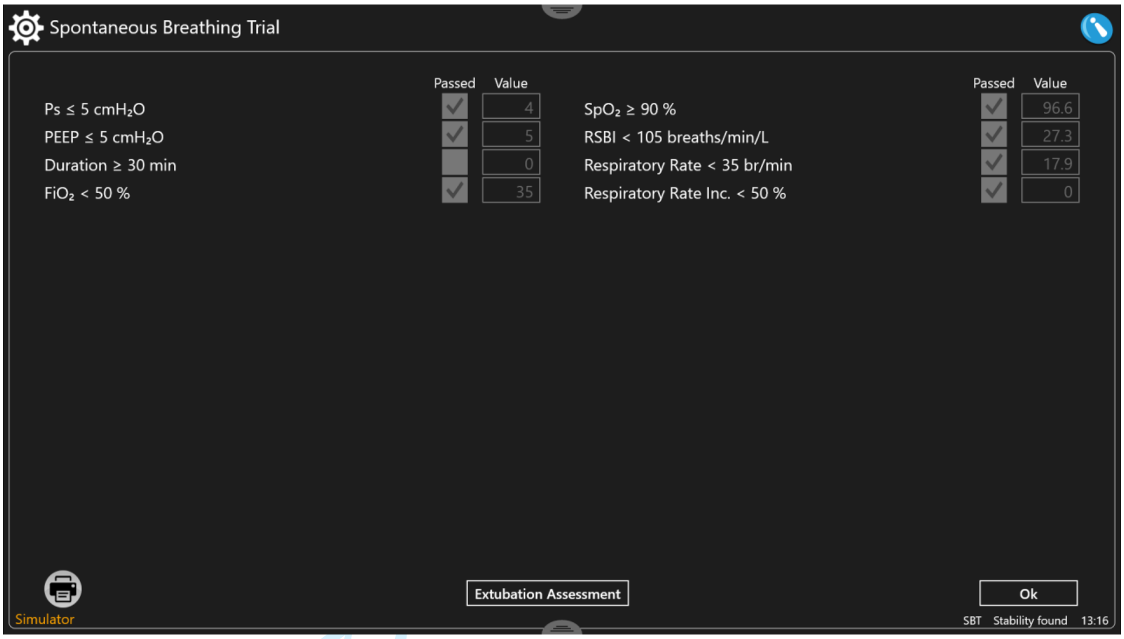

Figure E11 – User interface illustrating status of variables used to track the stability of patients over time in relation to the SBT counter.

When all variables illustrated in figure E11 are within range for the expected duration, the system allows checking of an extubation checklist illustrated in figure E12. Neither the SBT screens E10 and E11, nor the extubation checklist advise on an active SBT test or extubation.

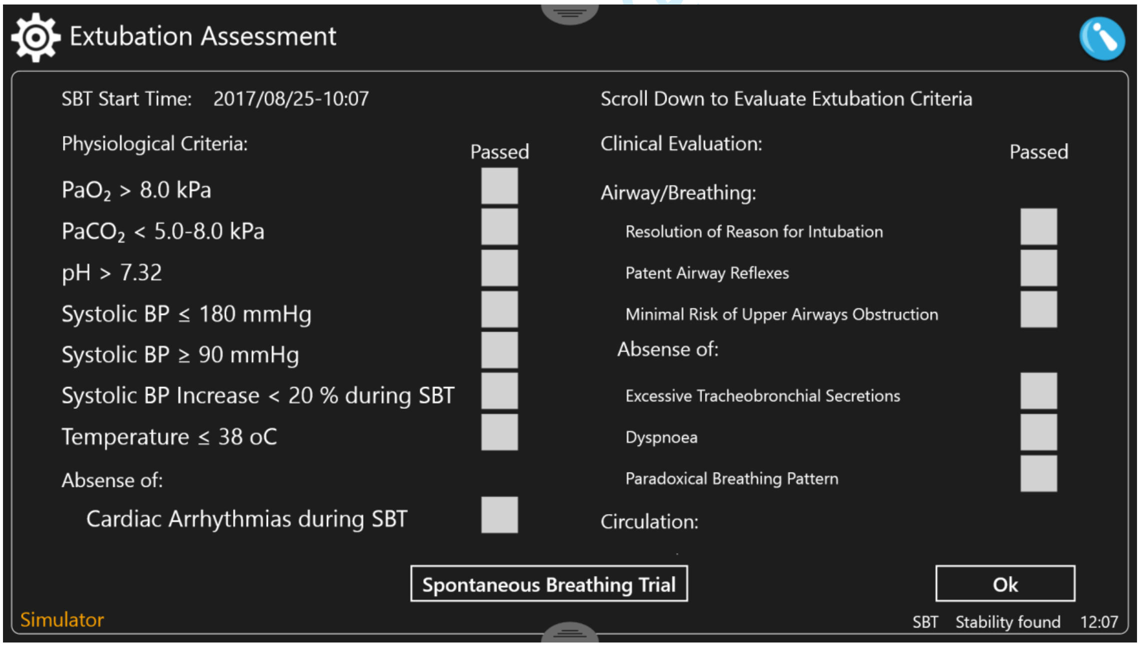

Figure E12a – Extubation checklist, top part of vertical scrolling window.

For peer review only - http://bmjopen.bmj.com/site/about/guidelines.xhtml

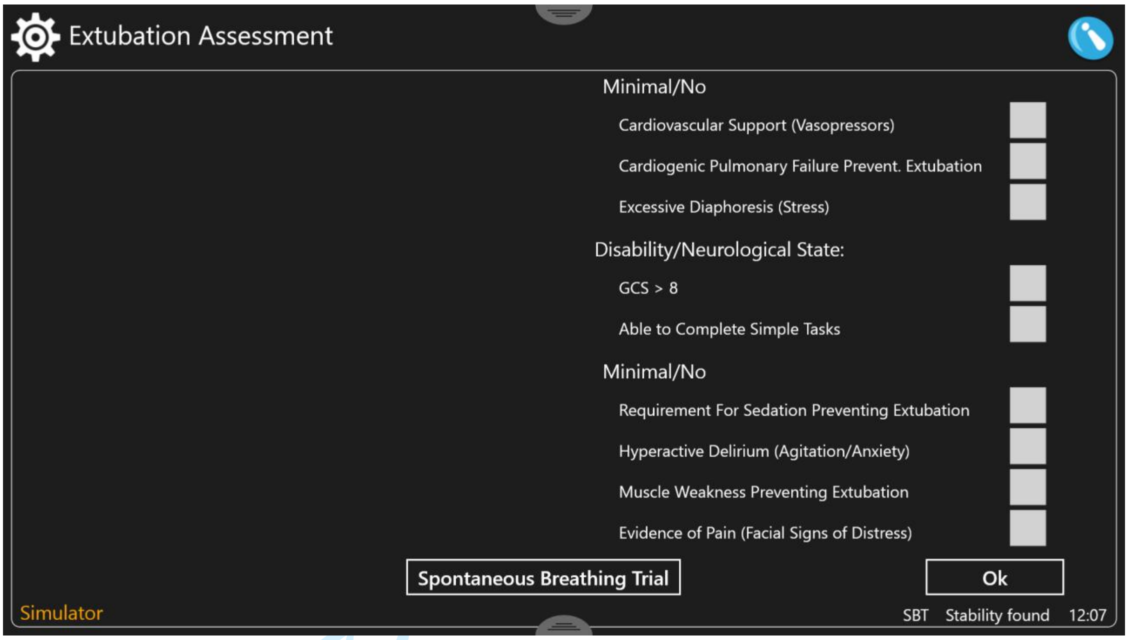

Figure E12b – Extubation checklist, bottom part of vertical scrolling window.

For peer review only - http://bmjopen.bmj.com/site/about/guidelines.xhtml

References

1. Karbing DS, Spadaro S, Dey N, Ragazzi R, Marangoni E, Dalla Corte F, Moro F, Lodahl D, Hansen NS, Winding R, Rees SE, Volta CA.. An Open-Loop, Physiologic Model-Based Decision Support System Can Provide Appropriate Ventilator Settings. Crit Care Med. 2018, 46(7):e642-e648

2. Spadaro S, Karbing DS, Dalla Corte F, Mauri T, Moro F, Gioia A, Volta CA, Rees SE. An open-loop, physiological model based decision support system can reduce pressure support while acting to preserve respiratory muscle function. J Crit. Care, 2018 Dec;48:407-413.

3. Rees SE, Karbing DS. Determining the appropriate model complexity for patient-specific advice on mechanical ventilation Biomed Tech (Berl). 201762(2):183-198.

4. Thomsen LP, Karbing DS, Smith BW, Murley D, Weinreich UM, Kjærgaard S, et al. Clinical refinement of the automatic lung parameter estimator (ALPE). J Clin Monit Comput. 2013;27(3):341-50.

For peer review only
